# The impact of Kamala Harris’s social identities during the 2024 presidential election

**DOI:** 10.1371/journal.pone.0354231

**Published:** 2026-07-24

**Authors:** Emily J. Brown, Sheba M. Aikawa, Evava S. Pietri, Tiffany A. Ito

**Affiliations:** Department of Psychology and Neuroscience, University of Colorado Boulder, Boulder, Colorado, United States of America; University of Stavanger, NORWAY

## Abstract

The social identities of Democratic Party presidential nominee Kamala Harris were noted frequently prior to the 2024 United States Election. We examined how making salient her marginalized racial and gender identities affected evaluations of her prior to the election. In Study 1, a stratified U.S. sample viewed a paragraph describing only Harris’s qualifications or her qualifications along with her gender, race, or both. Mentioning her race led to less favorable perceptions regardless of participants’ political orientation, suggesting subtle identity cues shape impressions. Political orientation did moderate responses when Harris was compared to an unnamed candidate, with more conservative participants rating Harris less favorably. Study 2 disaggregated Harris’s racial background showing that her Black identity, not her South Asian or combined racial identity, drove the negative evaluations across participant political orientation. As in Study 1, conservatives rated her more negatively than an unnamed candidate, but only when her Black identity was made salient. Together, these studies demonstrate that identity salience, particularly Black racial identity, can meaningfully influence candidate evaluations.

## Introduction

Kamala Harris was the first woman of Black and South Asian descent to be a major party’s presidential nominee in the United States (U.S.) yet her campaign was perceived as not making those identities a central aspect of her candidacy [1,2]. This perhaps reflected concern that her race and gender would be a liability for her campaign in part based on lessens from Barack Obama and Hillary Clinton’s presidential campaigns [3–5]. In fact, polling data showed that many voters saw Harris’s gender and race as a liability [5]. Rather than directly addressing her gender or racial identity, Harris spoke of it more obliquely. For example, in discussions of her mother, Harris would indirectly identify her own racial identity by noting her mom’s status as an immigrant but spent more time discussing her mom’s personal qualities of dedication and determination [6]. By contrast, the media often highlighted her race and/or gender [7], and her opponent Donald Trump frequently highlighted her racial heritage. One example occurred in July 2024, when Trump said “She was always of Indian heritage, and she was only promoting Indian heritage... I didn’t know she was Black until a number of years ago, when she happened to turn Black, and now she wants to be known as Black. So I don’t know, is she Indian or is she Black?” [7]. The vastly different approaches to discussing her identity raise the question of whether the different frames changed how she was perceived during her 2024 presidential candidacy.

Harris’s identities as a multiracial women suggest multiple possible influences of identity framing. One influence could occur via activation of stereotypical associations and the degree to which those associations are perceived as congruent with being president. According to Role Congruity Theory, group stereotypes determine how individuals are evaluated in social roles [8,9], including the role of U.S. president. When a group’s stereotype is consistent with the expectations for a role, individuals are evaluated more favorably as potential occupants compared to individuals from groups whose stereotypes are incongruent with the role. Both gendered and racialized stereotypes of leadership suggest that framing Harris in terms of either her gender or race could decrease her perceived suitability to serve as president. Political success is more strongly associated with stereotypically masculine attributes (e.g., leadership, strength) than with stereotypically feminine attributes (e.g., warmth, kindness) [8,10]. Gender-based role incongruity effects might be especially large for Harris given the unique demands of higher office such as foreign policy increasing perceived incongruity with being a woman [10]. Likewise, since White racial identity is associated with leadership [11,12] and Black candidates are perceived as less competent than White candidates [13], framing Harris in terms of her race may increase perceived incongruity with being president. Crucially, Harris is not only Black, but also South Asian. Unfortunately, there is limited research exploring perceptions of U.S. South Asian leaders or politicians, with most studies using the superordinate term Asian American without differentiating among different ethnic groups [12–14]. This makes it difficult to know how Harris will be evaluated when framed in terms of her South Asian identity in particular. Finally, framing that highlights both her race and gender could activate intersectional stereotypes [15,16]. Research on intersectionality shows that Black women are perceived as possessing qualities associated with leadership more than White women are [17,18] which might suggest greater role congruity when Harris is framed in terms of both race and gender than only in terms of gender.

Rather than operating via role congruence, other theorists have suggested that candidate demographic characteristics operate as heuristic cues signaling the candidate’s political attitudes, policy preferences, and ideological congruence with the voter [19–21]. Relevant to this mechanism is the perception that Black and women candidates are more liberal than their White and men counterparts [10,19,21–26], which should lead to different effects of framing depending on participant political orientation. Framings highlighting Harris’s race and/or gender should cue a liberal ideology and policy orientation, leading more liberal voters to perceive her more favorably and more conservative voters to perceive her less favorably, relative to when her race and/or gender are not emphasized.

Here we report two studies using Harris’s historic 2024 presidential candidacy to examine how identity frames of both race and gender — and their intersectional effects — influence evaluations of a U.S. presidential candidate. Framing effects assessed after Harris’ nomination for Vice President in 2020 and again after her inauguration in 2021 revealed mixed effects. Highlighting her race or gender had no impact on attitudes post-Vice Presidential nomination [27]. By contrast, shortly after she was inaugurated as Vice President, framing emphasizing either her gender, or both her Black racial identity and gender increased favorability. The authors attribute the different results to effects of context, pointing to the importance of re-examining framing effects on reactions to Harris under the context of her presidential campaign. To do so, we created a brief factual description of Harris noting her political accomplishments. We then varied whether aspects of her racial and/or gender identity were also mentioned. These identity cues also included brief contextual framing that reflected how such identities are commonly discussed in contemporary political discourse, including their perceived social and political implications [27–29]. We assessed effects on perceptions of her overall political abilities and a range of trait attributes. In Study 1, selected traits were associated with gender and racial stereotypes to determine if a race and/or gender framing led Harris to be perceived as congruent with stereotypes about race, gender, or intersectional race and gender stereotypes. In Study 2, we separately mentioned Harris’s Black or South Asian descent to assess their unique effects. Research on perceptions of South Asian politicians in the U.S. is limited, making this a critical contribution. Both studies also included a description of an unnamed politician with similar qualifications as a control condition. The Harris condition that omits any mention of social identities serves as a control by holding participants’ general attitudes toward Kamala Harris constant across experimental conditions, without making any specific aspect of her identity salient. However, because participants were likely already aware of Harris’s race and/or gender, these identity dimensions may still be implicitly activated, even in the absence of explicit cues. To address this, we included an additional control condition featuring a different political candidate. We intentionally avoided using a real politician, as preexisting attitudes toward known figures could confound interpretations of any observed differences relative to Harris. Instead, we developed an unfamiliar candidate with a background similar to Harris to provide a neutral comparison untainted by prior knowledge or bias. Both experiments were preregistered at https://osf.io/xn23q/overview?view_only=8cfaf19a7a7d4e49bbde922872b21748 and https://osf.io/cgpy8/overview?view_only=a4c2eeffc16548d08175597856b1d152. Deviations from pre-registration are noted in the main text and a fuller description of the reasons for deviation can be found at Study 1 Materials section of S1 File.

## Study 1 Method

### Participants

Participants were 598 Prolific users recruited between September 8th and September 10th, 2024, using Prolific’s representative sampling feature, which stratifies participants to approximate U.S. Census distributions on age, gender, and ethnicity, as well as political affiliation, to obtain a demographically diverse sample relevant to the U.S. voting population.

We used G*Power 3.1 [30] to determine the sample size needed to detect a small to medium effect size (f = 0.15) at α = .05 and β = .80 in a one-way ANOVA with 5 groups, resulting in *n* = 540. We aimed to collect 600 participants per our preregistration to allow for data loss due to inattention but stopped early to ensure the data were all collected prior to the Harris/Trump presidential debate held the evening of September 10^th^.

Inattentiveness was determined based on lack of variance in trait ratings, failing both explicit attention check items (e.g., *please select moderately if you are reading this*), or providing a non-responsive answer to the open-ended questions in the study. Lack of variance was considered more indicative of inattention in the trait ratings than other measures such as political abilities because of the heterogeneity of content among the traits; it seemed unlikely that an attentive participant would view Harris as identical on such a wide range of traits but more plausible they could find her equally competent on multiple political domains. Data from *n* = 5 were omitted for one or more indications of inattention. Although not a preregistered exclusion criterion, we also omitted data from 1 participant who reported not living in the United States as our inclusion criteria of a U.S. population was intended to recruit only from those living in the U.S. This resulted in a sample for analysis of 592 participants (295 women, 281 men, 14 non-binary individuals, and 2 individuals who preferred to self-describe, *M*_*Age*_ = 45.57, *SD*_*Age*_ = 15.79). The racial composition was 67.54% White, 12.95% Black, 4.88% East Asian, 9.01% Hispanic/ Latino/Latina/Latine/Latinx, 0.75% American Indian/Alaskan Native, 1.69% South Asian, and 2.44% other. Due to a typographical error, a racial category option was listed as “Middle Eastern or North American” (rather than North African) and was endorsed by 0.75% of participants. The sexual orientation composition was 80.91% Straight/Heterosexual, 7.43% Bisexual, 3.72% Gay/Lesbian, 2.53% Asexual, 1.35% Pansexual, 1.35% Queer, 0.51% preferred another term and self-identified, and 2.20% declined to report. Most participants (93.58%) were born in the United States and 95.10% were registered to vote. Participants reported their political ideology on a scale ranging from 1 (extremely liberal) to 7 (extremely conservative). As intended, the sample included participants across the ideological spectrum, with 10.1% identifying as extremely liberal, 22.5% as liberal, 13.0% as slightly liberal, 21.5% as moderate/middle of the road, 11.0% as slightly conservative, 15.9% as conservative, and 6.1% as extremely conservative. The mean level of political ideology was 3.73 (SD = 1.78). To examine ideological extremity, we computed an extremity score as the absolute distance from the scale midpoint (4), with higher values indicating greater ideological extremity. A linear regression comparing ideological extremity indicated that liberal participants (*M* = 1.94, *SD* = 0.71, *n* = 270) and conservative participants (*M* = 1.85, *SD* = 0.71, *n* = 195) did not significantly differ in extremity, *t*(461) = −1.29, *p* = 0.199, η²ₚ = .004. (Participants who selected the midpoint (4) were excluded from the assessment of extremity.)

A sensitivity analysis using G*Power [30] revealed that with a total sample size of *n* = 592, α = .05, and power = .80, the study was sufficiently powered to detect a minimum effect size of f = 0.14. This study was approved by the University of Colorado Boulder Institutional Review Board and conducted in accordance with the ethical standards for human subjects research. Informed consent was obtained electronically at the beginning of the study via a Qualtrics survey, where participants reviewed the consent information and indicated their agreement before proceeding.

### Procedure

Participants completed the study online via Prolific. Participants were randomly assigned to read one of five descriptions of a political candidate. In four of the conditions, participants read about Harris, with the description emphasizing key accomplishments of being the current Vice President and a former Senator and state Attorney General and advocating for civil rights, criminal justice reform, and healthcare access. The paragraphs differed by also naming (1) her race (2) her gender, (3) her race and gender or (4) neither her race nor gender*.* In the three conditions naming her race and/or gender, the historical implications of that identity were also noted similar to other studies of identity framing [31,32] (e.g., in the race framing condition “…*making history as the first Black and South Asian American to hold the office of Vice President…Her groundbreaking achievement represents a significant step forward for racial equality in U.S. leadership. Harris’s* *leadership reflects the increasing racial diversity in American political representation*). We held constant the description of Harris’s qualifications and political background in all conditions, with the race and gender frames adding identity-based information to the baseline description, an approach used in other research on identity framing [31]. We used the terms Black and South Asian American specifically because this is how Harris’s race was characterized in her official Vice-Presidential web page. In the final condition, (5) neither the race nor gender was specified for an unnamed politician described with a background similar to Harris (see Table 1 in S1 File for stimulus materials). Participants then rated the politician they saw (Harris or unnamed control politician) on traits, political abilities, and a warmth thermometer as well as reporting their own political orientation. They also answered the following questions not relevant to our primary hypotheses that are described in the Table 2 in S1 File: voting behavior, perceptions of Kamala Harris as a “DEI Hire,” perceptions of Harris’s race and open-ended questions including reasons to vote/not to voter for Harris and why they thought she was a “DEI hire” if they endorsed that option. Finally, participants reported their demographics.

### Materials

**Trait Ratings.** Traits were selected to assess the application of stereotypes associated with race, gender, and intersectional race and gender identity. Traits associated with competence were included because of their association with White Americans (*ambitious, competent, confident, and intelligent*; α = .84) [33,34]. Traits associated with communality were included because of their association with women (*friendly, kind, likeable, sensitive to the needs of others,* and *supportive*; α = .95) [34,35]. Traits associated with dominance were included because of their association with men and Black women (*bossy, controlling, demanding, dominant, assertive, powerful,* and *leader*; α = .65) [17,18,36]. Compared to many other racial and ethnic groups in the U.S., relatively little research has examined stereotypes of South Asians and examination of intersectional stereotypes of South Asian women are rare [37,38]. Studies that have been done often look at perceptions of competence and warmth, suggesting that the traits included here would be relevant to assessing the effects of making salient Harris’s South Asian heritage. To reduce social desirability, participants were asked to *“rate the extent to which you think people like you believe* ***Kamala Harris/the candidate*** *is…”* rather than providing their personal opinion [39,40], [37]. This indirect wording was intended to encourage more candid responding by prompting participants to consider how similar others might respond, rather than directly endorsing their own views [39]. Traits were rated on a scale from 1 (not at all) to 5 (extremely).

**Political Abilities.** Participants rated the degree to which they thought the candidate they were assigned to would manage situations relevant to politicians on a 1 (Strongly disagree) to 5 (Strongly agree) scale: *can handle crises effectively*, *connects with the public through their speeches and public appearances, clearly and concisely conveys their political views*, and *is knowledgeable about key political issues facing the country* (α = .95).

**Feeling Thermometer.** Participants rated their overall feelings towards the candidate they were randomly assigned to on a feeling thermometer scale of 0 (Cold) to 100 (Warm), with 50 labeled as Neutral.

**Participant Demographics**. Participants reported their age, gender identity, sexual orientation, employment status, racial identity, and highest level of education completed. They also were asked in what state and country they resided and in what country they were born. They reported their political orientation in response to the following question: *Here is a seven-point scale representing the political views people might hold, ranging from extremely liberal to extremely conservative. Where would you place yourself on this scale*? (1 = Extremely liberal – 7 = Extremely conservative).

### Exploratory and confirmatory factor analysis

To examine if the competence, communality, and dominance traits were perceived as separate dimensions, in part due to the low Cronbach’s alpha for dominance and similarity in results when the three dimensions were considered separately, exploratory factor analyses (EFAs) that were not preregistered were conducted using the psych R package [41]. Across the 16 traits, pairwise correlations ranged from *r* = −.50 −.86. To prepare for the EFA, eigenvalues were calculated, and scree plots were generated to determine the possible number of factors present based on Kaiser-Guttman rules [42]. Eigenvalues, scree plots and parallel analysis scree plots suggested 2 factors (see Tables 3 and 4 in S1 File for exploratory and confirmatory factor analysis results). EFAs were then run with Promax oblique rotation1 to examine the loadings and cross-loadings in a five-, four-, three-, and two-factor solution on polychoric-transformed items to account for their ordinal scales [43]. For each model, we examined how well-determined each factor was by looking at the number of single-factor loadings ≥ .55, following the recommended standard that there are at least as many “good” single-factor loadings per factor as there are estimated factors [42]. The two-factor solution was the best fit across the multiple criteria.

We followed with diagonal least weighted squares (DLWS) confirmatory factor analyses (CFAs) using the lavaan R package [44] on a three-factor model driven by theory (with factors for competence, communality, and dominance) and three different two factor models driven by both theory and the EFA. The two-factor models were (1) a theory-based content model in which competence and dominance items were combined into a single agency factor and communality items in a second factor, (2) a valence model based on the favorability of items (e.g., *friendly* vs *bossy)* and (3) a second valence model omitting *dominant* and *assertive* since they had moderately high loadings on both factors. The two-factor positive and negative model with *dominant* and *assertive* omitted had the best global fit based on RMSEA, CFI, and SRMR statistics. Thus, we are reporting the positive and negative factors (with dominant and assertive omitted) as they follow the same pattern of result as the original trait-based groupings. Analyses examining traits separated into the original competence, communality, and dominance factors are reported in Tables 10-12 in S1 File.

### Analytical strategy

Positive and negative traits, political abilities, and feeling thermometer were analyzed with separate regression analyses on each dependent variable, with four orthogonal contrast codes for the five experimental conditions: (1) Harris with mention of only race (race), (2) Harris with mention of only gender (gender), (3) Harris with mention of both race and gender (both), (4) Harris with no mention of race or gender (none), and (5) an unnamed candidate with similar qualifications (other). The race framing contrast code tested the effects of making Harris’s race salient (race = 1, gender = −1, both = 1, none = −1, and other = 0). The gender framing contrast code tested the effects of making Harris’s gender salient (race = −1, gender = 1, both = 1, none = −1, and other = 0). Another code tested the interaction between race and gender framing (race = −1, gender = −1, both = 1, none = 1, and other = 0). An additional code compared all Harris conditions to the other candidate (race = −1, gender = −1, both = −1, none = −1, and other = 4). Each model was run with political orientation (mean-centered; *M* = 3.73, *SD* = 1.78) as a moderator. If relevant, simple effects for liberals and conservatives were examined at +/- 1 standard deviation on the 1–7 scale, where 1 standard deviation above represented conservatives (*M* = 5.50) and 1 standard deviation below represented liberals (*M* = 1.95). Only significant effects are reported in the main text, with full inferential statistics report in Tables 6-9 in S1 File. We preregistered a different set of contrasts to examine the effects of race, gender, or both race and gender framing (e.g., both+race versus none to test effects of race framing) but opt to report these other contrasts for ease of interpretation because they correspond to tests of a main effect of race framing, a main effect of gender framing, and their interaction, with an additional contrast to compare the other condition to all Harris conditions. While these new contrasts we report are efficient because of their correspondence to a traditional ANOVA with 2 main effects and the interaction, they do not isolate particular contrasts that may be of interest, such as comparing race framing and gender framing separately to the none and control conditions. These were among our pre-registered codes and analyses using them show a similar pattern of results to the results reported here and are reported in Tables 17-20 in S1 File. In addition, while the contrasts presented here combined different conditions (e.g., the race and both conditions), descriptive statistics separately for each of the five conditions are presented in Table 5 in S1 File.

## Study 1 Results

The pattern of results was consistent across multiple measures so for ease of interpretation, we report results grouped by the contrast tested rather than the measure.

### Racial identity framing

The race contrast was significant across multiple measures, revealing that Harris was rated less favorably when her race and its historical implications were mentioned compared to when it was not. Specifically, the means and inferential statistics in Table 1 show that when her race was mentioned – alone or in conjunction with her gender – Harris was rated lower on positive traits, higher on negative traits, and less warmly on the feeling thermometer compared to when her race was not mentioned. Means were in the same direction of less favorable ratings of political abilities when race was mentioned but the race contrast was only marginally significant. None of the race effects were moderated by participant political orientation.

**Table 1:** Means and inferential statistics for contrast comparing when Harris’s Race was versus was not mentioned.

Measure	Salience of Racial Identity						
	*Race Mentioned (SE)*	*Race Not Mentioned (SE)*	*b* (SE)	*t* (582)	*p*	η²ₚ	95% CI
Positive Traits	3.31 (0.08)	3.51 (0.08)	−0.10 (0.04)	−2.65	.008	.012	−0.17, −0.03
Negative Traits	3.19 (0.08)	2.99 (0.09)	0.10 (0.04)	2.31	.021	.009	0.02, 0.18
Political Abilities	3.29 (0.10)	3.47 (0.11)	−0.10 (0.05)	−1.93	.054	.006	−0.19, 0.002
Feeling Thermometer	51.00 (2.33)	56.17 (2.51)	−2.57 (1.19)	−2.17	.031	.008	−4.91, −0.24

**Note.** *b* **=** unstandardized regression coefficient. Means are covariate adjusted means. SE = Standard Error. 95% CI = Confidence Intervals for *b. N* = 239 (Race Mentioned) and *N* = 237 (Race Not Mentioned)*.*

### Gender identity framing

The gender contrast was not significant for any measures, and it did not interact with participant political orientation for any measures.

### Interaction between race and gender identity framing

The contrast reflecting the interaction between making race and gender identity salient was not significant for any measures nor did it interact with participant political orientation for any measures.

### Harris vs. other candidate

The contrast comparing all Harris conditions to the other candidate was significant across multiple measures, revealing that Harris was rated less favorably than the other candidate. Specifically, the inferential statistics in Table 2 show that Harris was rated lower on positive traits, lower in political abilities, and less warmly on the feeling thermometer compared to the other candidate. However, each of these effects was moderated by participant political orientation. As shown in Table 3, tests of simple effects show the evaluative preference for the other candidate over Harris occurred only for more conservative but not more liberal participants.

**Table 2:** Inferential statistics for contrast comparing Harris vs. other candidate.

Measure	Harris vs. Other					Harris vs. Other X Political Identity				
	*b* (SE)	*t* (582)	*p*	η²ₚ	95% CI	*b* (SE)	*t* (582)	*p*	η²ₚ	95% CI
Positive Traits	0.07 (0.02)	3.80	<.001	0.024	0.03, 0.10	0.05 (0.01)	4.93	<.001	0.040	0.03, 0.07
Negative Traits	−0.01 (0.02)	−0.25	0.806	<.001	−0.04, 0.03	−0.01 (0.01)	−0.85	0.399	0.001	−0.03, 0.01
Political Abilities	0.12 (0.02)	5.54	<.001	0.050	0.08, 0.17	0.07 (0.01)	5.22	<.001	0.045	0.04, 0.09
Feeling Thermometer	1.46 (0.54)	2.721	0.007	0.013	0.41, 2.52	1.44 (0.31)	4.58	<.001	0.035	0.82, 2.06

**Note.** *b* **=** unstandardized regression coefficient. SE = Standard Error. 95% CI = Confidence Intervals for *b. N* = 476 (Harris) and *N* = 116 (Other Candidate)*.*

**Table 3:** Simple effects of Harris vs. other at 1 standard deviation above and below the mean on political identity.

Measure	1 Standard Deviation Below (Liberals)					1 Standard Deviation Above (Conservatives)				
	*b* (SE)	*t* (582)	*p*	η²ₚ	95% CI	b (SE)	*t* (582)	*p*	η²ₚ	95% CI
Positive Traits	−0.02 (0.03)	−0.891	0.373	0.001	−0.07, 0.03	0.15 (0.02)	6.40	<.001	0.066	0.11, 0.20
Negative Traits	0.01 (0.03)	0.43	0.671	<.001	−0.04, 0.69	−0.02 (0.03)	−0.81	0.420	0.001	−0.07, 0.03
Political Abilities	0.002 (0.03)	0.07	.792	<.001	−0.06, 0.07	0.25 (0.03)	7.86	<.001	0.096	0.18, 0.31
Feeling Thermometer	−1.10 (0.80)	−1.37	0.171	0.003	−2.67, 0.48	4.02 (0.75)	5.36	<.001	0.047	2.55, 5.50

**Note.** *b* **=** unstandardized regression coefficient. SE = Standard Error. 95% CI = Confidence Intervals for *b.* N = 476 (Harris) and *N* = 116 (Other Candidate).

### Political orientation

In addition to interacting with the Harris versus Other Candidate contrast, the partial effect of political orientation was significant for positive traits (*β* = −.32, *t*(582) = −16.65, *p* < .001, η²ₚ = .323), negative traits (*β* = 0.19, *t* (582) = 8.68, *p* < .001, η²ₚ = .115), poli*t*ical abilities (*β* = −0.43, *t* (582) = −17.22, *p* < .001, η²ₚ = .338), and feeling thermome*t*er ratings (*β* = −11.38, *t* (582) = −18.91, *p* < .001, η²ₚ = .381). These effects showed tha*t* regardless of condition, including whether Harris or the control politician were being rated, as political conservatism increased, participants gave lower ratings on positive traits, higher ratings on negative traits, and lower ratings on political abilities and the feeling thermometer.

## Study 1 Discussion

Data collected during Kamala Harris’s historic 2024 U.S. presidential campaign show that reference to Harris’s race decreased favorability. A descriptive paragraph with a race framing that noted her Black and South Asian descent and its historical implications — whether mentioned alone or in conjunction with her gender — resulted in less favorable impressions regardless of participant political orientation. While we selected traits based on specific politically relevant group stereotypes (e.g., that women are more communal than men, that Black women are more dominant than White women), factor analyses suggested that participants made global positive and negative evaluations rather than content-differentiated trait inferences, as reflected in trait positivity and negativity, and the feeling thermometer. This pattern suggests that race framing effects occurred via broad activation of favorability rather than activation of specific racial stereotypes.

Although participant political orientation did not moderate the impact of race framing, how Harris was evaluated compared to an unnamed politician described with a roughly comparable political background was moderated by political orientation. More conservative participants were more favorable to the unnamed candidate than Harris across multiple measures, but more liberal participants did not differentiate between the two candidates.

Contrary to predictions and to the emphasis often placed during the campaign on Harris’s gender [45], no effects of gender framing emerged. There was likewise no race by gender interactions, providing no evidence of intersectional effects on evaluations of Harris; implications of these null results are addressed in the general discussion.

While Study 1 showed significant effects of race framing, the observed effect sizes were small in magnitude. This may have occurred because participants had relatively well-formed opinions of Harris given her long political history and status as the current Vice President. We think it important to interpret the results within the context of the effect sizes but also note the practical significance of the results in showing that even with high profile candidates, small differences in the inclusion of race-related information can shift opinions.

## Study 2

The impact of mentioning Harris’s race on multiple measures in Study 1 begs the question of which aspect of her biracial identity was affecting perceptions. Were participants responding to the unique combination of her Black and South Asian heritage, more to her Black racial heritage, or more to her South Asian racial heritage? According to the Racial Positioning Model, racial and ethnic minority groups in the U.S. are evaluated along two distinct dimensions of perceived superiority/inferiority and Americanness/foreignness [46]. Of particular relevance to candidate Harris, African Americans and Asian Americans are perceived differently along these dimensions, with African Americans perceived as relatively American but inferior and Asian Americans as relatively superior but foreign. This conclusion comes from research focused broadly on Asian Americans rather than South Asian Americans in particular, but it raises the possibility that perceptions of Harris will differ depending on which aspect of her racial heritage is made salient. Research on hypodescent or the one-drop rule similarly suggests status differences for individuals of Black and Asian heritage. Perceptions of both Black-White and Asian-White biracial individuals are influenced by the status of their socially subordinate parent group, with biracial Black-White and Asian-White individuals perceived as relatively more Black or Asian than White [47,48]. However, the effects were bigger for Black-White than Asian-White individuals. Again, this work has not been done noting South Asian parentage so it is not clear how those with mixed South Asian ancestry are perceived in particular, but the extant research does suggest a lower relative status for Black than Asian ancestry in the U.S.

To test whether Harris’s Black heritage would more strongly affect perceptions than her South Asian heritage, as suggested by the Racial Positioning Model and research on hypodescent, we again had participants read either about Harris or the unnamed other candidate. Among those who read about Harris, the description varied in whether it named just her Black racial identity, just her South Asian racial identity, both her Black and South Asian racial identities, or neither her Black nor South Asian racial identities.

## Study 2 Method

### Participants

Participants were 592 Prolific users recruited using Prolific’s representative sampling feature, which stratifies participants to approximate U.S. Census distributions on age, gender, and ethnicity, as well as political affiliation. As in Study 1, we had a target *n* = 540 to achieve desired statistical power (f = 0.15, α = .05, β = .80) and preregistered a plan to collect data from *n* = 600 to allow for data loss. We deviated from this target because early elections were starting in some states, and we wanted data collection to occur before any participants may have voted. Data collection occurred between October 24^th^ to October 27^th^, 2024. We preregistered the same exclusion criteria as Study 1. However, due to a programming error all semantic trait pairs were coded in the same direction (i.e., all positive traits were on one side and all negative on the other). Given the partisan nature of the judgments, answering all items in the same way could plausibly reflect a consistent impression of Harris across all traits rather than inattention. We thus added ratings of political abilities to our consideration of straight line responding suggestive of inattention since political ability items were all coded in the opposite direction from the traits. Anyone straight-lining on traits was omitted from analyses only if they answered all political ability questions in a numerically consistent way (e.g., answering 5 for all trait and political ability items) since doing so indicates evaluatively inconsistent responses between the two measures. The new straight-lining criteria combined with the other two original exclusion criteria resulted in the omission of *n* = 3, leaving a sample for analysis of 588 participants (297 women, 183 men, 6 non-binary individuals, and 2 individuals who preferred to self-describe, *M*_*Age*_ = 45.69, *SD*_*Age*_ = 15.81). The racial composition was 70.33% White, 13.68% Black, 5.01% East Asian, 6.74% Hispanic/ Latino/Latina/Latine/Latinx, 0.77% American Indian/Alaskan Native, 1.54% South Asian, and 1.35% other. Due to a typographical error, a racial category option was listed as “Middle Eastern or North American” (rather than North African) and was endorsed by 0.39% of participants. The sexual orientation composition is 81.98% Straight/Heterosexual, 8.84% Bisexual, 4.33% Gay/Lesbian, 1.56% Asexual, 1.39% Pansexual, 0.69% Queer, 0.52% preferred another term and self-identified, and 0.69% declined to report. Of the 588 participants, 94.56% were born in the United States and 97.28% were registered to vote. Participants reported their political ideology on a scale ranging from 1 (extremely liberal) to 7 (extremely conservative). The sample included participants across the ideological spectrum, with 12.1% identifying as extremely liberal, 20.7% as liberal, 11.1% as slightly liberal, 21.3% as moderate/middle of the road, 13.1% as slightly conservative, 15.6% as conservative, and 6.1% as extremely conservative. The mean level of political ideology was 3.74 (*SD* = 1.81). We examined ideological extremity in the same way as Study 1, and in this sample, liberal participants were more ideologically extreme (*M* = 2.02, *SD* = 0.73, *n* = 258) than conservative participants (*M* = 1.80, *SD* = 0.21, *n* = 205), *t*(440.3) = −3.30, *p* = 0.001, η²ₚ = .023.

A sensitivity analysis using G*Power [30] revealed that with a total sample size of *n* = 588, α = .05, and power = .80, the study was sufficiently powered to detect a minimum effect size of f = 0.14. Study 2 received approval from the University of Colorado Boulder Institutional Review Board and was carried out in compliance with ethical standards for human subjects research. Participants completed an electronic informed consent process administered through Qualtrics, indicating their consent before accessing the survey materials.

### Procedure

Participants followed the same procedures as Study 1, with the following changes. Participants were randomly assigned to read a paragraph that included Harris’s Black racial identity, her South Asian racial identity, her Black and South Asian racial identities, neither her Black nor South Asian racial identities, or a paragraph about an unnamed politician with qualifications similar to Harris but whose race and gender were not named (see Table 34 in S1 File). Trait ratings were then made on semantic trait pairs as well as two items relevant to the Racial Positioning Model. Participants who read about the other candidate were next asked to report the age, gender, and race of the person they imagined. The majority of participants identified the candidate as a man (65.5%) and, independently, as White (51.7%). At the intersection, 41.4% specifically identified the candidate as a White man. All participants then answered ancillary measures not relevant to our primary hypotheses (described in Table 35 in S1 File) on their media consumption, perceived phenotypicality of Harris’s facial features, voting behavior, perceptions of Kamala Harris as a “DEI Hire,” perceptions of Harris’s race and open-ended questions including reasons to vote/not to voter for Harris and why they thought she was a “DEI hire” if they endorsed that option, and completed demographic measures.

### Materials

Political abilities (α = .95), the feeling thermometer, and the demographic questions were the same as Study 1. Measures that differed from Study 1 are described below.

**Traits.** To make sure results were not specific to the traits used in Study 1, especially since those traits were initially selected because of their relevance to racial and gender stereotypes, Study 2 selected traits broadly across dimensions of warmth and competence, viewed as fundamental trait dimensions in person perception [49]. Participants were asked to *“rate the extent to which you think people like you believe* ***Kamala Harris/the candidate*** *is…”* on 15 bipolar scales of *Competent/Incompetent, Intelligent/Unintelligent, Good Leader/Bad Leader, Friendly/Unfriendly, Kind/Unkind, Likeable/Unlikeable, Sensitive/Insensitive, Warm/Cold, Capable/Incapable, Hardworking/Lazy, Effective/Ineffective, Motivated/Lazy, Skilled/Unskilled, Sociable/Unsociable, and Pleasant/Unpleasant* on 5-point scales [49,50] (α = .98). Several Study 2 items directly overlap with Study 1 measures, including competent, intelligent, friendly, and kind. In addition, Study 1’s “sensitive to the needs of others” is captured as “sensitive” in Study 2, and “leader” is represented as “good leader/bad leader”. Bipolar scales were used because results on the positive and negative traits in Study 1 tended to merely be the inverse of each other rather than revealing unique patterns of effects. Combining positive and negative into a single bipolar scale allowed us to assess traits of interest more efficiently.

**Racial Positioning Measures.** Participants were asked to *“rate the extent to which you think people like you believe* ***Kamala Harris/the candidate*** *is…” Superior/Inferior* and *American/Foreign* on two 5-point bipolar scales adapted from Zou and Cheryan [33].

### Analytical strategy

We used a similar analytical strategy to Study 1, creating four orthogonal contrast codes for the five experimental conditions of (1) Harris with mention of only her Black racial identity (Black), (2) Harris with mention of only her South Asian racial identity (South Asian), (3) Harris with mention of both her Black and South Asian racial identities (both), (4) Harris with no mention of her Black or South Asian racial identities (none), and (5) an unnamed candidate with similar qualifications (other). The contrast codes test the effects of Black racial framing (Black = 1, South Asian = −1, both = 1, none = −1, and other = 0), South Asian racial framing (Black = −1, South Asian = 1, both = 1, none = −1, and other = 0), and the interaction between Black and South Asian racial framing (Black = −1, South Asian = −1, both = 1, none = 1, and other = 0). An additional code compared all Harris conditions to the other candidate (Black = −1, South Asian = −1, both = −1, none = −1, and other = 4). Each model included political orientation (mean-centered; *M* = 3.74, *SD* = 1.81) as a moderator. Simple effects for liberals and conservatives were examined at +/- 1 standard deviation on the 1–7 scale, where 1 standard deviation above represented conservatives (*M* = 5.55) and 1 standard deviation below represented liberals (*M* = 1.93). Only significant effects are reported in the main text (see Tables 37-41 in S1 File for full results). We preregistered a slightly different set of contrasts to examine the effects of making different aspects of Harris’s race salient (e.g., both+Black versus South Asian to test effects of Black racial framing) but opt to report these other contrasts for ease of interpretation because they correspond to tests of the main effects of Black and South Asian racial framing and their interaction. Analyses with the preregistered contrasts show a similar pattern of results (see Tables 42-47 in S1 File). As with Study 1, descriptive statistics separately for each of the five conditions are presented in the Table 36 in S1 File.

## Study 2 Results

As with Study 1, the pattern of results was consistent across multiple measures so for ease of interpretation, we report results grouped by the contrast tested rather than the measure.

### Black racial framing

The Black racial framing contrast was significant for trait favorability and the feeling thermometer. The means and inferential statistics in Table 4 show that when her Black racial identity was mentioned, Harris was rated less favorably on traits and less warmly on the feeling thermometer compared to when her Black racial identity was not mentioned. Means were in the same direction of less favorable ratings of political abilities when Black racial identity was mentioned but the Black racial framing contrast was only marginally significant.

**Table 4:** Means and inferential statistics for contrast comparing when Harris’s Black Racial identity was versus was not mentioned.

Measures	Salience of Black Racial Identity						
	*Black Racial Identity Mentioned (SE)*	*Black Racial Identity Not Mentioned (SE)*	*b* (SE)	*t* (578)	*p*	η²ₚ	95% CI
Trait Favorability	3.43 (0.09)	3.60 (0.09)	−0.08 (0.04)	−2.00	0.046	0.007	−0.17, −0.001
Political Abilities	3.23 (0.10)	3.41 (0.10)	−0.09 (0.05)	−1.90	0.058	0.006	−0.18, 0.003
Feeling Thermometer	50.93 (2.57)	56.15 (2.67)	−2.56 (1.24)	−2.07	0.039	0.007	−4.99, −0.13

**Note.** *b* **=** unstandardized regression coefficient. SE = Standard Error. 95% CI = Confidence Intervals for *b. N* = 238 (Black Racial Identity Mentioned) and *N* = 234 (Black Racial Identity Not Mentioned).

Although Black racial framing did not have a simple effect on ratings on the Superiority/Inferiority or American/foreign dimensions, the Black racial framing contrast did interact with political orientation for Superiority/Inferiority, *β = −*.05, *t* (578) *=* −2.02, *p* = .044, η²ₚ = .007. Tests of the simple Black racial framing contrast showed that among more conservative participants, Harris was rated lower in superiority when her Black racial identity was mentioned as compared to when it was not, *β = −*.17, *t* (578) *=* −2.65, *p* = .008, η²ₚ = .012. Among more liberal participants, ratings of superiority did not differ as a function of whether Harris’s Black racial identity was mentioned, *β =* .02, *t* (578) *=* 0.24, *p* = .810, η²ₚ < .001.

There was also a marginally significant interaction with political orientation for trait favorability, *β = −*.05, *t* (578) *=* −1.90, *p* = .058, η²ₚ = .006. Given the similar effect for Superiority/Inferiority, we examined the simple effects as a function of political orientation. More conservative participants rated Harris less favorably when her Black racial identity was made salient compared to when it was not (*β* = −0.17, *t* (578) = −2.78, *p* = .006, η²ₚ = .013), but the simple effect of the Black racial identity contrast was not significant for liberals (*β* = −0.004, *t* (578) = −0.06, *p* = .954, η²ₚ < .001).

### South Asian racial framing

The South Asian contrast was not significant for any measure but it did interact with participant political orientation for the rating of Superiority/Inferiority, *β = −*.05, *t* (578) *=* −1.98, *p* = .048, η²ₚ = .007. Tests of the simple South Asian racial framing contrast showed that more liberal participants rated Harris higher in superiority when her South Asian racial identity was mentioned as compared to when it was not, *β =* .13, *t* (578) *=* 2.03, *p* = .043, η²ₚ = .007. Among more conservative participants, ratings of superiority did not differ as a function of whether Harris’s South Asian racial identity was mentioned, *β =* −0.05, *t* (578) *=* −0.77, *p* = .444, η²ₚ = .001.

### Interaction between Black and South Asian racial framing

The contrast reflecting the interaction between making Harris’s Black and South Asian racial framing was not significant for any measures and it did not interact with participant political orientation for any measures.

### Harris vs other candidate

As seen in Table 5, participants rated Harris less favorably on traits, lower on political abilities, less warmly on the feeling thermometer, less superior, and less American compared to the other candidate. Each effect was moderated by political orientation and for each measure the Harris vs other simple effect was significant only for more conservative participants (see Table 6). Thus, more conservative participants rated Harris less favorably on traits, lower on political abilities, less warmly on the feeling thermometer, less superior, and less American compared to the other candidate but more liberal participants did not.

**Table 5:** Inferential statistics for contrast comparing Harris vs. other candidate.

Measure	Harris vs. Other					Harris vs. Other X Political Identity				
	*b* (SE)	*t* (578)	*p*	η²ₚ	95% CI	*b* (SE)	*t* (578)	*p*	η²ₚ	95% CI
Trait Favorability	0.13 (0.02)	6.67	<.001	0.072	0.09, 0.16	0.07 (0.01)	6.52	<.001	0.069	0.05, 0.09
Political Abilities	0.19 (0.02)	8.83	<.001	0.119	0.15, 0.23	0.08 (0.01)	6.90	<.001	0.076	0.06, 0.10
Feeling Thermometer	2.89 (0.56)	5.18	<.001	0.044	1.80, 3.99	1.74 (0.30)	5.74	<.001	0.054	1.14, 2.33
Superiority	0.09 (0.02)	4.40	<.001	0.032	0.05, 0.13	0.06 (0.01)	4.85	<.001	0.039	0.03, 0.08
Americanness	0.07 (0.02)	3.38	.001	0.019	0.03, 0.11	0.05 (0.01)	4.12	<.001	0.028	0.02, 0.07

**Note.** *b* **=** unstandardized regression coefficient. SE = Standard Error. 95% CI = Confidence Intervals for *b*. *N* = 472 (Harris) and *N* = 116 (Other Candidate).

**Table 6:** Simple effects of Harris vs. other at 1 standard deviation above and below the mean on political identity.

Measure	1 Standard Deviation Below (Liberals)					1 Standard Deviation Above (Conservatives)				
	*b* (SE)	*t* (578)	*p*	η²ₚ	95% CI	b (SE)	*t* (578)	*p*	η²ₚ	95% CI
Traits	0.01 (0.03)	0.21	.838	<.001	−0.05, 0.06	0.25 (0.03)	9.07	<.001	0.125	0.20, 0.30
Political Abilities	0.04 (0.03)	1.52	.129	0.004	−0.01, 0.10	0.33 (0.03)	10.82	<.001	0.169	0.27, 0.39
Feeling Thermometer	−0.25 (0.76)	−0.33	0.741	<.001	−1.74, 0.74	6.03 (0.80)	7.50	<.001	0.089	4.45, 7.61
Superiority	−0.01 (0.03)	−0.26	.792	<.001	−0.11, 0.05	0.19 (0.03)	6.36	<.001	0.065	0.13, 0.25
Americanness	−0.01 (0.03)	−0.48	0.630	<.001	−0.07, 0.04	0.16 (0.03)	5.15	<.001	0.044	0.10, 0.21

**Note.** *b* **=** unstandardized regression coefficient. SE = Standard Error. 95% CI = Confidence Intervals for *b*. *N* = 472 (Harris) and *N* = 116 (Other Candidate).

### Political orientation effects

In addition to interactions already discussed with specific contrasts, the partial effect of political orientation was significant in the analyses for trait favorability (*β* = −.39, *t*(578) = −18.73, *p* < .001, η²ₚ = .378), political abilities, (*β* = −.43, *t*(578) = −18.58, *p* < .001, η²ₚ = .374), feeling *t*hermometer (*β* = -.11.02, *t*(578) = −17.94, *p* < .001, η²ₚ = .358), superiority (*β* = −.31, *t*(578) = −13.53, *p* < .001, η²ₚ = .241), and foreignness (*β* = −.25, *t*(578) = −11.01, *p* < .001, η²ₚ = .173). Similar to Study 1, these effec*t*s showed that regardless of condition, including whether Harris or the control politician were being rated, as political conservatism increased, participants gave less favorable trait ratings, lower ratings on political abilities, were less warm on the feeling thermometer, and rated their candidate as less superior and less American.

## Study 2 Discussion

Study 1 showed that Harris was perceived less favorably when both aspects of her biracial identity were made salient compared to when racial identity and its historical implications were not explicitly named. By separately referencing her Black and South Asian identity in Study 2, we found that favorability was primarily affected by Harris’s Black racial identity. Ratings of trait favorability and warmness on the feeling thermometer were lowered only by mentioning her Black racial identity and not by mentioning her South Asian racial identity. This occurred across participant political orientation.

The different positioning of African Americans and Asian Americans at the group level along the dimensions of superiority and Americanness in the Racial Positioning Model [46] led us to add those as explicit measures. Broadly consistent with the Racial Positioning Model in which African Americans are viewed as relatively low in superiority, making salient Harris’s Black racial identity led more conservative participants to rate her as less superior compared to when her Black racial identity was not mentioned. Within the racial positioning model, Asian Americans as a group are rated as relatively high in superiority. Here we find that among more liberal participants only, Harris was rated more superior when her South Asian racial identity was named as compared to when it was not. In addition, in comparison to the other candidate (whom participants tended to assume was a White man), Harris was viewed as relatively less superior and American among more conservative participants. These results show an extension of the Racial Positioning Model to evaluations of an individual and that such perceptions are sensitive to target racial salience and participant political orientation.

## General Discussion

Across two studies during the 2024 presidential campaign, we examined how subtle shifts in how Kamala Harris was described influenced judgments central to presidential candidate evaluations. In Study 1, referencing both aspects of her biracial identity led to less favorable ratings compared to when her race was not mentioned. The race effect occurred regardless of whether her gender was mentioned. Study 2 isolated the different aspects of her racial identity, revealing that it was specifically Harris’s Black racial identity, rather than her South Asian identity or the combination of both identities, that led to less favorable evaluations. None of these race framing effects on evaluative responses were moderated by participant political orientation, showing similar evaluative decreases following reference to Harris’s Black racial identity across the political spectrum. Moreover, results show a valence-based rather than stereotyped based effect. That is, making her racial identity salient resulted in an overall less favorable impression of Harris, rather than selectively increasing activation of traits stereotypical of her racial identity.

In contrast to the consistent effects of race framing in both studies, there was no effect of gender framing in Study 1. Similarly, there was no evidence in Study 1 that participants evaluated Harris through an intersectional lens as a Black and South Asian woman. We doubt that either finding means gender played no role in voter’s response to Harris, or that she and other candidates are not evaluated intersectionally. One explanation for the lack of differences in ratings when Harris’s gender was or was not mentioned may be chronic salience. Comparisons between polling data in the Harris and 2016 Hillary Clinton presidential campaigns show that voters viewed being a woman to be an even greater liability for Harris than Clinton [51]. It is possible that Harris’s gender was highly salient, even in conditions where gender was not mentioned. In addition, such chronic salience of Harris’s gender identity could have contributed to the less favorable ratings of Harris relative to the other candidate obtained in both studies, especially since Study 2 showed most participants saw the other candidate as a man.

Regarding the lack of intersectional effects, it is possible Harris’s biracial identity and/or her particular racial identities contributed to this lack of effect. Limited research on the cultural stereotypes Americans hold about South Asian Americans [52,53] makes it unclear to what degree consensual stereotypes exist. If a strong consensual stereotype and associated evaluations is lacking, this may have made it unlikely that participants for whom both race and gender was made salient in Study 1 perceived Harris in a uniquely intersectional way that incorporated her South Asian heritage. More generally, how a multiracial identity is integrated into an intersectional identity along with other social categories such as gender is not known. The particulars of Harris’s candidacy may have also played a role. Discussions of her social identities tended to focus on *either* her race or gender, perhaps making it less likely that participants would consider her in an intersectional way. Additionally, research and theorizing have suggested that for Black women, race is the dominate identity, with perceivers typically viewing and judging Black women through the lens of their race [54–56]. Thus, it is possible that making salient Harris’s Black identity, even when in conjunction with her gender or South Asian identity, made her Black identity the most salient and fundamental identity. Ultimately, this Black identity was perceived as misaligned with positive traits and political attributes.

In addition to race effects in Studies 1 and 2, both studies also showed that Harris was evaluated less favorably than an unnamed politician. We do not find it surprising that someone about whom very little is known is perceived more favorably than someone with a long and varied political career. As already noted, that most participants assumed the unnamed other candidate was a man may also indicate this difference partially reflects gender bias. What is perhaps more interesting is that the greater favorability of the other candidate was moderated by political identity. Simple effects tests showed that only more conservative participants favored the other candidate over Harris. This pattern is consistent with theories that demographic characteristics heuristically cue a politician’s ideological congruence with the voter. It suggests that regardless of whether the description highlighted her race and/or gender, Harris cued specific political attitudes and policy preferences and/or general ideological incongruence for more conservative voters [13,19,20].

Our results converge with a prior study on identity framing in showing shifts in how Harris was perceived when her Black identity was highlighted but no effects when highlighting her South Asian identity (in that study, they used the broader label of *Asian)* [27]. However, in that study, Black framing *increased* the favorability of opinions about Harris whereas favorability decreased in two studies here. There are critical differences between studies that may account for the different outcomes. Perhaps of greatest relevance, the data in Clayton et al. (2023) were collected in January 2021, shortly after Harris was inaugurated as Vice President. The effects of Black racial identity in evaluating politicians may have changed in the intervening three years. As another possibility, the impact on voters of highlighting Harris’s Black racial identity may have changed when she was running for president herself compared to when she was being evaluated as a Vice President (with a White man as the president). We cannot determine the cause of the different results but think they highlight the dynamic and context-sensitive effects of identity frames that warrants further consideration.

### Limitations

Although the pattern of data are consistent with voters interpreting candidate demographic features as signaling their political attitudes, policy preferences, and ideological congruence with the voter, these studies were not able to identify the specific mechanism. We also note that we elected to present the same core biographical and credential information about Harris in all conditions so that any differences could not be attributed to a reduction in the absolute quantity of qualification-related information. At the same time, we recognize that participants may have responded to differences in framing and identity salience in multiple ways, and the design does not allow us to fully disentangle heuristic-cuing accounts from alternative interpretations such as reactions to the relevance or emphasis of identity in political communication. We also note that the findings may be specific to a particular political candidate and time period. Framing effects may differ for other candidates and contexts, particularly when the candidate is less established or when identity-related issues are discussed differently by the candidate themselves, their opponent, and/or the media. Furthermore, the present design was not intended to isolate intersectional effects by separating identity salience from accompanying evaluative framing. Instead, identity was embedded within broader descriptive and evaluative cues, consistent with its typical presentation in campaign materials and media coverage. Future research could build on this work by experimentally separating identity salience from such framing to more precisely identify the mechanisms underlying intersectional effects. We were also not able to examine the role of media coverage in reinforcing or dampening these effects. Nevertheless, we think it is important that even in the context of an ongoing campaign in which prospective voters were being bombarded with information about Harris, slight differences in how Harris was characterized had the ability to shift how Americans evaluated her. Finally, we opted to both name Harris’s various identities and note their historical implications [32] rather than testing their separate effects. While this left us unable to determine if merely naming the identity and (briefly) noting its implications have dissociable effects, we did so because the discourse surrounding the campaign frequently both noted Harris’s identities and their implication [1,45,57]. This increases the mundane realism of the studies.

Observed effect sizes were generally small in magnitude. This may be attributable to Harris being a highly familiar political figure, making large shifts in evaluations due to a situational manipulation unlikely. We powered the studies to detect small-to-moderate effects (f = .15) and sensitivity analyses indicate that the current sample sizes provide adequate power to detect effects of approximately f ≥ .14. However, we note that some smaller effects of making Harris’s race or gender salient may not have been detectable, warranting caution in interpreting weaker or null findings. We do think it is notable that even given Harris’s high visibility, both studies show race framing effects, illustrating that how sources chose to frame her could have shifted recipients’ perceptions.

Additionally, although the outcome measure used an indirect “people like you” framing intended to reduce social desirability concerns, this wording may introduce some variability in how participants interpret the reference group. Accordingly, results should be interpreted as reflecting indirectly elicited evaluations rather than direct self-reported attitudes.

Finally, we considered whether the greater political extremity in Study 2 could account for the larger number of effects moderated by political ideology in Study 2 than Study 1. We cannot rule this out but do not think this is obviously the explanation for difference in results between the studies. For one reason, a substantial and similar number of participants in both studies reported they were moderate or middle of the road (21.% and 21.3%). Second, the different manipulations between the two studies may account for the different number of effects of political ideology. Moderating effects of political ideology on making either Harris’s Black or South Asian racial identity salient may only occur when those identities are separately mentioned (as in Study 2) but not when together (as in Study 1). There could also be a history effect that made liberals and conservatives react differently to reminders about Harris’s racial identity in the two studies given the data were collected during a fast-moving political campaign. Finally, we note that in Study 2, when political ideology moderated an effect of making one of Harris’s racial identity salient, this was usually due to more conservative but not liberal participants showing an effect of racial framing. That is, the simple racial framing effect in three of four cases was significant for conservative but not liberal participants. If extremity accounted for interactions with political ideology in Study 2, we would expect the simple effects of condition to be more likely for liberal than conservative participants. Taken together, while we do not think differences in ideological extremity between liberal and conservative participants accounts for the observed moderation effects, results of the two studies should nevertheless be interpreted taking the slightly greater ideological extremity among liberal participants in Study 2 into account.

## Conclusion

In a historic U.S. presidential campaign that featured the first biracial woman to be a candidate of a major party, our data reveal some of the complexity of how her identities were perceived by prospective voters, demonstrating that identity framing —particularly Black racial identity—can influence candidate evaluations. The Harris campaign was perceived as purposely downplaying her racial and gender identities to avoid invoking divisiveness. Our results on the one hand suggest that concern about emphasizing her racial identity – and her Black racial identity in particular – may have been well-placed. On the other hand, we cannot know how prospective voters would have responded to a more explicit and nuanced integration of her racial and/or gender identity into her campaign. Our results sadly also suggest the benefits of attacking a political opponent’s racial and gender identities. One notable period of the 2024 presidential campaign involved opponents questioning Harris’s Black heritage [7]. While this may be perceived as distancing Harris from her Black heritage, our results suggest that the many defenses explaining that Harris is a Black American that it initiated could have had a negative evaluative impact by making salient ideological associations with that racial identity.

## Supporting information

S1 File Supplemental materials and analyses for Study 1 and 2. This file contains supplemental experiment materials, additional measures, exploratory and confirmatory factor analyses, preregistered contrast codes analyses, and supplemental inferential statistics for Studies 1 and 2 (Tables 1–66).
(DOCX)

S2 File Human Participants Research Checklist Harris. (PDF)
