# Supplementary material for: The impact of Kamala Harris’s social identities during the 2024 presidential election
Source: PLoS One. 2026 Jul 24;21(7):e0354231. doi: 10.1371/journal.pone.0354231 (PMC13399341; doi:10.1371/journal.pone.0354231)
Supplement: S1 File — This file contains supplemental experiment materials, additional measures, exploratory and confirmatory factor analyses, preregistered contrast codes analyses, and supplemental inferential statistics for Studies 1 and 2 (Tables 1–66). (DOCX) [file pone.0354231.s001.docx]

**The Impact of Kamala Harris’s Social Identities During the 2024 Presidential Election**

**Supplemental Materials**

Emily J. Brown, Sheba M. Aikawa, Evava S. Pietri, and Tiffany A. Ito

Department of Psychology and Neuroscience, University of Colorado Boulder

# Contents

[Contents 2](#_Toc232336180)

[Supplemental Study 1 Materials 5](#_Toc232336181)

[Table 1. Study 1 Priming Paragraph Text 7](#_Toc232336182)

[Table 2. Study 1 Additional Measures 8](#_Toc232336183)

[Supplemental Study 1 Analyses 10](#_Toc232336184)

[Table 3. Study 1 Exploratory Factor Analysis Loadings for Two-Factor Extraction 12](#_Toc232336185)

[Table 4. Study 1 Confirmatory Factor Analysis Fit Statistics 13](#_Toc232336186)

[Table 5. Study 1 Descriptive Statistics for Outcomes by Condition 14](#_Toc232336187)

[Table 6. Study 1 Inferential Statistics for Positive Ratings by Condition and Political Orientation 15](#_Toc232336188)

[Table 7. Study 1 Inferential Statistics for Negative Ratings by Condition and Political Orientation 16](#_Toc232336189)

[Table 8. Study 1 Inferential Statistics for Feeling Thermometer by Condition and Political Orientation 17](#_Toc232336190)

[Table 9. Study 1 Inferential Statistics for Political Abilities Ratings by Condition and Political Orientation 18](#_Toc232336191)

[Table 10. Study 1 Inferential Statistics on Competence Traits by Condition and Political Orientation 19](#_Toc232336192)

[Table 11. Study 1 Inferential Statistics on Communality by Condition and Political Orientation 20](#_Toc232336193)

[Table 12: Study 1 Inferential Statistics on Dominance Traits by Condition and Political Orientation 21](#_Toc232336194)

[Table 13. Study 1 Preregistered Contrast Codes 22](#_Toc232336195)

[Table 14. Study 1 Inferential Statistics for Preregistered Contrasts on Competence Ratings by Condition and Political Orientation 23](#_Toc232336196)

[Table 15. Study 1 Inferential Statistics for Preregistered Contrasts on Communality Ratings by Condition and Political Orientation 24](#_Toc232336197)

[Table 16. Study 1 Inferential Statistics for Preregistered Contrasts on Dominance Ratings by Condition and Political Orientation 25](#_Toc232336198)

[Table 17. Study 1 Inferential Statistics for Preregistered Contrasts on Positive Ratings by Condition and Political Orientation 26](#_Toc232336199)

[Table 18. Study 1 Inferential Statistics for Preregistered Contrasts on Negative Ratings by Condition and Political Orientation 27](#_Toc232336200)

[Table 19. Study 1 Inferential Statistics for Preregistered Contrasts on Feeling Thermometer by Condition and Political Orientation 28](#_Toc232336201)

[Table 20. Study 1 Inferential Statistics for Preregistered Contrasts on Political Abilities by Condition and Political Orientation 29](#_Toc232336202)

[Table 21. Study 1 Inferential Statistics for Performance as a Political Leader by Condition and Political Orientation 30](#_Toc232336203)

[Table 22. Study 1 Inferential Statistics for Perceptions of Harris as Black by Condition and Political Orientation 31](#_Toc232336204)

[Table 23. Study 1 Inferential Statistics for Perceptions of Harris as South Asian by Condition and Political Orientation 32](#_Toc232336205)

[Table 24. Study 1 Inferential Statistics for Harris’s Perceptions of Herself as Black by Condition and Political Orientation 33](#_Toc232336206)

[Table 25. Study 1 Inferential Statistics for Harris’s Perceptions of Viewing Herself as South Asian by Condition and Political Orientation 34](#_Toc232336207)

[Table 26. Study 1 Inferential Statistics for Positive Traits by Condition and Participant Gender 35](#_Toc232336208)

[Table 27. Study 1 Inferential Statistics for Negative Traits by Condition and Participant Gender 36](#_Toc232336209)

[Table 28. Study 1 Inferential Statistics for Feeling Thermometer by Condition and Participant Gender 37](#_Toc232336210)

[Table 29. Study 1 Inferential Statistics for Political Abilities by Condition and Participant Gender 38](#_Toc232336211)

[Table 30. Study 1 Inferential Statistics for Positive Traits by Condition and Participant Race 39](#_Toc232336212)

[Table 31. Study 1 Inferential Statistics for Negative Traits by Condition and Participant Race 40](#_Toc232336213)

[Table 32. Study 1 Inferential Statistics for Feeling Thermometer by Condition and Participant Race 41](#_Toc232336214)

[Table 33. Study 1 Inferential Statistics for Political Abilities by Condition and Participant Race 42](#_Toc232336215)

[Supplemental Study 2 Materials 43](#_Toc232336216)

[Table 34. Study 2 Priming Paragraph Text 45](#_Toc232336217)

[Table 35. Study 2 Additional Measures 46](#_Toc232336218)

[Supplemental Study 2 Analyses 49](#_Toc232336219)

[Table 36. Study 2 Descriptive Statistics for Outcomes by Condition 50](#_Toc232336220)

[Table 37. Study 2 Inferential Statistics for Trait Ratings by Condition and Political Orientation 51](#_Toc232336221)

[Table 38. Study 2 Inferential Statistics for Thermometer Ratings by Condition and Political Orientation 52](#_Toc232336222)

[Table 39. Study 2 Inferential Statistics for Political Abilities by Condition and Political Orientation 53](#_Toc232336223)

[Table 40. Study 2 Inferential Statistics for Superiority/Inferiority Ratings by Condition and Political Orientation 54](#_Toc232336224)

[Table 41. Study 2 Inferential Statistics for American/Foreign Ratings by Condition and Political Orientation 55](#_Toc232336225)

[Table 42. Study 2 Preregistered Contrast Codes 56](#_Toc232336226)

[Table 43. Study 2 Inferential Statistics for Preregistered Contrasts on Trait Ratings by Condition and Political Orientation 57](#_Toc232336227)

[Table 44. Study 2 Inferential Statistics for Preregistered Contrasts on Thermometer Ratings by Condition and Political Orientation 58](#_Toc232336228)

[Table 45. Study 2 Inferential Statistics for Preregistered Contrasts on Political Abilities by Condition and Political Orientation 59](#_Toc232336229)

[Table 46. Study 2 Inferential Statistics for Preregistered Contrasts on Superiority/Inferiority Ratings by Condition and Political Orientation 60](#_Toc232336230)

[Table 47. Study 2 Inferential Statistics for Preregistered Contrasts on American/Foreign Ratings by Condition and Political Orientation 61](#_Toc232336231)

[Table 48. Study 2 Inferential Statistics for Performance as a Political Leader by Condition and Political Orientation 62](#_Toc232336232)

[Table 49. Study 2 Inferential Statistics for Perceptions of Harris as Black by Condition and Political Orientation 63](#_Toc232336233)

[Table 50. Study 2 Inferential Statistics for Perception of Harris as South Asian by Condition and Political Orientation 64](#_Toc232336234)

[Table 51. Study 2 Inferential Statistics for Harris’s Perceptions of Herself as Black by Condition and Political Orientation 65](#_Toc232336235)

[Table 52. Study 2 Inferential Statistics for Harris’s Perceptions of Herself as South Asian by Condition and Political Orientation 66](#_Toc232336236)

[Table 53. Study 2 Inferential Statistics for Other’s Perceptions of Black Americans as Superior/Inferior by Condition and Political Orientation 67](#_Toc232336237)

[Table 54. Study 2 Inferential Statistics for Other’s Perceptions of Black Americans as American/Foreign by Condition and Political Orientation 68](#_Toc232336238)

[Table 55. Study 2 Inferential Statistics for Other’s Perceptions of South Asian Americans as Superior/Inferior by Condition and Political Orientation 69](#_Toc232336239)

[Table 56. Study 2 Inferential Statistics for Other’s Perceptions of South Asian as American/Foreign by Condition and Political Orientation 70](#_Toc232336240)

[Table 57. Study 2 Inferential Statistics for Perceptions of Afrocentricity by Condition and Political Orientation 71](#_Toc232336241)

[Table 58. Study 2 Inferential Statistics for Perceptions of South Asian Features by Condition and Political Orientation 72](#_Toc232336242)

[Table 59. Study 2 Inferential Statistics for Traits by Condition and Gender 73](#_Toc232336243)

[Table 60. Study 2 Inferential Statistics for Feeling Thermometer by Condition and Gender 74](#_Toc232336244)

[Table 61. Study 2 Inferential Statistics for Political Abilities by Condition and Gender 75](#_Toc232336245)

[Table 62. Study 2 Inferential Statistics for Superior/Inferior by Condition and Gender 76](#_Toc232336246)

[Table 63. Study 2 Inferential Statistics for American/Foreign by Condition and Gender 77](#_Toc232336247)

[Table 64. Study 2 Inferential Statistics for Traits by Condition and Race 78](#_Toc232336248)

[Table 65. Study 2 Inferential Statistics for Feeling Thermometer by Condition and Race 79](#_Toc232336249)

[Table 66. Study 2 Inferential Statistics for Political Abilities by Condition and Race 80](#_Toc232336250)

[Table 67. Study 2 Inferential Statistics for Superior/Inferior by Condition and Race 81](#_Toc232336251)

[Table 68. Study 2 Inferential Statistics for American/Foreign by Condition and Race 82](#_Toc232336252)

# Supplemental Study 1 Materials

Study 1 contained the following deviations from preregistration (<https://osf.io/xn23q/overview?view_only=8cfaf19a7a7d4e49bbde922872b21748>),

1. We intended to collect data from 600 participants but stopped data collection at 598 participants on 9/10/24 because the presidential debate was occurring on that evening. We wanted to ensure that all data were collected before the debate. Our preregistered target sample size based on an a priori power analysis was 540 so we had exceeded that target when we stopped data collection. Our sample for analysis after data exclusion of 592 also exceeded our preregistered target sample size.
2. Although not a preregistered exclusion criterion, we omitted data from 1 participant who reported not living in the United States as our inclusion criteria of U.S. population was intended to recruit only from those living in the U.S.
3. We included traits related to competence, communality, and dominance because of their associations with different gender and racial groups. Our preregistered analyses focused on separate composite competence, communality, and dominance scores. However, preliminary analyses showed by lower internal reliability for the dominance scale and similar patterns of results across the three dimensions when they were considered separately. This led us to examine whether participants were treating the traits as 3 separate dimensions. Exploratory and confirmatory factor analyses revealed that a two-factor valence-based solution based on the favorability of items (e.g., *friendly* vs *bossy*) with *dominant* and *assertive* omitted due to moderately high loadings on both factors had the best global fit based on RMSEA, CFI, and SRMR statistics. The main text thus reports analyses on the positive and negative factors (with dominant and assertive omitted) but analyses on the separate competence, communality, and dominance factors are reported here and support the same conclusions as those in the main text in terms of evaluations being affected by priming race.
4. We specified 8 contrasts in the preregistration to test all hypotheses. We subsequently realized we could test the hypotheses more efficiently with a smaller set of contrasts. The new contrasts also had the benefit of corresponding to tests of a main effect of specifying Harris’s race, a main effect of specifying Harris’s gender, and their interaction (with an additional contrast to compare the other condition to all Harris conditions). For efficiency and conceptual ease, we report the smaller set of contrasts in the main text. The preregistered contrasts can be found in Tables 13-20 in this document.

The full stimulus materials describing either Kamala Harris or the unnamed other candidate as shown in Table 1 and the ancillary measures not reported in the main text are in Table 2.

## Table 1. Study 1 Priming Paragraph Text

| Condition | Text |
| --- | --- |
| Race | Kamala Harris is the Vice President of the United States, making history as the first Black and South Asian American to hold the office. Her groundbreaking achievement represents a significant step forward for racial equality in U.S. leadership. Prior to this role, Harris was a U.S. Senator from California and the state's Attorney General. She has consistently advocated for civil rights, criminal justice reform, and healthcare access. Harris’s leadership reflects the increasing racial diversity in American political representation. |
| Gender | Kamala Harris is the Vice President of the United States, making history as the first woman to hold the office. Her groundbreaking achievement represents a significant step forward for gender equality in U.S. leadership. Prior to this role, Harris was a U.S. Senator from California and the state's Attorney General. She has consistently advocated for civil rights, criminal justice reform, and health care access. Harris’s leadership reflects the increasing gender diversity in American political representation. |
| Race and Gender | Kamala Harris is the Vice President of the United States, making history as the first woman and the first Black and South Asian American to hold the office. Her groundbreaking achievement represents a significant step  forward for gender and racial equality in U.S. leadership. Prior to this role, Harris was a U.S. Senator from California and the state's Attorney General. She has consistently advocated for civil rights, criminal justice reform, and healthcare access. Harris’s leadership reflects the increasing gender and racial diversity in American political representation. |
| No Mention of Race or Gender | Kamala Harris is the Vice President of the United States, bringing extensive experience in public service to the role. Prior to this role, Harris was a U.S. Senator from California and the state's Attorney General. She has consistently advocated for civil rights, criminal justice reform, and healthcare access. Harris’s leadership reflects a significant milestone in American political history, reflecting her commitment to justice and equality. |
| Other candidate | A current politician who is up for reelection brings extensive experience in public service to the role. This politician is a U.S. Senator from New York and the state's Attorney General. They have consistently advocated for civil rights, criminal justice reform, and healthcare access. Their leadership reflects a significant milestone in American political history, reflecting their commitment to justice and equality. |

## Table 2. Study 1 Additional Measures

| Measure | Item | Response Options |
| --- | --- | --- |
| Registration | Are you registered to vote? | *Yes, No* |
| Party | What political party are you registered to vote with, if any? | *1 = Democratic Party, 2 = Republican Party, 3 = None, 4 = Independent, 5 = Other* |
| Voter Registration Intention | Do you intend to register to vote before the November 5th, 2024 elections or do you not intend to register | *Yes, intend to register*  *No, do not intend to register* |
| Registered Voter State | In what state are you registered to vote now? | *Select a State* |
| Voting in the Presidential Primary | In the presidential primary or caucus, who did you vote for? | *1 = Joe Biden, 2 = Another Democrat, 3 = Donald Trump, 4 = Another Republican, 5 = Someone else who is not a Republican or a Democrat, 6 = Did not vote* |
| Harris’s Performance | Overall, how would you rate Kamala Harris's performance as a political leader | *1 – Very Negative to*  *5 – Very Positive* |
| Harris DEI Hire | To what degree do you consider Kamala Harris as a DEI (Diversity, Equity and Inclusion) Hire? | *1 – strongly disagree to 7 – strongly agree* |
| DEI Hire Reasoning | Can you tell us more about why you chose selected choices? | Open ended response |
| Harris DEI Reasoning | Why do you think Kamala Harris is a DEI Hire? Because of... (Check all that Apply) | *1 = Her Gender, 2 = Her Black Heritage, 3 = Her South Asian heritage* |
| DEI Hire Definition | Under what circumstances do you consider someone a DEI Hire? | Open ended response |
| Perceptions of Harris (Black) | To what degree do you think Kamala Harris is Black? | *1 = Not at all - 5 = A great deal* |
| Perceptions of Harris (South Asian) | To what degree do you think Kamala Harris is South Asian? | *1 = Not at all - 5 = A great deal* |
| Harris’s perceptions of Her Black Heritage | To what degree do you think Kamala Harris views herself as Black? | *1 = Not at all - 5 = A great deal* |
| Harris’s perceptions of Her South Asian Heritage | To what degree do you think Kamala Harris views herself as South Asian? | *1 = Not at all - 5 = A great deal* |
| Reasons to Vote for Kamala | What reasons might make you want to vote for Kamala Harris? | Open ended response |
| Reasons to Vote Against Kamala | What reasons might make you want to NOT vote for Kamala Harris? | Open ended response |
| Harris’s Overall Performance | Overall, how would you rate Kamala Harris's performance as a political leader? | 1 = *Very negative –* 5 *= Very positive* |
| Harris’s Race | What do you think Kamala Harris's race is? | Open ended response |

# Supplemental Study 1 Analyses

Data and R code are available at (<https://osf.io/aq26w/?view_only=2a8bc42887204afe87981eda81ab464b>).

Results for the Exploratory Factor Analysis of the trait items are in Table 3 and results for the Confirmatory Factor Analysis are in Table 4.

Tables 5-9 present the descriptive and full inferential statistics for the measures of positive traits, negative traits, feeling thermometer, and political abilities that are discussed in the main text.

Tables 10-12 report analyses on separate composite scores for competence, communality, and dominance using the contrasts reported in the main text. We also report analyses on the separate composite scores for competence, communality, and dominance using the contrasts in the preregistration. The preregistered contrast codes are in Table 13 and the results using those codes are in Tables 14-16.

Tables 17-20 report analyses on the measures presented in the main text using the preregistered contrasts (composite positive and negative traits, feeling thermometer, and political ability).

Tables 21-25 report analyses on additional measures not reported in the main text. This includes perceptions of Harris as Black and South Asian. We measured both the degree to which participants agreed that Harris was Black and South Asian, and also the degree to which participants agreed that Harris viewed herself as Black and South Asian. The only effect across all measures was a simple effect of political orientation such that as conservatism increased, participants increasingly disagreed.

For exploratory purposes, we replicated the analyses reported in the main text with participant gender and then with participant race as moderators. Gender was coded Man = -1/2, Not Man = 1/2, and race was coded Person of Color = -1/2, Not Person of Color = 1/2. As seen in Tables 25-33, neither participant gender nor race moderated any of the effects reported in the main text.

## Table 3. Study 1 Exploratory Factor Analysis Loadings for Two-Factor Extraction

| **Trait** | **Positive** | **Negative** |
| --- | --- | --- |
| Ambitious | 0.522 |  |
| Competent | 0.833 |  |
| Confident | 0.801 |  |
| Intelligent | 0.825 |  |
| Bossy |  | 0.715 |
| Controlling |  | 0.756 |
| Demanding |  | 0.748 |
| Dominant | 0.616 | 0.482 |
| Assertive | 0.755 | 0.327 |
| Powerful | 0.802 |  |
| Leader | 0.871 |  |
| Friendly | 0.670 |  |
| Kind | 0.714 |  |
| Likeable | 0.743 |  |
| Sensitive to the needs of Other | 0.743 |  |
| Warm | 0.669 |  |

*Note.* Factor loadings less than .3 are not shown.

## Table 4. Study 1 Confirmatory Factor Analysis Fit Statistics

| Model statistics | Dominance, Competence, and Communality | Agency and Communality | Positive and Negative | Positive and Negative Dropped* |
| --- | --- | --- | --- | --- |
| *Χ^2^* | 975.233 | 978.069 | 704.557 | 250.186 |
| *^X2^_df_* | 101 | 103 | 103 | 76 |
| *X^2^ p* | <.001 | <.001 | <.001 | <.001 |
| CFI | 0.949 | 0.949 | 0.965 | 0.989 |
| RMSEA | 0.121 | 0.120 | 0.099 | 0.062 |
| lower | 0.114 | 0.113 | 0.093 | 0.054 |
| upper | 0.128 | 0.127 | 0.107 | 0.071 |
| SRMR | 0.125 | 0.125 | 0.108 | 0.071 |

*Note.* Positive and Negative Dropped* is the model with Assertive and Dominant not included in the analysis. The two-factor positive and negative model with Dominant and Assertive dropped had the best global fit based on RMSEA, CFI, and SRMR statistics. This is the factor structure reported in the manuscript.

## Table 5. Study 1 Descriptive Statistics for Outcomes by Condition

| Condition | Positive Traits  *M* (*SD*) | Negative Traits  *M* (*SD*) | Political Abilities  *M* (*SD*) | Feeling Thermometer  *M* (*SD*) |
| --- | --- | --- | --- | --- |
| Gender | 3.57 (1.02) | 2.93 (0.98) | 3.52 (1.42) | 55.87 (34.95) |
| Race | 3.26 (1.12) | 3.18 (1.00) | 3.26 (1.44) | 51.03 (34.65) |
| Both | 3.37 (1.12) | 3.20 (0.97) | 3.32 (1.46) | 51.35 (35.84) |
| None | 3.48 (1.05) | 3.03 (1.03) | 3.48 (1.44) | 57.78 (34.86) |
| Other | 3.72 (0.71) | 3.09 (0.93) | 3.98 (0.86) | 60.06 (25.09) |

*Note.*  Values represent raw means (*M*) and standard deviations (*SD*) for each outcome across the five experimental conditions.

Table 6. Study 1 Inferential Statistics for Positive Ratings by Condition and Political Orientation

| Effect | Estimate | *t* | *SE* | 95% CI | | η²ₚ | *p* |
| --- | --- | --- | --- | --- | --- | --- | --- |
|  |  |  |  | *LL* | *UL* |  |  |
| Fixed effects |  |  |  |  |  |  |  |
| Intercept | 3.47 | 102.73 | 0.03 | 3.41 | 3.54 | 0.948 | <.001^***^ |
| Race vs No Race | -0.10 | -2.65 | 0.04 | -0.17 | -0.03 | 0.012 | 0.008^**^ |
| Gender vs No Gender | 0.03 | 0.68 | 0.04 | -0.05 | 0.10 | 0.001 | 0.498 |
| Gender X Race | 0.02 | 0.42 | 0.04 | -0.06 | 0.09 | <.001 | 0.676 |
| Harris vs. Other | 0.07 | 3.80 | 0.02 | 0.03 | 0.10 | 0.024 | <.001^***^ |
| Political Orientation (PO) | -0.32 | -16.65 | 0.02 | -0.36 | -0.28 | 0.323 | <.001^***^ |
| Race vs No Race X PO | -0.03 | -1.36 | 0.02 | -0.07 | 0.01 | 0.003 | 0.175 |
| Gender vs No Gender X PO | 0.003 | 0.14 | 0.02 | -0.04 | 0.04 | <.001 | 0.889 |
| Gender X Race X PO | -0.01 | -0.33 | 0.02 | -0.05 | 0.03 | <.001 | 0.744 |
| Harris vs. Other X PO | 0.05 | 4.93 | 0.01 | 0.03 | 0.07 | 0.040 | <.001^***^ |

*Note.*  CI = confidence interval; *LL* = lower limit; *UL* = upper limit. * *p* < .05, ** *p* < .01, *** *p* < .001.

## Table 7. Study 1 Inferential Statistics for Negative Ratings by Condition and Political Orientation

| Effect | Estimate | *t* | *SE* | 95% CI | | η²ₚ | *p* |
| --- | --- | --- | --- | --- | --- | --- | --- |
|  |  |  |  | *LL* | *UL* |  |  |
| Fixed effects |  |  |  |  |  |  |  |
| Intercept | 3.09 | 80.93 | 0.04 | 3.01 | 3.16 | 0.918 | <.001^***^ |
| Race vs No Race | 0.10 | 2.31 | 0.04 | 0.02 | 0.18 | 0.009 | 0.021^**^ |
| Gender vs No Gender | -0.003 | -0.07 | 0.04 | -0.09 | 0.08 | <.001 | 0.945 |
| Gender X Race | 0.02 | 0.51 | 0.04 | -0.06 | 0.11 | <.001 | 0.613 |
| Harris vs. Other | -0.01 | -0.25 | 0.02 | -0.04 | 0.03 | <.001 | 0.806 |
| Political Orientation (PO) | 0.19 | 8.68 | 0.02 | 0.15 | 0.23 | 0.115 | <.001^***^ |
| Race vs No Race X PO | -0.01 | -0.30 | 0.02 | -0.05 | 0.04 | <.001 | 0.766 |
| Gender vs No Gender X PO | 0.01 | 0.35 | 0.02 | -0.04 | 0.06 | <.001 | 0.728 |
| Gender X Race X PO | 0.002 | 0.09 | 0.02 | -0.04 | 0.05 | <.001 | 0.927 |
| Harris vs. Other X PO | -0.01 | -0.85 | 0.01 | -0.03 | 0.01 | 0.001 | 0.399 |

*Note.*  CI = confidence interval; *LL* = lower limit; *UL* = upper limit. * *p* < .05, ** *p* < .01, *** *p* < .001.

## Table 8. Study 1 Inferential Statistics for Feeling Thermometer by Condition and Political Orientation

| Effect | Estimate | *t* | *SE* | 95% CI | | η²ₚ | *p* |
| --- | --- | --- | --- | --- | --- | --- | --- |
|  |  |  |  | *LL* | *UL* |  |  |
| Fixed effects |  |  |  |  |  |  |  |
| Intercept | 55.02 | 51.64 | 1.07 | 52.93 | 57.12 | 0.821 | <.001^***^ |
| Race vs No Race | -2.57 | -2.17 | 1.19 | -4.91 | -0.24 | 0.008 | 0.031^*^ |
| Gender vs No Gender | -1.34 | -1.13 | 1.19 | -3.67 | 0.99 | 0.002 | 0.259 |
| Gender X Race | 0.99 | 0.84 | 1.19 | -1.34 | 3.33 | 0.001 | 0.403 |
| Harris vs. Other | 1.46 | 2.72 | 0.54 | 0.41 | 2.52 | 0.013 | 0.007^**^ |
| Political Orientation (PO) | -11.38 | -18.91 | 0.60 | -12.56 | -10.20 | 0.381 | <.001^***^ |
| Race vs No Race X PO | -0.57 | -0.87 | 0.66 | -1.87 | 0.73 | 0.001 | 0.387 |
| Gender vs No Gender X PO | -0.37 | -0.55 | 0.66 | -1.67 | 0.93 | 0.001 | 0.580 |
| Gender X Race X PO | -0.02 | -0.04 | 0.66 | -1.32 | 1.28 | <.001 | 0.971 |
| Harris vs. Other X PO | 1.44 | 4.58 | 0.314 | 0.82 | 2.06 | 0.035 | <.001^***^ |

*Note.*  CI = confidence interval; *LL* = lower limit; *UL* = upper limit. * *p* < .05, ** *p* < .01, *** *p* < .001.

## Table 9. Study 1 Inferential Statistics for Political Abilities Ratings by Condition and Political Orientation

| Effect | Estimate | *t* | *SE* | 95% CI | | η²ₚ | *p* |
| --- | --- | --- | --- | --- | --- | --- | --- |
|  |  |  |  | *LL* | *UL* |  |  |
| Fixed effects |  |  |  |  |  |  |  |
| Intercept | 3.51 | 79.26 | 0.04 | 3.42 | 3.59 | 0.915 | <.001^***^ |
| Race vs No Race | -0.10 | -1.93 | 0.05 | -0.19 | 0.002 | 0.006 | 0.054 |
| Gender vs No Gender | -0.01 | -0.17 | 0.05 | -0.11 | 0.09 | <.001 | 0.862 |
| Gender X Race | 0.02 | 0.39 | 0.05 | -0.08 | 0.12 | <.001 | 0.695 |
| Harris vs. Other | 0.12 | 5.54 | 0.02 | 0.08 | 0.17 | 0.050 | <.001^***^ |
| Political Orientation (PO) | -0.43 | -17.22 | 0.03 | -0.48 | -0.38 | 0.338 | <.001^***^ |
| Race vs No Race X PO | -0.02 | -0.89 | 0.03 | -0.08 | 0.03 | 0.001 | 0.376 |
| Gender vs No Gender X PO | 0.02 | 0.69 | 0.03 | -0.04 | 0.07 | 0.001 | 0.491 |
| Gender X Race X PO | 0.003 | 0.10 | 0.03 | -0.05 | 0.06 | <.001 | 0.919 |
| Harris vs. Other X PO | 0.07 | 5.22 | 0.01 | 0.04 | 0.09 | 0.045 | <.001^***^ |

*Note.*  CI = confidence interval; *LL* = lower limit; *UL* = upper limit.* *p* < .05, ** *p* < .01, *** *p* < .001.

## Table 10. Study 1 Inferential Statistics on Competence Traits by Condition and Political Orientation

| Effect | Estimate | *t* | *SE* | 95% CI | | η²ₚ | *p* |
| --- | --- | --- | --- | --- | --- | --- | --- |
|  |  |  |  | *LL* | *UL* |  |  |
| Fixed effects |  |  |  |  |  |  |  |
| Intercept | 3.83 | 112.54 | 0.03 | 3.76 | 3.89 | 0.956 | <.001*** |
| Race vs No Race | -0.08 | -2.09 | 0.04 | -0.15 | -0.01 | 0.007 | 0.037* |
| Gender vs No Gender | 0.02 | 0.43 | 0.04 | -0.06 | 0.09 | <.001 | 0.664 |
| Gender X Race | 0.01 | 0.15 | 0.04 | -0.07 | 0.80 | <.001 | 0.882 |
| Harris vs. Other | 0.06 | 3.43 | 0.02 | 0.03 | 0.09 | 0.020 | 0.001** |
| Political Orientation (PO) | -0.29 | -15.27 | 0.02 | -0.33 | -0.26 | 0.286 | <.001*** |
| Race vs No Race X PO | -0.04 | -1.77 | 0.02 | -0.08 | 0.004 | 0.005 | 0.077 |
| Gender vs No Gender X PO | -0.01 | -0.28 | 0.02 | -0.05 | 0.04 | <.001 | 0.782 |
| Gender X Race X PO | 0.01 | 0.41 | 0.02 | -0.03 | 0.05 | <.001 | 0.685 |
| Harris vs. Other X PO | 0.05 | 4.51 | 0.01 | 0.03 | 0.07 | 0.034 | <.001*** |

*Note.*  CI = confidence interval; *LL* = lower limit; *UL* = upper limit.* *p* < .05, ** *p* < .01, *** *p* < .001.

## Table 11. Study 1 Inferential Statistics on Communality by Condition and Political Orientation

| Effect | Estimate | *t* | *SE* | 95% CI | | η²ₚ | *p* |
| --- | --- | --- | --- | --- | --- | --- | --- |
|  |  |  |  | *LL* | *UL* |  |  |
| Fixed effects |  |  |  |  |  |  |  |
| Intercept | 3.20 | 81.82 | 0.04 | 3.12 | 3.28 | 0.920 | <.001*** |
| Race vs No Race | -0.12 | -2.71 | 0.04 | -0.20 | -0.03 | 0.012 | 0.007** |
| Gender vs No Gender | 0.06 | 1.34 | 0.04 | -0.03 | 0.14 | 0.003 | 0.180 |
| Gender X Race | 0.03 | 0.66 | 0.04 | -0.06 | 0.11 | 0.001 | 0.511 |
| Harris vs. Other | 0.05 | 2.68 | 0.02 | 0.01 | 0.09 | 0.012 | 0.008** |
| Political Orientation (PO) | -0.32 | -14.45 | 0.02 | -0.36 | -0.28 | 0.264 | <.001*** |
| Race vs No Race X PO | -0.30 | -1.05 | 0.02 | -0.07 | 0.02 | 0.002 | 0.292 |
| Gender vs No Gender X PO | 0.01 | 0.60 | 0.02 | -0.03 | 0.06 | 0.001 | 0.552 |
| Gender X Race X PO | -0.01 | -0.50 | 0.02 | 0.06 | 0.04 | <.001 | 0.616 |
| Harris vs. Other X PO | 0.05 | 4.39 | 0.01 | 0.03 | 0.07 | 0.032 | <.001*** |

*Note.*  CI = confidence interval; *LL* = lower limit; *UL* = upper limit.* *p* < .05, ** *p* < .01, *** *p* < .001.

## Table 12: Study 1 Inferential Statistics on Dominance Traits by Condition and Political Orientation

| Effect | Estimate | *t* | *SE* | 95% CI | | η²ₚ | *p* |
| --- | --- | --- | --- | --- | --- | --- | --- |
|  |  |  |  | *LL* | *UL* |  |  |
| Fixed effects |  |  |  |  |  |  |  |
| Intercept | 3.31 | 125.99 | 0.03 | 3.26 | 3.36 | 0.965 | <.001*** |
| Race vs No Race | -0.004 | -0.12 | 0.03 | -0.06 | 0.05 | <.001 | 0.904 |
| Gender vs No Gender | -0.02 | -0.63 | 0.03 | -0.08 | 0.04 | 0.001 | 0.531 |
| Gender X Race | 0.001 | 0.05 | 0.03 | -0.06 | 0.06 | <.001 | 0.964 |
| Harris vs. Other | 0.05 | 3.61 | 0.01 | 0.02 | 0.07 | 0.022 | <.001*** |
| Political Orientation (PO) | -0.06 | -4.03 | 0.02 | -0.09 | -0.03 | 0.027 | <.001*** |
| Race vs No Race X PO | -0.02 | -1.14 | 0.02 | -0.05 | 0.01 | 0.002 | 0.254 |
| Gender vs No Gender X PO | -0.01 | -0.76 | 0.02 | -0.05 | 0.02 | 0.001 | 0.446 |
| Gender X Race X PO | -0.004 | -0.24 | 0.02 | -0.04 | 0.03 | <.001 | 0.811 |
| Harris vs. Other X PO | 0.02 | 2.69 | 0.01 | 0.01 | 0.04 | 0.012 | 0.007* |

*Note.*  CI = confidence interval; *LL* = lower limit; *UL* = upper limit.* *p* < .05, ** *p* < .01, *** *p* < .001.

## Table 13. Study 1 Preregistered Contrast Codes

|  | Condition | | | | |
| --- | --- | --- | --- | --- | --- |
| Contrast | Race | Gender | Both | None | Other |
| Both + Race vs. Other | -1 | 0 | -1 | 0 | +2 |
| Both + Race vs. None | -1 | 0 | -1 | +2 | 0 |
| Both vs. Race | -1 | 0 | +1 | 0 | 0 |
| Both + Gender vs. Other | 0 | -1 | -1 | 0 | +2 |
| Both + Gender vs. None | 0 | -1 | -1 | +2 | 0 |
| Both vs. Gender | 0 | -1 | +1 | 0 | 0 |
| Both vs. None | 0 | 0 | 0 | 1 | 0 |
| Both vs. Other | 0 | 0 | 0 | 0 | 1 |

## Table 14. Study 1 Inferential Statistics for Preregistered Contrasts on Competence Ratings by Condition and Political Orientation

| Effect | Estimate | *t* | *SE* | 95% CI | | η²ₚ | *p* |
| --- | --- | --- | --- | --- | --- | --- | --- |
|  |  |  |  | *LL* | *UL* |  |  |
| Fixed effects |  |  |  |  |  |  |  |
| Intercept | 3.83 | 112.54 | 0.03 | 3.76 | 3.89 | 0.956 | <.001*** |
| Both + Race vs. Other | 0.12 | 3.98 | 0.03 | 0.06 | 0.19 | 0.995 | <.001*** |
| Both + Race vs. None | 0.05 | 1.58 | 0.03 | -0.01 | 0.11 | 0.004 | 0.116 |
| Both vs. Race | 0.02 | 0.41 | 0.05 | -0.08 | 0.13 | <.001 | 0.680 |
| Both + Gender vs. Other | 0.09 | 2.97 | 0.03 | 0.03 | 0.15 | 0.015 | 0.003** |
| Both + Gender vs. None | 0.02 | 0.56 | 0.03 | -0.04 | 0.08 | 0.001 | 0.579 |
| Both vs. Gender | -0.07 | -1.39 | 0.05 | -0.18 | 0.03 | 0.003 | 0.166 |
| Both vs. Other | 0.35 | 3.28 | 0.11 | 0.14 | 0.56 | 0.018 | 0.001** |
| Both vs. None | 0.13 | 1.17 | 1.11 | -0.09 | 3.86 | 0.002 | <.001*** |
| Political Orientation (PO) | -0.29 | -15.27 | 0.02 | -0.33 | -0.26 | 0.286 | <.001*** |
| Both + Race vs. Other X PO | 0.09 | 4.87 | 0.02 | 0.05 | 0.012 | 0.039 | <.001*** |
| Both + Race vs. None X PO | 0.03 | 1.75 | 0.02 | -0.004 | 0.06 | 0.005 | 0.081 |
| Both vs. Race X PO | 0.003 | 0.09 | 0.03 | -0.06 | 0.06 | <.001 | 0.927 |
| Both + Gender vs. Other X PO | 0.08 | 4.28 | 0.02 | 0.04 | 0.11 | 0.030 | <.001*** |
| Both + Gender vs. None X PO | 0.02 | 1.13 | 0.02 | -0.01 | 0.05 | 0.002 | 0.259 |
| Both vs. Gender X PO | -0.03 | -0.97 | 0.03 | -0.09 | 0.03 | 0.002 | 0.331 |
| Both vs. Other X PO | 0.26 | 4.31 | 0.06 | 0.14 | 0.38 | 0.031 | <.001*** |
| Both vs. None X PO | 0.09 | 1.50 | 0.06 | -0.03 | 0.20 | 0.004 | 0.13 |

*Note.*  CI = confidence interval; *LL* = lower limit; *UL* = upper limit. * *p* < .05, ** *p* < .01, *** *p* < .001.

## Table 15. Study 1 Inferential Statistics for Preregistered Contrasts on Communality Ratings by Condition and Political Orientation

| Effect | Estimate | *t* | *SE* | 95% CI | | η²ₚ | *p* |
| --- | --- | --- | --- | --- | --- | --- | --- |
|  |  |  |  | *LL* | *UL* |  |  |
| Fixed effects |  |  |  |  |  |  |  |
| Intercept | 3.20 | 81.82 | 0.04 | 3.12 | 3.28 | 0.920 | <.001*** |
| Both + Race vs. Other | 0.13 | 3.55 | 0.04 | 0.06 | 0.20 | 0.021 | <.001*** |
| Both + Race vs. None | 0.07 | 1.92 | 0.04 | -0.002 | 0.14 | 0.006 | 0.056 |
| Both vs. Race | 0.09 | 1.42 | 0.06 | -0.03 | 0.21 | 0.003 | 0.157 |
| Both + Gender vs. Other | 0.07 | 1.92 | 0.04 | -0.002 | 0.14 | 0.006 | 0.056 |
| Both + Gender vs. None | 0.01 | 0.28 | 0.04 | -0.06 | 0.08 | <.001 | 0.782 |
| Both vs. Gender | -0.09 | -1.47 | 0.06 | -0.21 | 0.03 | 0.004 | 0.143 |
| Both vs. Other | 0.30 | 2.40 | 0.12 | 0.05 | 0.54 | 0.010 | 0.0117 |
| Both vs. None | 0.12 | 0.97 | 0.12 | -0.12 | 0.36 | 0.002 | 0.334 |
| Political Orientation (PO) | -0.32 | -14.45 | 0.02 | -0.36 | -0.28 | 0.264 | <.001*** |
| Both + Race vs. Other X PO | 0.09 | 4.47 | 0.02 | 0.05 | 0.13 | 0.033 | <.001 |
| Both + Race vs. None X PO | 0.01 | 0.42 | 0.02 | -0.03 | 0.05 | <.001 | 0.675 |
| Both vs. Race X PO | 0.002 | 0.07 | 0.03 | -0.06 | 0.07 | <.001 | 0.946 |
| Both + Gender vs. Other X PO | 0.08 | 3.82 | 0.02 | 0.04 | 0.12 | 0.024 | <.001*** |
| Both + Gender vs. None X PO | -0.01 | -0.26 | 0.02 | -0.04 | 0.03 | <.001 | 0.792 |
| Both vs. Gender X PO | -0.04 | -1.11 | 0.03 | -0.11 | 0.03 | 0.002 | 0.269 |
| Both vs. Other X PO | 0.28 | 3.97 | 0.07 | 0.14 | 0.41 | 0.026 | <.001*** |
| Both vs. None X PO | 0.02 | 0.34 | 0.07 | -0.11 | 0.15 | <.001 | 0.737 |

*Note.*  CI = confidence interval; *LL* = lower limit; *UL* = upper limit. * *p* < .05, ** *p* < .01, *** *p* < .001.

## Table 16. Study 1 Inferential Statistics for Preregistered Contrasts on Dominance Ratings by Condition and Political Orientation

| Effect | Estimate | *t* | *SE* | 95% CI | | η²ₚ | *p* |
| --- | --- | --- | --- | --- | --- | --- | --- |
|  |  |  |  | *LL* | *UL* |  |  |
| Fixed effects |  |  |  |  |  |  |  |
| Intercept | 3.31 | 125.99 | 0.03 | 3.26 | 3.36 | 0.965 | <.001*** |
| Both + Race vs. Other | 0.08 | 3.36 | 0.02 | 0.03 | 0.13 | 0.019 | 0.001** |
| Both + Race vs. None | 0.01 | 0.037 | 0.02 | -0.04 | 0.06 | <.001 | 0.711 |
| Both vs. Race | -0.02 | -0.41 | 0.04 | -0.10 | 0.06 | <.001 | 0.680 |
| Both + Gender vs. Other | 0.09 | 3.57 | 0.02 | 0.04 | 0.13 | 0.021 | <.001*** |
| Both + Gender vs. None | 0.01 | 0.58 | 0.02 | -0.03 | 0.06 | 0.001 | 0.564 |
| Both vs. Gender | -0.002 | -0.05 | 0.04 | -0.08 | 0.08 | <.001 | 0.56 |
| Both vs. Other | 0.26 | 3.14 | 0.08 | 0.10 | 0.42 | 0.017 | 0.002** |
| Both vs. None | 0.04 | 0.53 | 0.08 | -0.12 | 0.21 | <.001 | 0.60 |
| Political Orientation (PO) | -0.06 | -4.03 | 0.02 | -0.09 | -0.03 | 0.027 | <.001*** |
| Both + Race vs. Other X PO | 0.04 | 2.93 | 0.01 | 0.01 | 0.07 | 0.015 | 0.004** |
| Both + Race vs. None X PO | 0.02 | 1.16 | 0.01 | -0.01 | 0.04 | 0.002 | 0.245 |
| Both vs. Race X PO | -0.02 | -0.72 | 0.02 | -0.06 | 0.03 | 0.001 | 0.474 |
| Both + Gender vs. Other X PO | 0.04 | 2.78 | 0.01 | 0.01 | 0.07 | 0.013 | 0.006** |
| Both + Gender vs. None X PO | 0.01 | 1.00 | 0.01 | -0.01 | 0.04 | 0.002 | 0.316 |
| Both vs. Gender X PO | -0.02 | -0.98 | 0.02 | -0.07 | 0.02 | 0.002 | 0.326 |
| Both vs. Other X PO | 0.14 | 2.97 | 0.05 | 0.05 | 0.23 | 0.015 | 0.003 |
| Both vs. None X PO | 0.06 | 1.39 | 0.05 | -0.03 | 0.15 | 0.003 | 0.164 |

*Note.*  CI = confidence interval; *LL* = lower limit; *UL* = upper limit. * *p* < .05, ** *p* < .01, *** *p* < .001.

## Table 17. Study 1 Inferential Statistics for Preregistered Contrasts on Positive Ratings by Condition and Political Orientation

| Effect | Estimate | *t* | *SE* | 95% CI | | η²ₚ | *p* |
| --- | --- | --- | --- | --- | --- | --- | --- |
|  |  |  |  | *LL* | *UL* |  |  |
| Fixed effects |  |  |  |  |  |  |  |
| Intercept | 3.47 | 102.73 | 0.03 | 3.41 | 3.54 | 0.948 | <.001*** |
| Both + Race vs. Other | 0.14 | 4.55 | 0.03 | 0.08 | 0.20 | 0.034 | <.001*** |
| Both + Race vs. None | 0.06 | 2.04 | 0.03 | 0.002 | 0.12 | 0.007 | 0.041 |
| Both vs. Race | 0.04 | 0.78 | 0.05 | -0.06 | 0.15 | 0.001 | 0.438 |
| Both + Gender vs. Other | 0.10 | 3.21 | 0.03 | 0.04 | 0.16 | 0.017 | 0.001** |
| Both + Gender vs. None | 0.02 | 0.70 | 0.03 | -0.04 | 0.08 | 0.001 | 0.487 |
| Both vs. Gender | -0.08 | -1.60 | 0.05 | -0.19 | 0.02 | 0.004 | 0.111 |
| Both vs. Other | 0.38 | 3.59 | 0.11 | 0.17 | 0.59 | 0.003 | <.001*** |
| Both vs. None | 0.15 | 1.40 | 0.11 | -0.06 | 0.36 | 0.003 | 0.163 |
| Political Orientation (PO) | -0.32 | -16.65 | 0.02 | -0.36 | -0.28 | 0.323 | <.001*** |
| Both + Race vs. Other X PO | 0.09 | 5.09 | 0.02 | 0.06 | 0.13 | 0.043 | <.001*** |
| Both + Race vs. None X PO | 0.02 | 0.93 | 0.02 | -0.02 | 0.05 | 0.001 | 0.352 |
| Both vs. Race X PO | -0.004 | -0.13 | 0.03 | -0.06 | 0.05 | <.001 | 0.893 |
| Both + Gender vs. Other X PO | 0.08 | 4.49 | 0.02 | 0.05 | 0.01 | 0.034 | 0.415 |
| Both + Gender vs. None X PO | 0.01 | 0.31 | 0.02 | -0.03 | 0.04 | <.001 | 0.756 |
| Both vs. Gender X PO | -0.04 | -1.20 | 0.03 | -0.09 | 0.02 | 0.002 | 0.232 |
| Both vs. Other X PO | 0.28 | 4.62 | 0.06 | 0.16 | 0.40 | 0.035 | <.001*** |
| Both vs. None X PO | 0.05 | 089 | 0.06 | -0.06 | 0.16 | 0.001 | 0.373 |

*Note.*  CI = confidence interval; *LL* = lower limit; *UL* = upper limit. * *p* < .05, ** *p* < .01, *** *p* < .001.

## Table 18. Study 1 Inferential Statistics for Preregistered Contrasts on Negative Ratings by Condition and Political Orientation

| Effect | Estimate | *t* | *SE* | 95% CI | | η²ₚ | *p* |
| --- | --- | --- | --- | --- | --- | --- | --- |
|  |  |  |  | *LL* | *UL* |  |  |
| Fixed effects |  |  |  |  |  |  |  |
| Intercept | 3.09 | 80.93 | 0.04 | 3.01 | 3.16 | 0.918 | <.001*** |
| Both + Race vs. Other | -0.04 | -1.16 | 0.04 | -0.11 | 0.03 | 0.002 | 0.246 |
| Both + Race vs. None | -0.06 | -1.64 | 0.04 | -0.13 | 0.01 | 0.005 | 0.101 |
| Both vs. Race | 0.02 | 0.31 | 0.06 | -0.10 | 0.14 | <.001 | 0.757 |
| Both + Gender vs. Other | -0.01 | -0.20 | 0.04 | -0.08 | 0.06 | <.001 | 0.844 |
| Both + Gender vs. None | -0.02 | -0.68 | 0.04 | -0.09 | 0.05 | 0.001 | 0.497 |
| Both vs. Gender | 0.12 | 2.02 | 0.06 | 0.003 | 0.24 | 0.007 | 0.044 |
| Both vs. Other | -0.14 | -1.17 | 0.12 | -0.38 | 0.10 | 0.002 | 0.242 |
| Both vs. None | -0.19 | -1.59 | 0.12 | -0.43 | 0.05 | 0.004 | 0.112 |
| Political Orientation (PO) | 0.19 | 8.68 | 0.02 | 0.15 | 0.23 | 0.115 | <.001*** |
| Both + Race vs. Other X PO | -0.01 | -0.67 | 0.02 | -0.05 | 0.03 | 0.001 | 0.506 |
| Both + Race vs. None X PO | 0.003 | 0.14 | 0.02 | -0.04 | 0.04 | <.001 | 0.888 |
| Both vs. Race X PO | 0.01 | 0.31 | 0.03 | -0.06 | 0.08 | 0.001 | 0.754 |
| Both + Gender vs. Other X PO | -0.02 | -0.92 | 0.02 | -0.06 | 0.02 | 0.001 | 0.360 |
| Both + Gender vs. None X PO | -0.002 | -0.13 | 0.02 | -0.04 | 0.04 | <.001 | 0.899 |
| Both vs. Gender X PO | -0.01 | -0.15 | 0.03 | -0.07 | 0.06 | <.001 | 0.883 |
| Both vs. Other X PO | -0.05 | -0.75 | 0.07 | -0.18 | 0.08 | 0.001 | 0.455 |
| Both vs. None X PO | -0.002 | -0.04 | 0.07 | -0.13 | 0.13 | <.001 | 0.971 |

*Note.*  CI = confidence interval; *LL* = lower limit; *UL* = upper limit. * *p* < .05, ** *p* < .01, *** *p* < .001.

## Table 19. Study 1 Inferential Statistics for Preregistered Contrasts on Feeling Thermometer by Condition and Political Orientation

| Effect | Estimate | *t* | *SE* | 95% CI | | η²ₚ | *p* |
| --- | --- | --- | --- | --- | --- | --- | --- |
|  |  |  |  | *LL* | *UL* |  |  |
| Fixed effects |  |  |  |  |  |  |  |
| Intercept | 55.02 | 51.64 | 1.07 | 52.93 | 57.12 | 0.821 | <.001*** |
| Both + Race vs. Other | 3.30 | 3.37 | 0.98 | 1.37 | 5.22 | 0.019 | 0.001** |
| Both + Race vs. None | 2.49 | 2.55 | 0.98 | 0.58 | 4.41 | 0.011 | 0.011* |
| Both vs. Race | -0.35 | -0.21 | 1.67 | -3.64 | 2.94 | <.001 | 0.836 |
| Both + Gender vs. Other | 2.88 | 2.96 | 0.98 | 0.97 | 4.80 | 0.002 | 0.003** |
| Both + Gender vs. None | 2.08 | 2.14 | 0.97 | 0.17 | 4.00 | 0.008 | 0.033 |
| Both vs. Gender | -1.58 | -0.95 | 1.66 | -4.84 | 1.68 | 0.002 | 0.342 |
| Both vs. Other | 10.23 | 3.05 | 3.36 | 3.64 | 16.82 | 0.016 | 0.002** |
| Both vs. None | 7.83 | 2.34 | 3.35 | 1.25 | 14.21 | 0.009 | 0.020* |
| Political Orientation (PO) | -11.38 | -18.91 | 0.60 | -12.56 | -10.20 | 0.381 | <.001*** |
| Both + Race vs. Other X PO | 2.59 | 4.57 | 0.57 | 1.48 | 3.71 | 0.035 | <.001*** |
| Both + Race vs. None X PO | 0.50 | 0.93 | 0.53 | -0.55 | 1.54 | 0.001 | 0.352 |
| Both vs. Race X PO | -0.39 | -0.42 | 0.92 | -2.21 | 1.43 | <.001 | 0.674 |
| Both + Gender vs. Other X PO | 2.52 | 4.44 | 0.57 | 1.41 | 3.64 | 0.033 | <.001*** |
| Both + Gender vs. None X PO | 0.43 | 0.80 | 0.53 | -0.62 | 1.48 | 0.001 | 0.424 |
| Both vs. Gender X PO | -0.60 | -0.64 | 0.93 | -2.43 | 1.23 | 0.001 | 0.521 |
| Both vs. Other X PO | 8.16 | 4.30 | 1.90 | 4.43 | 11.89 | 0.031 | <.001*** |
| Both vs. None X PO | 1.88 | 1.04 | 1.81 | -1.67 | 5.43 | 0.002 | 0.299 |

*Note.*  CI = confidence interval; *LL* = lower limit; *UL* = upper limit. * *p* < .05, ** *p* < .01, *** *p* < .001.

## Table 20. Study 1 Inferential Statistics for Preregistered Contrasts on Political Abilities by Condition and Political Orientation

| Effect | Estimate | *t* | *SE* | 95% CI | | η²ₚ | *p* |
| --- | --- | --- | --- | --- | --- | --- | --- |
|  |  |  |  | *LL* | *UL* |  |  |
| Fixed effects |  |  |  |  |  |  |  |
| Intercept | 3.51 | 79.26 | 0.04 | 3.42 | 3.59 | 0.915 | <.001*** |
| Both + Race vs. Other | 0.24 | 5.85 | 0.04 | 0.16 | 0.32 | 0.056 | <.001*** |
| Both + Race vs. None | 0.07 | 1.80 | 0.04 | -0.01 | 0.15 | 0.006 | 0.073 |
| Both vs. Race | 0.01 | 0.16 | 0.07 | -0.13 | 0.15 | <.001 | 0.877 |
| Both + Gender vs. Other | 0.21 | 5.15 | 0.04 | 0.13 | 0.29 | 0.044 | <.001*** |
| Both + Gender vs. None | 0.04 | 1.09 | 0.04 | -0.04 | 0.12 | 0.002 | 0.278 |
| Both vs. Gender | -0.08 | -1.10 | 0.07 | -0.21 | 0.06 | 0.002 | 0.272 |
| Both vs. Other | 0.70 | 5.04 | 0.14 | 0.43 | 0.98 | 0.042 | <.001*** |
| Both vs. None | 0.21 | 1.49 | 0.14 | -0.07 | 0.48 | 0.004 | 0.136 |
| Political Orientation (PO) | -0.43 | -17.22 | 0.03 | -0.48 | -0.38 | 0.338 | <.001*** |
| Both + Race vs. Other X PO | 0.12 | 5.17 | 0.02 | 0.08 | 0.17 | 0.044 | <.001*** |
| Both + Race vs. None X PO | 0.01 | 0.49 | 0.02 | -0.03 | 0.05 | <.001 | 0.624 |
| Both vs. Race X PO | 0.02 | 0.57 | 0.04 | -0.05 | 0.10 | 0.001 | 0.572 |
| Both + Gender vs. Other X PO | 0.11 | 4.55 | 0.02 | 0.06 | 0.15 | 0.03 | <.001*** |
| Both + Gender vs. None X PO | -0.004 | -0.16 | 0.02 | -0.05 | 0.04 | <.001 | 0.872 |
| Both vs. Gender X PO | -0.02 | -0.56 | 0.04 | -0.10 | 0.05 | 0.001 | 0.577 |
| Both vs. Other X PO | 0.34 | 4.35 | 0.08 | 0.019 | 0.50 | <.001 | <.001*** |
| Both vs. None X PO | 0.01 | 0.14 | 0.08 | -0.14 | 0.16 | <.001 | 0.885 |

*Note.*  CI = confidence interval; *LL* = lower limit; *UL* = upper limit. * *p* < .05, ** *p* < .01, *** *p* < .001.

## Table 21. Study 1 Inferential Statistics for Performance as a Political Leader by Condition and Political Orientation

| Effect | Estimate | *t* | *SE* | 95% CI | | η²ₚ | *p* |
| --- | --- | --- | --- | --- | --- | --- | --- |
|  |  |  |  | *LL* | *UL* |  |  |
| Fixed effects |  |  |  |  |  |  |  |
| Intercept | 3.06 | 64.43 | 0.05 | 2.97 | 3.15 | 0.877 | <.001*** |
| Race vs No Race | -0.11 | -2.06 | 0.05 | -0.21 | -0.01 | 0.007 | 0.040* |
| Gender vs No Gender | -0.06 | -1.05 | 0.05 | -0.16 | 0.05 | 0.002 | 0.294 |
| Gender X Race | 0.04 | 0.81 | 0.05 | -0.06 | 0.15 | 0.001 | 0.420 |
| Harris vs. Other | -0.04 | -1.73 | 0.02 | -0.09 | 0.01 | 0.005 | 0.085 |
| Political Orientation (PO) | -0.57 | -21.30 | 0.03 | -0.62 | -0.52 | 0.438 | <.001*** |
| Race vs No Race X PO | -0.01 | -0.36 | 0.03 | -0.07 | 0.05 | <.001 | 0.721 |
| Gender vs No Gender X PO | 0.01 | 0.28 | 0.03 | -0.05 | 0.07 | <.001 | 0.782 |
| Gender X Race X PO | -0.01 | -0.27 | 0.03 | -0.07 | 0.05 | <.001 | 0.790 |
| Harris vs. Other X PO | -0.02 | -1.25 | 0.01 | -0.05 | 0.01 | 0.003 | 0.213 |

*Note.*  CI = confidence interval; *LL* = lower limit; *UL* = upper limit. * *p* < .05, ** *p* < .01, *** *p* < .001.

## Table 22. Study 1 Inferential Statistics for Perceptions of Harris as Black by Condition and Political Orientation

| Effect | Estimate | *t* | *SE* | 95% CI | | η²ₚ | *p* |
| --- | --- | --- | --- | --- | --- | --- | --- |
|  |  |  |  | *LL* | *UL* |  |  |
| Fixed effects |  |  |  |  |  |  |  |
| Intercept | 3.44 | 72.67 | 0.05 | 3.35 | 3.53 | 0.901 | <.001*** |
| Race vs No Race | -0.05 | -1.02 | 0.05 | -0.16 | 0.05 | 0.002 | 0.309 |
| Gender vs No Gender | -0.003 | -0.05 | 0.05 | -0.11 | 0.10 | <.001 | 0.961 |
| Gender X Race | 0.03 | 0.49 | 0.05 | -0.08 | 0.13 | <.001 | 0.625 |
| Harris vs. Other | -0.02 | -0.92 | 0.02 | -0.07 | 0.03 | 0.001 | 0.358 |
| Political Orientation (PO) | -0.30 | -11.17 | 0.03 | -0.35 | -0.25 | 0.176 | <.001*** |
| Race vs No Race X PO | 0.003 | 0.11 | 0.03 | -0.06 | 0.06 | <.001 | 0.912 |
| Gender vs No Gender X PO | 0.01 | 0.49 | 0.03 | -0.04 | 0.07 | <.001 | 0.625 |
| Gender X Race X PO | -0.03 | -0.85 | 0.03 | -0.08 | 0.03 | 0.001 | 0.397 |
| Harris vs. Other X PO | -0.01 | -0.99 | 0.01 | -0.04 | 0.01 | 0.002 | 0.322 |

*Note.*  CI = confidence interval; *LL* = lower limit; *UL* = upper limit. * *p* < .05, ** *p* < .01, *** *p* < .001.

## Table 23. Study 1 Inferential Statistics for Perceptions of Harris as South Asian by Condition and Political Orientation

| Effect | Estimate | *t* | *SE* | 95% CI | | η²ₚ | *p* |
| --- | --- | --- | --- | --- | --- | --- | --- |
|  |  |  |  | *LL* | *UL* |  |  |
| Fixed effects |  |  |  |  |  |  |  |
| Intercept | 3.41 | 73.28 | 0.05 | 3.32 | 3.50 | 0.902 | <.001*** |
| Race vs No Race | 0.08 | 1.51 | 0.05 | -0.02 | 0.18 | 0.004 | 0.133 |
| Gender vs No Gender | -0.05 | -1.02 | 0.05 | -0.16 | 0.05 | 0.002 | 0.308 |
| Gender X Race | 0.01 | 0.13 | 0.05 | -0.10 | 0.11 | <.001 | 0.896 |
| Harris vs. Other | 0.01 | 0.33 | 0.02 | -0.04 | 0.05 | <.001 | 0.741 |
| Political Orientation (PO) | -0.20 | -7.54 | 0.03 | -0.25 | -0.15 | 0.089 | <.001*** |
| Race vs No Race X PO | 0.03 | 1.19 | 0.03 | -0.02 | 0.09 | 0.002 | 0.235 |
| Gender vs No Gender X PO | -0.03 | -1.06 | 0.03 | -0.09 | 0.03 | 0.002 | 0.290 |
| Gender X Race X PO | -0.05 | -1.84 | 0.03 | -0.11 | 0.004 | 0.006 | 0.067 |
| Harris vs. Other X PO | -0.01 | -1.04 | 0.01 | -0.04 | 0.01 | 0.002 | 0.300 |

*Note.*  CI = confidence interval; *LL* = lower limit; *UL* = upper limit. * *p* < .05, ** *p* < .01, *** *p* < .001.

## Table 24. Study 1 Inferential Statistics for Harris’s Perceptions of Herself as Black by Condition and Political Orientation

| Effect | Estimate | *t* | *SE* | 95% CI | | η²ₚ | *p* |
| --- | --- | --- | --- | --- | --- | --- | --- |
|  |  |  |  | *LL* | *UL* |  |  |
| Fixed effects |  |  |  |  |  |  |  |
| Intercept | 3.77 | 80.12 | 0.05 | 3.67 | 3.86 | 0.917 | <.001*** |
| Race vs No Race | -0.02 | -0.42 | 0.05 | -0.13 | 0.08 | <.001 | 0.675 |
| Gender vs No Gender | -0.02 | -0.44 | 0.05 | -0.13 | 0.08 | <.001 | 0.664 |
| Gender X Race | 0.02 | 0.31 | 0.05 | -0.09 | 0.12 | <.001 | 0.758 |
| Harris vs. Other | -0.01 | -0.44 | 0.02 | -0.06 | 0.04 | <.001 | 0.663 |
| Political Orientation (PO) | -0.15 | -5.48 | 0.03 | -0.20 | -0.09 | 0.049 | <.001*** |
| Race vs No Race X PO | 0.01 | 0.46 | 0.03 | -0.04 | 0.07 | <.001 | 0.646 |
| Gender vs No Gender X PO | -0.004 | -0.14 | 0.03 | -0.06 | 0.05 | <.001 | 0.891 |
| Gender X Race X PO | -0.01 | -0.48 | 0.03 | -0.07 | 0.04 | <.001 | 0.634 |
| Harris vs. Other X PO | 0.005 | 0.35 | 0.01 | -0.02 | 0.03 | <.001 | 0.725 |

*Note.*  CI = confidence interval; *LL* = lower limit; *UL* = upper limit. * *p* < .05, ** *p* < .01, *** *p* < .001.

## Table 25. Study 1 Inferential Statistics for Harris’s Perceptions of Viewing Herself as South Asian by Condition and Political Orientation

| Effect | Estimate | *t* | *SE* | 95% CI | | η²ₚ | *p* |
| --- | --- | --- | --- | --- | --- | --- | --- |
|  |  |  |  | *LL* | *UL* |  |  |
| Fixed effects |  |  |  |  |  |  |  |
| Intercept | 3.44 | 76.63 | 0.05 | 3.36 | 3.53 | 0.910 | <.001*** |
| Race vs No Race | 0.04 | 0.83 | 0.05 | -0.06 | 0.14 | 0.001 | 0.408 |
| Gender vs No Gender | 0.01 | 0.16 | 0.05 | -0.09 | 0.11 | <.001 | 0.872 |
| Gender X Race | 0.03 | 0.62 | 0.05 | -0.07 | 0.13 | 0.001 | 0.533 |
| Harris vs. Other | -0.01 | -0.25 | 0.02 | -0.05 | 0.04 | <.001 | 0.800 |
| Political Orientation (PO) | -0.26 | -10.20 | 0.03 | -0.31 | -0.21 | 0.152 | <.001*** |
| Race vs No Race X PO | 0.02 | 0.87 | 0.03 | -0.03 | 0.08 | 0.001 | 0.388 |
| Gender vs No Gender X PO | -0.01 | -0.49 | 0.03 | -0.07 | 0.04 | <.001 | 0.622 |
| Gender X Race X PO | -0.05 | -1.86 | 0.03 | -0.11 | 0.003 | 0.006 | 0.063 |
| Harris vs. Other X PO | -0.02 | -1.68 | 0.01 | -0.05 | 0.004 | 0.005 | 0.094 |

*Note.*  CI = confidence interval; *LL* = lower limit; *UL* = upper limit. * *p* < .05, ** *p* < .01, *** *p* < .001.

## Table 26. Study 1 Inferential Statistics for Positive Traits by Condition and Participant Gender

| Effect | Estimate | *t* | *SE* | 95% CI | | η²ₚ | *p* |
| --- | --- | --- | --- | --- | --- | --- | --- |
|  |  |  |  | *LL* | *UL* |  |  |
| Fixed effects |  |  |  |  |  |  |  |
| Intercept | 3.48 | 83.18 | 0.04 | 3.39 | 3.56 | 0.922 | <.001*** |
| Race vs No Race | -0.10 | -2.08 | 0.05 | -0.19 | -0.01 | 0.007 | 0.038* |
| Gender vs No Gender | 0.06 | 1.20 | 0.05 | -0.04 | 0.15 | 0.002 | 0.229 |
| Gender X Race | -0.001 | -0.02 | 0.05 | -0.09 | 0.09 | <.001 | 0.986 |
| Harris vs. Other | 0.06 | 3.01 | 0.02 | 0.02 | 0.11 | 0.015 | 0.003** |
| Participant Gender (PG) | 0.20 | 2.35 | 0.08 | 0.03 | 0.36 | 0.009 | 0.019* |
| Race vs No Race X PG | -0.17 | -1.85 | 0.09 | -0.36 | 0.01 | 0.006 | 0.065 |
| Gender vs No Gender X PG | -0.06 | -0.61 | 0.09 | -0.24 | 0.13 | 0.001 | 0.544 |
| Gender X Race X PG | 0.05 | 0.58 | 0.09 | -0.13 | 0.24 | 0.001 | 0.563 |
| Harris vs. Other X PG | 0.05 | 1.14 | 0.04 | -0.04 | 0.13 | 0.002 | 0.256 |

*Note.*  CI = confidence interval; *LL* = lower limit; *UL* = upper limit. * *p* < .05, ** *p* < .01, *** *p* < .001.

## Table 27. Study 1 Inferential Statistics for Negative Traits by Condition and Participant Gender

| Effect | Estimate | *t* | *SE* | 95% CI | | η²ₚ | *p* |
| --- | --- | --- | --- | --- | --- | --- | --- |
|  |  |  |  | *LL* | *UL* |  |  |
| Fixed effects |  |  |  |  |  |  |  |
| Intercept | 3.09 | 76.38 | 0.04 | 3.01 | 3.17 | 0.909 | <.001*** |
| Race vs No Race | 0.11 | 2.36 | 0.05 | 0.02 | 0.20 | 0.009 | 0.019* |
| Gender vs No Gender | -0.02 | -0.53 | 0.05 | -0.11 | 0.07 | <.001 | 0.594 |
| Gender X Race | 0.02 | 0.49 | 0.05 | -0.07 | 0.11 | <.001 | 0.623 |
| Harris vs. Other | -0.003 | -0.14 | 0.02 | -0.04 | 0.04 | <.001 | 0.892 |
| Participant Gender (PG) | -0.21 | -2.56 | 0.08 | -0.37 | -0.05 | 0.011 | 0.011* |
| Race vs No Race X PG | -0.05 | -0.52 | 0.09 | -0.22 | 0.13 | <.001 | 0.607 |
| Gender vs No Gender X PG | 0.01 | 0.14 | 0.09 | -0.16 | 0.19 | <.001 | 0.885 |
| Gender X Race X PG | 0.09 | 0.96 | 0.09 | -0.09 | 0.26 | 0.002 | 0.335 |
| Harris vs. Other X PG | -0.02 | -0.53 | 0.04 | -0.10 | 0.06 | <.001 | 0.596 |

*Note.*  CI = confidence interval; *LL* = lower limit; *UL* = upper limit. * *p* < .05, ** *p* < .01, *** *p* < .001.

## Table 28. Study 1 Inferential Statistics for Feeling Thermometer by Condition and Participant Gender

| Effect | Estimate | *t* | *SE* | 95% CI | | η²ₚ | *p* |
| --- | --- | --- | --- | --- | --- | --- | --- |
|  |  |  |  | *LL* | *UL* |  |  |
| Fixed effects |  |  |  |  |  |  |  |
| Intercept | 55.12 | 40.01 | 1.38 | 52.41 | 57.83 | 0.733 | <.001*** |
| Race vs No Race | -2.63 | -1.71 | 1.54 | -5.65 | 0.39 | 0.005 | 0.088 |
| Gender vs No Gender | -0.29 | -0.19 | 1.54 | -3.31 | 2.73 | <.001 | 0.851 |
| Gender X Race | 0.47 | 0.31 | 1.54 | -2.55 | 3.49 | <.001 | 0.759 |
| Harris vs. Other | 1.31 | 1.89 | 0.69 | -0.05 | 2.67 | 0.006 | 0.059 |
| Participant Gender (PG) | 5.59 | 2.03 | 2.76 | 0.18 | 11.00 | 0.007 | 0.043* |
| Race vs No Race X PG | -3.32 | -1.08 | 3.08 | -9.35 | 2.72 | 0.002 | 0.281 |
| Gender vs No Gender X PG | -0.22 | -0.07 | 3.08 | -6.26 | 5.82 | <.001 | 0.942 |
| Gender X Race X PG | 1.24 | 0.40 | 3.08 | -4.80 | 7.28 | <.001 | 0.687 |
| Harris vs. Other X PG | 0.83 | 0.60 | 1.39 | -1.89 | 3.55 | 0.001 | 0.551 |

*Note.*  CI = confidence interval; *LL* = lower limit; *UL* = upper limit. * *p* < .05, ** *p* < .01, *** *p* < .001.

## Table 29. Study 1 Inferential Statistics for Political Abilities by Condition and Participant Gender

| Effect | Estimate | *t* | *SE* | 95% CI | | η²ₚ | *p* |
| --- | --- | --- | --- | --- | --- | --- | --- |
|  |  |  |  | *LL* | *UL* |  |  |
| Fixed effects |  |  |  |  |  |  |  |
| Intercept | 3.51 | 63.24 | 0.06 | 3.40 | 3.62 | 0.873 | <.001*** |
| Race vs No Race | -0.10 | -1.55 | 0.06 | -0.22 | 0.03 | 0.004 | 0.122 |
| Gender vs No Gender | 0.03 | 0.50 | 0.06 | -0.09 | 0.15 | <.001 | 0.616 |
| Gender X Race | -0.001 | -0.01 | 0.06 | -0.12 | 0.12 | <.001 | 0.992 |
| Harris vs. Other | 0.12 | 4.31 | 0.03 | 0.07 | 0.18 | 0.031 | <.001*** |
| Participant Gender (PG) | 0.20 | 1.77 | 0.11 | -0.02 | 0.42 | 0.005 | 0.077 |
| Race vs No Race X PG | -0.19 | -1.56 | 0.12 | -0.44 | 0.05 | 0.004 | 0.118 |
| Gender vs No Gender X PG | -0.06 | -0.45 | 0.12 | -0.30 | 0.19 | <.001 | 0.651 |
| Gender X Race X PG | -0.003 | -0.02 | 0.12 | -0.25 | 0.24 | <.001 | 0.981 |
| Harris vs. Other X PG | 0.05 | 0.87 | 0.06 | -0.06 | 0.16 | 0.001 | 0.385 |

*Note.*  CI = confidence interval; *LL* = lower limit; *UL* = upper limit. * *p* < .05, ** *p* < .01, *** *p* < .001.

## Table 30. Study 1 Inferential Statistics for Positive Traits by Condition and Participant Race

| Effect | Estimate | *t* | *SE* | 95% CI | | η²ₚ | *p* |
| --- | --- | --- | --- | --- | --- | --- | --- |
|  |  |  |  | *LL* | *UL* |  |  |
| Fixed effects |  |  |  |  |  |  |  |
| Intercept | 3.48 | 80.53 | 0.04 | 3.39 | 3.56 | 0.918 | <.001*** |
| Race vs No Race | -0.10 | -2.06 | 0.05 | -0.19 | -0.01 | 0.007 | 0.040* |
| Gender vs No Gender | 0.05 | 1.03 | 0.05 | -0.05 | 0.14 | 0.002 | 0.305 |
| Gender X Race | 0.01 | 0.21 | 0.05 | -0.08 | 0.10 | <.001 | 0.836 |
| Harris vs. Other | 0.06 | 2.76 | 0.02 | 0.02 | 0.11 | 0.013 | 0.006** |
| Participant Race (PR) | 0.04 | 0.41 | 0.09 | -0.13 | 0.21 | <.001 | 0.684 |
| Race vs No Race X PR | -0.07 | -0.69 | 0.10 | -0.25 | 0.12 | 0.001 | 0.493 |
| Gender vs No Gender X PR | -0.002 | -0.03 | 0.10 | -0.19 | 0.19 | <.001 | 0.980 |
| Gender X Race X PR | -0.03 | -0.32 | 0.10 | -0.22 | 0.16 | <.001 | 0.749 |
| Harris vs. Other X PR | -0.02 | -0.42 | 0.05 | -0.11 | 0.07 | <.001 | 0.679 |

*Note.*  CI = confidence interval; *LL* = lower limit; *UL* = upper limit. * *p* < .05, ** *p* < .01, *** *p* < .001.

## Table 31. Study 1 Inferential Statistics for Negative Traits by Condition and Participant Race

| Effect | Estimate | *t* | *SE* | 95% CI | | η²ₚ | *p* |
| --- | --- | --- | --- | --- | --- | --- | --- |
|  |  |  |  | *LL* | *UL* |  |  |
| Fixed effects |  |  |  |  |  |  |  |
| Intercept | 3.07 | 74.20 | 0.04 | 2.99 | 3.15 | 0.904 | <.001*** |
| Race vs No Race | 0.11 | 2.38 | 0.05 | 0.02 | 0.20 | 0.010 | 0.018* |
| Gender vs No Gender | -0.03 | -0.55 | 0.05 | -0.12 | 0.07 | 0.001 | 0.586 |
| Gender X Race | 0.04 | 0.93 | 0.05 | -0.05 | 0.13 | 0.001 | 0.351 |
| Harris vs. Other | 0.01 | 0.61 | 0.02 | -0.03 | 0.06 | 0.001 | 0.541 |
| Participant Race (PR) | 0.15 | 1.86 | 0.08 | -0.01 | 0.32 | 0.006 | 0.064 |
| Race vs No Race X PR | -0.02 | -0.25 | 0.09 | -0.20 | 0.16 | <.001 | 0.800 |
| Gender vs No Gender X PR | 0.01 | 0.11 | 0.09 | -0.17 | 0.19 | <.001 | 0.910 |
| Gender X Race X PR | -0.08 | -0.84 | 0.09 | -0.26 | 0.10 | 0.001 | 0.400 |
| Harris vs. Other X PR | -0.09 | -2.10 | 0.04 | -0.17 | -0.01 | 0.008 | 0.036* |

*Note.*  CI = confidence interval; *LL* = lower limit; *UL* = upper limit. * *p* < .05, ** *p* < .01, *** *p* < .001.

## Table 32. Study 1 Inferential Statistics for Feeling Thermometer by Condition and Participant Race

| Effect | Estimate | *t* | *SE* | 95% CI | | η²ₚ | *p* |
| --- | --- | --- | --- | --- | --- | --- | --- |
|  |  |  |  | *LL* | *UL* |  |  |
| Fixed effects |  |  |  |  |  |  |  |
| Intercept | 55.25 | 38.97 | 1.42 | 52.47 | 58.04 | 0.723 | <.001*** |
| Race vs No Race | -2.72 | -1.74 | 1.57 | -5.80 | 0.36 | 0.005 | 0.083 |
| Gender vs No Gender | -0.49 | -0.31 | 1.57 | -3.57 | 2.59 | <.001 | 0.754 |
| Gender X Race | 0.57 | 0.37 | 1.57 | -2.51 | 3.65 | <.001 | 0.715 |
| Harris vs. Other | 1.19 | 1.62 | 0.73 | -0.25 | 2.63 | 0.004 | 0.106 |
| Participant Race (PR) | -0.72 | -0.25 | 2.84 | -6.28 | 4.85 | <.001 | 0.801 |
| Race vs No Race X PR | -0.91 | -0.29 | 3.13 | -7.06 | 5.24 | <.001 | 0.771 |
| Gender vs No Gender X PR | 0.10 | 0.32 | 3.13 | -5.15 | 7.15 | <.001 | 0.750 |
| Gender X Race X PR | -0.10 | -0.03 | 3.13 | -6.25 | 6.06 | <.001 | 0.975 |
| Harris vs. Other X PR | 0.27 | 0.18 | 1.47 | -2.61 | 3.15 | <.001 | 0.854 |

*Note.*  CI = confidence interval; *LL* = lower limit; *UL* = upper limit. * *p* < .05, ** *p* < .01, *** *p* < .001.

## Table 33. Study 1 Inferential Statistics for Political Abilities by Condition and Participant Race

| Effect | Estimate | *t* | *SE* | 95% CI | | η²ₚ | *p* |
| --- | --- | --- | --- | --- | --- | --- | --- |
|  |  |  |  | *LL* | *UL* |  |  |
| Fixed effects |  |  |  |  |  |  |  |
| Intercept | 3.51 | 61.50 | 0.06 | 3.40 | 3.63 | 0.867 | <.001*** |
| Race vs No Race | -0.10 | -1.55 | 0.06 | -0.22 | 0.03 | 0.004 | 0.122 |
| Gender vs No Gender | 0.03 | 0.46 | 0.06 | -0.10 | 0.15 | <.001 | 0.649 |
| Gender X Race | 0.01 | 0.11 | 0.06 | -0.12 | 0.13 | <.001 | 0.909 |
| Harris vs. Other | 0.12 | 3.92 | 0.03 | 0.06 | 0.17 | 0.026 | <.001*** |
| Participant Race (PR) | -0.01 | -0.08 | 0.11 | -0.23 | 0.22 | <.001 | 0.993 |
| Race vs No Race X PR | -0.07 | -0.54 | 0.13 | -0.32 | 0.18 | <.001 | 0.593 |
| Gender vs No Gender X PR | -0.04 | -0.30 | 0.13 | -0.29 | 0.21 | <.001 | 0.766 |
| Gender X Race X PR | -0.04 | -0.30 | 0.13 | -0.29 | 0.21 | <.001 | 0.761 |
| Harris vs. Other X PR | 0.003 | 0.06 | 0.06 | -0.11 | 0.12 | <.001 | 0.955 |

*Note.*  CI = confidence interval; *LL* = lower limit; *UL* = upper limit. * *p* < .05, ** *p* < .01, *** *p* < .001.

# Supplemental Study 2 Materials

Study 2 contained the following deviations from preregistration (<https://osf.io/cgpy8/overview?view_only=a4c2eeffc16548d08175597856b1d152)>.

1. We intended to collect data from 600 participants but stopped data collection at 592 on 10/27/24 because early voting was beginning in some states. Our preregistered target sample size based on an a priori power analysis was 540 so we had exceeded that target when we stopped data collection and our sample for analysis after data exclusion of 588 exceeded our preregistered target sample size.
2. Lack of variation in trait ratings was a preregistered exclusion criteria to detect inattentiveness. However, during data analysis we realized that we had erroneously presented participants with all semantic trait pairs coded in the same direction (i.e., all positive traits were on one side and all negative on the other). Given the partisan nature of the judgments, answering all items in the same way could plausibly reflect a consistent impression of Harris across all traits rather than inattention (i.e., someone answering *5* on each item could have a uniform impression of Harris or could have been failing to closely read the items). To more unambiguously assess inattentiveness, we added ratings of political abilities to our consideration of straight line responding suggestive of inattention. This was useful because the political ability items were coded in the opposite direction from the traits (higher ratings on the traits indicated greater negativity whereas higher rating on the political abilities indicated greater favorability). Anyone straight-lining on traits was omitted from analyses only if they answered all political ability questions in a numerically consistent way (e.g., answering *5* for all trait and political ability items) since doing so indicates evaluatively inconsistent responses between the two measures.
3. We specified 8 contrasts in the preregistration to test all hypotheses. As in Study 1, we subsequently realized we could test the hypotheses more efficiently with a smaller set of contrasts that also had the benefit of corresponding to tests of the main effect of specifying Harris’s Black ancestry, the main effect of specifying her South Asian ancestry, and their interaction (with an additional contrast to compare the other condition to all Harris conditions). For efficiency and conceptual ease, we report the smaller set of contrasts in the main text. The preregistered contrasts can be found in Tables 42-47 in this document.

Table 34 shows the full stimulus materials describing either Kamala Harris or the unnamed other candidate and Table 35 shows the ancillary measures not reported in the main text.

## Table 34. Study 2 Priming Paragraph Text

| Condition | Text |
| --- | --- |
| Black and South Asian Identity | Kamala Harris is the Vice President of the United States, making history as the first Black and South Asian American to hold the office. Her groundbreaking achievement represents a significant step forward for racial equality in U.S. leadership. Prior to this role, Harris was a U.S. Senator from California and the state's Attorney General. She has consistently advocated for civil rights, criminal justice reform, and healthcare access. Harris’s leadership reflects the increasing racial diversity in American political representation. |
| Black Identity | Kamala Harris is the Vice President of the United States, making history as the first Black American to hold the office. Her groundbreaking achievement represents a significant step forward for racial equality in U.S. leadership. Prior to this role, Harris was a U.S. Senator from California and the state’s Attorney General. She has consistently advocated for civil rights, criminal justice reform, and health care access. Harris’s leadership reflects the increasing racial diversity in American political representation. |
| South Asian Identity | Kamala Harris is the Vice President of the United States, making history as the first South Asian American to hold the office. Her groundbreaking achievement represents a significant step forward for racial equality in U.S. leadership. Prior to this role, Harris was a U.S. Senator from California and the state's Attorney General. She has consistently advocated for civil rights, criminal justice reform, and healthcare access. Harris’s leadership reflects the increasing racial diversity in American political representation. |
| No mention of Black and South Asian Identity | Kamala Harris is the Vice President of the United States, bringing extensive experience in public service to the role. Prior to this role, Harris was a U.S. Senator from California and the state's Attorney General. She has consistently advocated for civil rights, criminal justice reform, and healthcare access. Harris’s leadership reflects a significant milestone in American political history, reflecting her commitment to justice and equality. |
| Other candidate | A current politician who is up for reelection brings extensive experience in public service to the role. This politician is a U.S. Senator from New York and the state's Attorney General. They have consistently advocated for civil rights, criminal justice reform, and healthcare access. Their leadership reflects a significant milestone in American political history, reflecting their commitment to justice and equality. |

## Table 35. Study 2 Additional Measures

| Measure | Item | Response Options |
| --- | --- | --- |
| Registration | Are you registered to vote? | *Yes, No* |
| Party | What political party are you registered to vote with, if any? | *1 = Democratic Party, 2 = Republican Party, 3 = None, 4 = Independent, 5 = Other* |
| Voter Registration Intention | Do you intend to register to vote before the November 5th, 2024 elections or do you not intend to register | *Yes, intend to register*  *No, do not intend to register* |
| Registered Voter State | In what state are you registered to vote now? | *Select a State* |
| Voting in the Presidential Primary | In the presidential primary or caucus, who did you vote for? | *1 = Joe Biden, 2 = Another Democrat, 3 = Donald Trump, 4 = Another Republican, 5 = Someone else who is not a Republican or a Democrat, 6 = Did not vote* |
| Media Consumption | Please select all of the news outlets that you regularly watch/read: | *Jacobin, Nation, Democracy Now!, Salon, The Intercept,*  *Mother Jones, MSNBC, Slate, HuffPost, Vox,*  *Atlantic, New York Times, Guardian, Washington Post, CNN, Yahoo! News, NBC, NPR, PBS, USA Today,*  *CBS, ABC, Wall Street Journal, Dispatch, Reason,*  *New York Post, The Libertarian, Washington Examiner, Fox News,*  *Washington Times, Daily Wire, National Review, Blaze, Newsmax,*  *Breitbart, American Conservative, Daily Caller* |
| Media Consumption 2 | How often do you consume media (read, watch, etc.) on the following platforms? | *Of those selected on the prior question 1 = Occasionally to 7 = Daily* |
| Harris’s Performance | Overall, how would you rate Kamala Harris's performance as a political leader? | *1 = Very Negative to*  *5 = Very Positive* |
| Harris DEI Hire | To what degree do you consider Kamala Harris as a DEI (Diversity, Equity and Inclusion) Hire? | *1 = strongly disagree to 7 = strongly agree* |
| DEI Hire Reasoning | Can you tell us more about why you chose selected choices? | Open ended response |
| Harris DEI Reasoning | Why do you think Kamala Harris is a DEI Hire? Because of… (Check all that Apply) | *1 = Her Gender, 2 = Her Black Heritage, 3 = Her South Asian heritage* |
| DEI Hire Definition | Under what circumstances do you consider someone a DEI Hire? | Open ended response |
| Perceptions of Harris (Black | To what degree do you think Kamala Harris is Black? | *1 = Not at all - 5 = A great deal* |
| Perceptions of Harris (South Asian) | To what degree do you think Kamala Harris is South Asian? | *1 = Not at all - 5 = A great deal* |
| Harris’s perceptions of Her Black Heritage | To what degree do you think Kamala Harris views herself as Black? | *1 = Not at all - 5 = A great deal* |
| Harris’s perceptions of Her South Asian Heritage | To what degree do you think Kamala Harris views herself as South Asian? | *1 = Not at all - 5 = A great deal* |
| South Asian American Group Superiority Ratings | People like me think South Asian Americans are: | *1 = Inferior to 5 = Superior* |
| South Asian American Group Foreignness Ratings | People like me think South Asian Americans are: | *1 = American to 5 = Foreign* |
| Black American Group Superiority Ratings | People like me think Black Americans are: | *1 = Inferior to 5 = Superior* |
| Black American Group Foreignness Ratings | People like me think Black Americans are: | *1 = American to 5 = Foreign* |
| Afrocentricity | To what degree do you think Kamala Harris has Afrocentric features, meaning facial features that are stereotypical of Black individuals? Move the slider below to represent your perception of her facial features | *0 = Not at all Afrocentric to 100 = Completely Afrocentric* |
| South Asian Features | To what degree do you think Kamala Harris has South Asian features, meaning facial features that are stereotypical of South Asian individuals? Move the slider below to represent your perception of her facial features | *0 = Not at all South Asian to 100 = Completely South Asian* |
| Reasons to Vote for Kamala | What reasons might make you want to vote for Kamala Harris? | Open ended response |
| Reasons to Vote Against Kamala | What reasons might make you want to NOT vote for Kamala Harris? | Open ended response |
| Harris’s Overall Performance | Overall, how would you rate Kamala Harris's performance as a political leader? | 1 = *Very negative –* 5 *= Very positive* |
| Harris’s Race | What do you think Kamala Harris's race is? | Open ended response |

# Supplemental Study 2 Analyses

Data and R code are available at (<https://osf.io/aq26w/?view_only=2a8bc42887204afe87981eda81ab464b>).

Tables 36-41 present the full inferential statistics for the traits, feeling thermometer, political abilities, superiority/inferiority, and American/foreign ratings that are discussed in the main text.

The preregistered contrast codes are shown in Table 42, then Tables 43-47 report the main dependent measures of feeling thermometer, political ability, superiority/inferiority, and American/foreign ratings using the preregistered contrasts.

Tables 48-58 report analyses on additional measures not reported in the main text.

For exploratory purposes, we replicated the analyses reported in the main text with participant gender and then with participant race as moderators. Gender was coded Man = -1/2, Not Man = 1/2, and race was coded Person of Color = -1/2, Not Person of Color = 1/2. As seen in Tables 59-68, neither participant gender nor race moderated any of the effects reported in the main text.

## Table 36. Study 2 Descriptive Statistics for Outcomes by Condition

| Condition | Traits  *M* (*SD*) | Political Abilities  *M* (*SD*) | Feeling Thermometer  *M* (*SD*) | Superiority  *M* (*SD*) | Americanness  *M* (*SD*) |
| --- | --- | --- | --- | --- | --- |
| Black | 3.38 (1.30) | 3.14 (1.46) | 49.22 (36.39) | 2.99 (1.21) | 4.23 (1.19) |
| South Asian | 3.63 (1.22) | 3.42 (1.37) | 57.58 (34.91) | 3.30 (1.18) | 4.21 (1.10) |
| Both | 3.45 (1.33) | 3.28 (1.47) | 51.67 (37.08) | 3.19 (1.32) | 4.12 (1.19) |
| None | 3.55 (1.26) | 3.38 (1.41) | 53.91 (36.55) | 3.22 (1.19) | 4.12 (1.24) |
| Other | 4.17 (0.64) | 4.27 (0.66) | 68.55 (20.27) | 3.66 (0.91) | 4.54 (0.76) |

*Note.*  Values represent raw means (*M*) and standard deviations (*SD*) for each outcome across the five experimental conditions.

## Table 37. Study 2 Inferential Statistics for Trait Ratings by Condition and Political Orientation

| Effect | Estimate | *t* | *SE* | 95% CI | | η²ₚ | *p* |
| --- | --- | --- | --- | --- | --- | --- | --- |
|  |  |  |  | *LL* | *UL* |  |  |
| Fixed effects |  |  |  |  |  |  |  |
| Intercept | 3.64 | 96.39 | 0.04 | 3.57 | 3.72 | 0.941 | <.001*** |
| Black vs. Not Black | -0.08 | -2.00 | 0.04 | -0.17 | -0.001 | 0.007 | 0.046* |
| South Asian vs. Not South Asian | 0.003 | 0.07 | 0.04 | -0.08 | 0.09 | <.001 | 0.941 |
| Black X South Asian | 0.002 | 0.06 | 0.04 | -0.09 | 0.09 | <.001 | 0.956 |
| Harris vs. Other | 0.13 | 6.67 | 0.02 | 0.09 | 0.16 | 0.072 | <.001*** |
| Political Orientation (PO) | -0.39 | -18.73 | 0.02 | -0.43 | -0.35 | 0.378 | <.001*** |
| Black vs. Not Black X PO | -0.05 | -1.90 | 0.02 | -0.09 | 0.002 | 0.006 | 0.058 |
| South Asian vs. Not South  Asian X PO | -0.02 | -0.79 | 0.02 | -0.07 | 0.03 | 0.001 | 0.433 |
| Black X South Asian X PO | 0.002 | 0.07 | 0.02 | -0.04 | 0.05 | <.001 | 0.942 |
| Harris vs. Other X PO | 0.07 | 6.52 | 0.01 | 0.05 | 0.09 | 0.069 | <.001*** |

*Note.*  CI = confidence interval; *LL* = lower limit; *UL* = upper limit. * *p* < .05, ** *p* < .01, *** *p* < .001.

## Table 38. Study 2 Inferential Statistics for Thermometer Ratings by Condition and Political Orientation

| Effect | Estimate | *t* | *SE* | 95% CI | | η²ₚ | *p* |
| --- | --- | --- | --- | --- | --- | --- | --- |
|  |  |  |  | *LL* | *UL* |  |  |
| Fixed effects |  |  |  |  |  |  |  |
| Intercept | 56.40 | 50.84 | 1.11 | 54.22 | 58.58 | 0.817 | <.001*** |
| Black vs. Not Black | -2.56 | -2.07 | 1.24 | -4.99 | -0.13 | 0.007 | 0.039* |
| South Asian vs. Not South Asian | 0.48 | 0.39 | 1.24 | -1.95 | 2.91 | <.001 | 0.700 |
| Black X South Asian | -0.12 | -0.10 | 1.24 | -2.55 | 2.31 | <.001 | 0.924 |
| Harris vs. Other | 2.89 | 5.18 | 0.56 | 1.80 | 3.99 | 0.044 | <.001*** |
| Political Orientation (PO) | -11.02 | -17.94 | 0.61 | -12.22 | -9.81 | 0.358 | <.001*** |
| Black vs. Not Black X PO | -0.51 | -0.74 | 0.69 | -1.87 | 0.84 | 0.001 | 0.460 |
| South Asian vs. Not South Asian  X PO | -0.33 | -0.48 | 0.69 | -1.69 | 1.03 | <.001 | 0.633 |
| Black X South Asian X PO | 0.49 | 0.71 | 0.69 | -0.87 | 1.85 | 0.001 | 0.478 |
| Harris vs. Other X PO | 1.74 | 5.74 | 0.30 | 1.14 | 2.33 | 0.054 | 0.460 |

*Note.*  CI = confidence interval; *LL* = lower limit; *UL* = upper limit. * *p* < .05, ** *p* < .01, *** *p* < .001.

## Table 39. Study 2 Inferential Statistics for Political Abilities by Condition and Political Orientation

| Effect | Estimate | *t* | *SE* | 95% CI | | η²ₚ | *p* |
| --- | --- | --- | --- | --- | --- | --- | --- |
|  |  |  |  | *LL* | *UL* |  |  |
| Fixed effects |  |  |  |  |  |  |  |
| Intercept | 3.51 | 83.24 | 0.04 | 3.42 | 3.59 | 0.923 | <.001*** |
| Black vs. Not Black | -0.09 | -1.90 | 0.05 | -0.18 | 0.003 | 0.006 | 0.058 |
| South Asian vs. Not South  Asian | 0.01 | 0.11 | 0.05 | -0.09 | 0.10 | <.001 | 0.911 |
| Black X South Asian | 0.03 | 0.71 | 0.05 | -0.06 | 0.13 | <.001 | 0.476 |
| Harris vs. Other | 0.19 | 8.83 | 0.02 | 0.15 | 0.23 | 0.119 | <.001*** |
| Political Orientation (PO) | -0.43 | -18.58 | 0.02 | -0.48 | -0.39 | 0.374 | <.001*** |
| Black vs. Not Black X PO | -0.03 | -1.23 | 0.03 | -0.08 | 0.02 | 0.003 | 0.221 |
| South Asian vs. Not South  Asian X PO | -0.01 | -0.49 | 0.03 | -0.06 | 0.04 | <.001 | 0.622 |
| Black X South Asian X PO | 0.02 | 0.60 | 0.03 | -0.04 | 0.07 | 0.001 | 0.551 |
| Harris vs. Other X PO | 0.08 | 6.90 | 0.01 | 0.06 | 0.10 | 0.076 | <.001*** |

*Note.*  CI = confidence interval; *LL* = lower limit; *UL* = upper limit. * *p* < .05, ** *p* < .01, *** *p* < .001.

## Table 40. Study 2 Inferential Statistics for Superiority/Inferiority Ratings by Condition and Political Orientation

| Effect | Estimate | *t* | *SE* | 95% CI | | η²ₚ | *p* |
| --- | --- | --- | --- | --- | --- | --- | --- |
|  |  |  |  | *LL* | *UL* |  |  |
| Fixed effects |  |  |  |  |  |  |  |
| Intercept | 3.28 | 79.09 | 0.04 | 3.19 | 3.36 | 0.915 | <.001*** |
| Black vs. Not Black | -0.08 | -1.69 | 0.05 | -0.17 | 0.01 | 0.005 | 0.091 |
| South Asian vs. Not South  Asian | 0.04 | 0.92 | 0.05 | -0.05 | 0.13 | 0.001 | 0.360 |
| Black X South Asian | 0.03 | 0.74 | 0.05 | -0.06 | 0.13 | 0.001 | 0.461 |
| Harris vs. Other | 0.09 | 4.4 | 0.02 | 0.05 | 0.13 | 0.032 | <.001*** |
| Political Orientation (PO) | -0.31 | -13.53 | 0.02 | -0.26 | -0.27 | 0.241 | <.001*** |
| Black vs. Not Black X PO | -0.05 | -2.02 | 0.03 | -0.10 | 0.001 | 0.007 | 0.044* |
| South Asian vs. Not South  Asian X PO | -0.05 | -01.98 | 0.03 | -0.10 | <.001 | 0.007 | 0.048* |
| Black X South Asian X PO | 0.001 | 0.03 | 0.03 | -0.05 | 0.05 | <.001 | 0.979 |
| Harris vs. Other X PO | 0.06 | 4.85 | 0.01 | 0.03 | 0.08 | 0.0639 | <.001*** |

*Note.*  CI = confidence interval; *LL* = lower limit; *UL* = upper limit. * *p* < .05, ** *p* < .01, *** *p* < .001.

## Table 41. Study 2 Inferential Statistics for American/Foreign Ratings by Condition and Political Orientation

| Effect | Estimate | *t* | *SE* | 95% CI | | η²ₚ | *p* |
| --- | --- | --- | --- | --- | --- | --- | --- |
|  |  |  |  | *LL* | *UL* |  |  |
| Fixed effects |  |  |  |  |  |  |  |
| Intercept | 4.25 | 102.41 | 0.04 | 4.17 | 4.33 | 0.948 | <.001*** |
| Black vs. Not Black | 0.01 | 0.14 | 0.05 | -0.09 | 0.10 | <.001 | 0.891 |
| South Asian vs. Not South  Asian | -0.03 | -0.67 | 0.05 | -0.12 | 0.06 | 0.001 | 0.505 |
| Black X South Asian | -0.05 | -0.99 | 0.05 | -0.14 | 0.05 | 0.002 | 0.321 |
| Harris vs. Other | 0.07 | 3.38 | 0.02 | 0.03 | 0.11 | 0.019 | 0.001** |
| Political Orientation (PO) | -0.25 | -11.01 | 0.02 | -0.30 | -0.21 | 0.173 | <.001*** |
| Black vs. Not Black X PO | -0.01 | -0.26 | 0.03 | -0.06 | 0.04 | <.001 | 0.792 |
| South Asian vs. Not South  Asian X PO | 0.001 | 0.15 | 0.03 | -0.05 | 0.06 | <.001 | 0.879 |
| Black X South Asian X PO | -0.012 | -0.46 | 0.03 | -0.06 | 0.04 | <.001 | 0.646 |
| Harris vs. Other X PO | 0.05 | 4.12 | 0.01 | 0.02 | 0.07 | 0.028 | <.001*** |

*Note.*  CI = confidence interval; *LL* = lower limit; *UL* = upper limit. * *p* < .05, ** *p* < .01, *** *p* < .001.

## Table 42. Study 2 Preregistered Contrast Codes

|  | Condition | | | | |
| --- | --- | --- | --- | --- | --- |
| Contrast | None | Both | Black | South Asian | Other |
| Both + Black vs. South Asian | 0 | -1 | -1 | 2 | 0 |
| Both + South Asian vs. Black | 0 | -1 | 2 | -1 | 0 |
| Both vs. Black | 0 | -1 | 1 | 0 | 0 |
| Both vs. South Asian | 0 | -1 | 0 | 1 | 0 |
| Black vs. South Asian | 0 | 0 | 0 | 1 | 0 |
| All vs. Other | 0 | -1 | -1 | -1 | 3 |
| All vs. None | 3 | -1 | -1 | -1 | 0 |
| None vs. Other | 0 | 0 | 0 | 0 | 1 |

## Table 43. Study 2 Inferential Statistics for Preregistered Contrasts on Trait Ratings by Condition and Political Orientation

| Effect | Estimate | *t* | *SE* | 95% CI | | η²ₚ | *p* |
| --- | --- | --- | --- | --- | --- | --- | --- |
|  |  |  |  | *LL* | *UL* |  |  |
| Fixed effects |  |  |  |  |  |  |  |
| Intercept | 3.64 | 96.39 | 0.04 | 3.57 | 3.72 | 0.941 | <.001*** |
| Both + Black vs. South Asian | 0.06 | 1.63 | 0.04 | -0.1 | 0.12 | 0.005 | 0.104 |
| Both + South Asian vs. Black | -0.03 | -0.90 | 0.03 | -0.10 | 0.04 | 0.001 | 0.368 |
| Both vs. Black | -0.01 | -0.09 | 0.06 | -0.01 | 0.12 | <.001 | 0.104 |
| Both vs. South Asian | 0.08 | 1.37 | 0.06 | -0.04 | 0.20 | 0.003 | 0.173 |
| Black vs. South Asian | 0.18 | 1.46 | 0.12 | -0.06 | 0.41 | 0.004 | 0.144 |
| All vs. Other | 0.17 | 6.74 | 0.03 | 0.12 | 0.21 | 0.073 | <.001*** |
| All vs. None  None vs. Other | 0.03  0.55 | 1.15  4.60 | 0.02  0.12 | -0.02  0.32 | 0.08  0.79 | 0.002  0.035 | 0.253  <.001*** |
| Political Orientation (PO) | -0.39 | -18.73 | 0.02 | -0.43 | -0.35 | 0.378 | <.001*** |
| Both + Black vs. South Asian X PO | 0.02 | 1.18 | 0.02 | -0.02 | 0.06 | 0.002 | 0.238 |
| Both + South Asian vs. Black X PO | -0.003 | -0.16 | 0.02 | -0.04 | 0.04 | <.001 | 0.871 |
| Both vs. Black X PO | 0.02 | 0.03 | 0.51 | -0.05 | 0.08 | <.001 | 0.614 |
| Both vs. South Asian X PO | 0.04 | 1.28 | 0.03 | -0.04 | 0.04 | 0.003 | 0.871 |
| Black vs. South Asian X PO | 0.05 | 0.78 | 0.07 | -0.08 | 0.19 | 0.001 | 0.438 |
| All vs. Other X PO | 0.09 | 6.70 | 0.01 | 0.06 | 0.12 | 0.072 | <.001*** |
| All vs. None X PO  None vs. Other X PO | 0.02  0.27 | 1.61  4.17 | 0.01  0.07 | -0.01  0.14 | 0.05  0.40 | 0.004  0.029 | 0.108  <.001*** |

*Note.*  CI = confidence interval; *LL* = lower limit; *UL* = upper limit. * *p* < .05, ** *p* < .01, *** *p* < .001.

## Table 44. Study 2 Inferential Statistics for Preregistered Contrasts on Thermometer Ratings by Condition and Political Orientation

| Effect | Estimate | *t* | *SE* | 95% CI | | η²ₚ | *p* |
| --- | --- | --- | --- | --- | --- | --- | --- |
|  |  |  |  | *LL* | *UL* |  |  |
| Fixed effects |  |  |  |  |  |  |  |
| Intercept | 56.40 | 50.84 | 1.11 | 54.22 | 58.58 | <.001 | <.001*** |
| Both + Black vs. South Asian | 1.91 | 1.88 | 1.02 | -0.09 | 3.91 | 0.006 | 0.061 |
| Both + South Asian vs. Black | -1.13 | -1.13 | 1.01 | -3.11 | 0.84 | 0.002 | 0.261 |
| Both vs. Black | -0.36 | -0.21 | 1.74 | -3.78 | 3.06 | <.001 | 0.837 |
| Both vs. South Asian | 2.68 | 1.52 | 1.76 | -0.78 | 6.14 | 0.004 | 0.13 |
| Black vs. South Asian | 6.08 | 1.73 | 3.51 | -0.81 | 12.97 | 0.005 | 0.084 |
| All vs. Other | 3.78 | 5.25 | 0.72 | 2.36 | 5.19 | 0.045 | <.001*** |
| All vs. None  None vs. Other | 0.66  12.49 | 0.92  3.55 | 0.71  3.51 | -0.75  5.59 | 2.06  19.39 | 0.001  0.021 | 0.358 <.001*** |
| Political Orientation (PO) | -11.02 | -17.94 | 0.61 | -12.22 | -9.81 | 0.358 | <.001*** |
| Both + Black vs. South Asian X  PO | 0.07 | 0.12 | 0.57 | -1.06 | 1.19 | <.001 | 0.907 |
| Both + South Asian vs. Black X  PO | -0.11 | -0.20 | 0.57 | -1.23 | 1.00 | <.001 | 0.841 |
| Both vs. Black X PO | -0.16 | -0.17 | 0.97 | -2.07 | 1.75 | <.001 | 0.869 |
| Both vs. South Asian X PO | 0.02 | 0.02 | 0.98 | -1.91 | 1.95 | <.001 | 0.983 |
| Black vs. South Asian X PO | 0.36 | 0.18 | 1.98 | -3.53 | 4.25 | <.001 | 0.855 |
| All vs. Other X PO | 2.28 | 5.83 | 0.39 | 1.51 | 3.05 | 0.055 | <.001*** |
| All vs. None X PO  None vs. Other X PO | 0.44  7.35 | 1.13  3.85 | 0.39  1.91 | -0.33  3.60 | 1.22  11.10 | 0.002  0.025 | 0.260  <.001*** |

*Note.*  CI = confidence interval; *LL* = lower limit; *UL* = upper limit. * *p* < .05, ** *p* < .01, *** *p* < .001.

## Table 45. Study 2 Inferential Statistics for Preregistered Contrasts on Political Abilities by Condition and Political Orientation

| Effect | Estimate | *t* | *SE* | 95% CI | | η²ₚ | *p* |
| --- | --- | --- | --- | --- | --- | --- | --- |
|  |  |  |  | *LL* | *UL* |  |  |
| Fixed effects |  |  |  |  |  |  |  |
| Intercept | 3.51 | 83.24 | 0.04 | 3.42 | 3.59 | 0.923 | <.001*** |
| Both + Black vs. South Asian | 0.05 | 1.30 | 0.04 | -0.03 | 0.13 | 0.003 | 0.195 |
| Both + South Asian vs. Black | -0.04 | -1.16 | 0.04 | -0.12 | 0.03 | 0.002 | 0.246 |
| Both vs. Black | -0.04 | -0.59 | 0.07 | -0.17 | 0.09 | 0.001 | 0.558 |
| Both vs. South Asian | 0.06 | 0.83 | 0.07 | -0.08 | 0.19 | 0.001 | 0.405 |
| Black vs. South Asian | 0.19 | 1.42 | 0.13 | -0.07 | 0.45 | 0.003 | 0.156 |
| All vs. Other | 0.24 | 8.91 | 0.03 | 0.19 | 0.30 | 0.121 | <.001*** |
| All vs. None  None vs. Other | 0.04  0.82 | 1.45  6.13 | 0.03  0.13 | -0.01  0.56 | 0.09  1.08 | 0.004  0.061 | 0.149  <.001*** |
| Political Orientation (PO) | -0.43 | -18.58 | 0.02 | -0.48 | -0.39 | 0.374 | <.001*** |
| Both + Black vs. South Asian  X PO | 0.01 | 0.55 | 0.02 | -0.03 | 0.06 | 0.001 | 0.584 |
| Both + South Asian vs. Black X  PO | -0.01 | -0.34 | 0.02 | -0.05 | 0.04 | <.001 | 0.735 |
| Both vs. Black X PO | -0.003 | -0.07 | 0.04 | -0.08 | 0.07 | <.001 | 0.941 |
| Both vs. South Asian X PO | 0.02 | 0.44 | 0.04 | -0.06 | 0.09 | <.001 | 0.659 |
| Black vs. South Asian X PO | 0.04 | 0.51 | 0.08 | -0.11 | 0.19 | <.001 | 0.610 |
| All vs. Other X PO | 0.10 | 7.00 | 0.02 | 0.08 | 0.13 | 0.078 | <.001*** |
| All vs. None X PO  None vs. Other X PO | 0.02  0.34 | 1.35  4.63 | 0.02  0.07 | -0.01  0.19 | 0.05  0.48 | 0.003  0.036 | 0.176  <.001*** |

*Note.*  CI = confidence interval; *LL* = lower limit; *UL* = upper limit. * *p* < .05, ** *p* < .01, *** *p* < .001.

## Table 46. Study 2 Inferential Statistics for Preregistered Contrasts on Superiority/Inferiority Ratings by Condition and Political Orientation

| Effect | Estimate | *t* | *SE* | 95% CI | | η²ₚ | *p* |
| --- | --- | --- | --- | --- | --- | --- | --- |
|  |  |  |  | *LL* | *UL* |  |  |
| Fixed effects |  |  |  |  |  |  |  |
| Intercept | 3.28 | 79.09 | 0.04 | 3.19 | 3.36 | 0.915 | <.001*** |
| Both + Black vs. South Asian | 0.06 | 1.45 | 0.04 | -0.02 | 0.13 | 0.004 | 0.149 |
| Both + South Asian vs. Black | -0.07 | -1.75 | 0.04 | -0.14 | 0.01 | 0.005 | 0.081 |
| Both vs. Black | -0.08 | -1.18 | 0.07 | -0.20 | 0.05 | 0.002 | 0.240 |
| Both vs. South Asian | 0.04 | 0.67 | 0.07 | -0.09 | 0.17 | 0.001 | 0.502 |
| Black vs. South Asian | 0.24 | 1.84 | 0.13 | -0.02 | 0.50 | 0.006 | 0.066 |
| All vs. Other | 0.12 | 4.48 | 0.03 | 0.07 | 0.17 | 0.034 | <.001*** |
| All vs. None  None vs. Other | 0.02  0.39 | 0.86  2.96 | 0.03  0.13 | -0.03  0.13 | 0.08  0.65 | 0.001  0.02 | 0.382  0.003** |
| Political Orientation (PO) | -0.31 | -13.53 | 0.02 | -0.20 | 0.05 | 0.241 | 0.240 |
| Both + Black vs. South Asian X  PO | 0.02 | 0.82 | 0.02 | -0.02 | 0.06 | 0.001 | 0.413 |
| Both + South Asian vs. Black X  PO | 0.02 | 0.77 | 0.02 | -0.03 | 0.06 | 0.001 | 0.439 |
| Both vs. Black X PO | 0.05 | 1.38 | 0.04 | -0.02 | 0.12 | 0.003 | 0.167 |
| Both vs. South Asian X PO | 0.05 | 1.40 | 0.04 | -0.02 | 0.12 | 0.003 | 0.162 |
| Black vs. South Asian X PO | 0.002 | 0.03 | 0.07 | -0.14 | 0.15 | <.001 | 0.976 |
| All vs. Other X PO | 0.08 | 5.28 | 0.02 | 0.05 | 0.11 | 0.046 | <.001*** |
| All vs. None X PO  None vs. Other X PO | 0.04  0.17 | 2.35  2.39 | 0.02  0.07 | 0.01  0.03 | 0.06  0.31 | 0.009  0.010 | 0.019*  0.017 |

*Note.*  CI = confidence interval; *LL* = lower limit; *UL* = upper limit. * *p* < .05, ** *p* < .01, *** *p* < .001.

## Table 47. Study 2 Inferential Statistics for Preregistered Contrasts on American/Foreign Ratings by Condition and Political Orientation

| Effect | Estimate | *t* | *SE* | 95% CI | | η²ₚ | *p* |
| --- | --- | --- | --- | --- | --- | --- | --- |
|  |  |  |  | *LL* | *UL* |  |  |
| Fixed effects |  |  |  |  |  |  |  |
| Intercept | 4.25 | 102.41 | 0.04 | 4.17 | 4.33 | 0.948 | <.001*** |
| Both + Black vs. South Asian | 0.001 | 0.02 | 0.04 | -0.07 | 0.08 | <.001 | 0.983 |
| Both + South Asian vs. Black | 0.04 | 1.01 | 0.04 | -0.4 | 0.11 | 0.002 | 0.313 |
| Both vs. Black | 0.08 | 1.18 | 0.07 | -0.05 | 0.21 | 0.002 | 0.238 |
| Both vs. South Asian | 0.04 | 0.60 | 0.07 | -0.09 | 0.17 | 0.001 | 0.547 |
| Black vs. South Asian | -0.07 | -0.57 | 0.13 | -0.33 | 0.18 | 0.001 | 0.571 |
| All vs. Other | 0.09 | 3.21 | 0.03 | 0.03 | 0.14 | 0.017 | 0.001** |
| All vs. None  None vs. Other | -0.01  0.37 | -0.27  2.85 | -0.06  0.13 | 0.05  0.12 | 0.0  0.63 | <.001  0.014 | 0.789  0.005 |
| Political Orientation (PO) | -0.25 | -11.01 | 0.02 | -0.30 | -0.21 | 0.173 | <.001*** |
| Both + Black vs. South Asian X  PO | 0.01 | 0.46 | 0.02 | -0.03 | 0.05 | <.001 | 0.647 |
| Both + South Asian vs. Black X  PO | -0.001 | -0.04 | 0.02 | -0.04 | 0.04 | <.001 | 0.965 |
| Both vs. Black X PO | 0.01 | 0.22 | 0.04 | -0.06 | 0.08 | <.001 | 0.828 |
| Both vs. South Asian X PO | 0.02 | 0.51 | 0.04 | -0.05 | 0.09 | <.001 | 0.612 |
| Black vs. South Asian X PO | 0.02 | 0.29 | 0.07 | -0.12 | 0.17 | <.001 | 0.772 |
| All vs. Other X PO | 0.06 | 3.92 | 0.02 | 0.03 | 0.09 | 0.026 | <.001*** |
| All vs. None X PO  None vs. Other X PO | -0.003  0.24 | -0.20  3.39 | 0.02  0.07 | -0.03  0.10 | 0.03  0.38 | <.001  0.019 | 0.839  0.001 |

*Note.*  CI = confidence interval; *LL* = lower limit; *UL* = upper limit. * *p* < .05, ** *p* < .01, *** *p* < .001.

## Table 48. Study 2 Inferential Statistics for Performance as a Political Leader by Condition and Political Orientation

| Effect | Estimate | *t* | *SE* | 95% CI | | η²ₚ | *p* |
| --- | --- | --- | --- | --- | --- | --- | --- |
|  |  |  |  | *LL* | *UL* |  |  |
| Fixed effects |  |  |  |  |  |  |  |
| Intercept | 3.08 | 63.69 | 0.05 | 2.98 | 3.17 | 0.875 | <.001*** |
| Black vs. Not Black | -0.06 | -1.10 | 0.05 | -0.17 | 0.05 | 0.002 | 0.274 |
| South Asian vs. Not South Asian | 0.002 | 0.03 | 0.05 | -0.10 | 0.11 | <.001 | 0.977 |
| Black X South Asian | 0.04 | 0.76 | 0.05 | -0.07 | 0.15 | 0.001 | 0.446 |
| Harris vs Other | 0.03 | 1.42 | 0.02 | -0.01 | 0.08 | 0.003 | 0.158 |
| Political Orientation (PO) | -0.56 | -21.04 | 0.03 | -0.62 | -0.51 | 0.434 | <.001*** |
| Black vs. Not Black X PO | -0.1 | -0.33 | 0.03 | -0.07 | 0.05 | <.001 | 0.740 |
| South Asian vs. Not South Asian  X PO | -0.02 | -0.75 | 0.03 | -0.08 | 0.04 | 0.001 | 0.457 |
| Black X South Asian X PO | 0.03 | 0.95 | 0.03 | -0.03 | 0.09 | 0.002 | 0.343 |
| Harris vs. Other X PO | -0.01 | -0.66 | 0.01 | -0.04 | 0.02 | 0.001 | 0.508 |

*Note.*  CI = confidence interval; *LL* = lower limit; *UL* = upper limit. * *p* < .05, ** *p* < .01, *** *p* < .001.

## Table 49. Study 2 Inferential Statistics for Perceptions of Harris as Black by Condition and Political Orientation

| Effect | Estimate | *t* | *SE* | 95% CI | | η²ₚ | *p* |
| --- | --- | --- | --- | --- | --- | --- | --- |
|  |  |  |  | *LL* | *UL* |  |  |
| Fixed effects |  |  |  |  |  |  |  |
| Intercept | 3.34 | 71.68 | 0.05 | 3.25 | 3.43 | 0.899 | <.001*** |
| Black vs. Not Black | 0.02 | 0.31 | 0.05 | -0.09 | 0.19 | <.001 | 0.757 |
| South Asian vs. Not South Asian | -0.003 | -0.06 | 0.05 | -0.11 | 0.10 | <.001 | 0.950 |
| Black X South Asian | 0.06 | 1.13 | 0.05 | -0.04 | 0.16 | 0.002 | 0.261 |
| Harris vs Other | 0.04 | 1.74 | 0.02 | -0.01 | 0.09 | 0.005 | 0.082 |
| Political Orientation (PO) | -0.31 | -12.16 | 0.03 | -0.36 | -0.26 | 0.204 | <.001*** |
| Black vs. Not Black X PO | -0.06 | -1.90 | 0.03 | -0.11 | 0.002 | 0.006 | 0.058 |
| South Asian vs. Not South Asian  X PO | 0.02 | 0.74 | 0.03 | -0.04 | 0.08 | 0.001 | 0.460 |
| Black X South Asian X PO | -0.04 | -1.46 | 0.03 | -0.10 | 0.02 | 0.004 | 0.155 |
| Harris vs. Other X PO | -0.01 | -0.36 | 0.01 | -0.03 | 0.02 | <.001 | 0.719 |

*Note.*  CI = confidence interval; *LL* = lower limit; *UL* = upper limit. * *p* < .05, ** *p* < .01, *** *p* < .001.

## Table 50. Study 2 Inferential Statistics for Perception of Harris as South Asian by Condition and Political Orientation

| Effect | Estimate | *t* | *SE* | 95% CI | | η²ₚ | *p* |
| --- | --- | --- | --- | --- | --- | --- | --- |
|  |  |  |  | *LL* | *UL* |  |  |
| Fixed effects |  |  |  |  |  |  |  |
| Intercept | 3.43 | 74.97 | 0.05 | 3.34 | 3.52 | 0.907 | <.001*** |
| Black vs. Not Black | 0.01 | 0.20 | 0.05 | -0.09 | 0.11 | <.001 | 0.844 |
| South Asian vs. Not South Asian | 0.001 | 0.01 | 0.05 | -0.10 | 0.10 | <.001 | 0.989 |
| Black X South Asian | -0.05 | -1.01 | 0.05 | -0.15 | 0.05 | 0.002 | 0.313 |
| Harris vs. Other | -0.03 | -1.39 | 0.02 | -0.08 | 0.01 | 0.003 | 0.164 |
| Political Orientation (PO) | -0.22 | -8.47 | 0.03 | -0.26 | -0.17 | 0.110 | <.001*** |
| Black vs. Not Black X PO | 0.03 | 1.09 | 0.03 | -0.03 | 0.09 | 0.002 | 0.275 |
| South Asian vs. Not South Asian  X PO | -0.03 | -1.15 | 0.03 | -0.09 | 0.02 | 0.002 | 0.252 |
| Black X South Asian X PO | -0.08 | -2.74 | 0.03 | -0.13 | -0.02 | 0.013 | 0.006** |
| Harris vs. Other X PO | 0.01 | 1.05 | 0.01 | -0.01 | 0.04 | 0.002 | 0.295 |

*Note.*  CI = confidence interval; *LL* = lower limit; *UL* = upper limit. * *p* < .05, ** *p* < .01, *** *p* < .001.

## Table 51. Study 2 Inferential Statistics for Harris’s Perceptions of Herself as Black by Condition and Political Orientation

| Effect | Estimate | *t* | *SE* | 95% CI | | η²ₚ | *p* |
| --- | --- | --- | --- | --- | --- | --- | --- |
|  |  |  |  | *LL* | *UL* |  |  |
| Fixed effects |  |  |  |  |  |  |  |
| Intercept | 3.64 | 80.21 | 0.05 | 3.55 | 3.73 | 0.918 | <.001*** |
| Black vs. Not Black | 0.08 | 1.64 | 0.05 | -0.02 | 0.18 | 0.005 | 0.102 |
| South Asian vs. Not South Asian | -0.03 | -0.61 | 0.05 | -0.13 | 0.07 | 0.001 | 0.541 |
| Black X South Asian | 0.14 | 2.72 | 0.05 | 0.04 | 0.24 | 0.013 | 0.007** |
| Other vs. Harris | 0.05 | 2.32 | 0.02 | 0.01 | 0.10 | 0.009 | 0.021* |
| Political Orientation (PO) | -0.16 | -6.19 | 0.03 | -0.21 | -0.11 | 0.062 | <.001*** |
| Black vs. Not Black X PO | -0.06 | -2.10 | 0.03 | -0.12 | -0.004 | 0.008 | 0.036* |
| South Asian vs. Not South Asian  X PO | -0.04 | -1.26 | 0.03 | -0.09 | 0.02 | 0.003 | 0.207 |
| Black X South Asian X PO | 0.05 | 1.68 | 0.03 | -0.01 | 0.10 | 0.005 | 0.094 |
| Harris vs. Other X PO | -0.01 | -0.65 | 0.01 | -0.03 | 0.02 | 0.001 | 0.515 |

*Note.*  CI = confidence interval; *LL* = lower limit; *UL* = upper limit. * *p* < .05, ** *p* < .01, *** *p* < .001.

## Table 52. Study 2 Inferential Statistics for Harris’s Perceptions of Herself as South Asian by Condition and Political Orientation

| Effect | Estimate | *t* | *SE* | 95% CI | | η²ₚ | *p* |
| --- | --- | --- | --- | --- | --- | --- | --- |
|  |  |  |  | *LL* | *UL* |  |  |
| Fixed effects |  |  |  |  |  |  |  |
| Intercept | 3.44 | 75.72 | 0.05 | 3.35 | 3.52 | 0.909 | <.001*** |
| Black vs. Not Black | 0.01 | 0.15 | 0.05 | -0.09 | 0.11 | <.001 | 0.881 |
| South Asian vs. Not South Asian | 0.04 | 0.82 | 0.05 | -0.06 | 0.14 | 0.001 | 0.414 |
| Black X South Asian | -0.04 | -0.74 | 0.05 | -0.14 | 0.06 | 0.001 | 0.458 |
| Other vs. Harris | -0.01 | -0.39 | 0.02 | -0.05 | 0.04 | <.001 | 0.696 |
| Political Orientation (PO) | -0.22 | -8.81 | 0.03 | -0.27 | -0.17 | 0.119 | <.001*** |
| Black vs. Not Black X PO | -0.004 | -0.13 | 0.03 | -0.06 | 0.05 | <.001 | 0.893 |
| South Asian vs. Not South Asian  X PO | -0.04 | -1.45 | 0.03 | -0.10 | 0.02 | 0.004 | 0.148 |
| Black X South Asian X PO | -0.04 | -1.56 | 0.03 | -0.10 | 0.01 | 0.004 | 0.120 |
| Harris vs. Other X PO | 0.001 | 0.07 | 0.01 | -0.02 | 0.03 | <.001 | 0.945 |

*Note.*  CI = confidence interval; *LL* = lower limit; *UL* = upper limit. * *p* < .05, ** *p* < .01, *** *p* < .001.

## Table 53. Study 2 Inferential Statistics for Other’s Perceptions of Black Americans as Superior/Inferior by Condition and Political Orientation

| Effect | Estimate | *t* | *SE* | 95% CI | | η²ₚ | *p* |
| --- | --- | --- | --- | --- | --- | --- | --- |
|  |  |  |  | *LL* | *UL* |  |  |
| Fixed effects |  |  |  |  |  |  |  |
| Intercept | 3.10 | 112.77 | 0.03 | 3.05 | 3.15 | 0.957 | <.001*** |
| Black vs. Not Black | <.001 | -0.01 | 0.03 | -0.06 | 0.06 | <.001 | 0.992 |
| South Asian vs. Not South Asian | -0.05 | -1.62 | 0.03 | -0.11 | 0.01 | 0.005 | 0.105 |
| Black X South Asian | -0.04 | -1.17 | 0.03 | -0.10 | 0.02 | 0.002 | 0.243 |
| Other vs. Harris | -0.002 | -0.16 | 0.01 | -0.03 | 0.03 | <.001 | 0.872 |
| Political Orientation (PO) | -0.02 | -0.96 | 0.02 | -0.04 | 0.02 | 0.002 | 0.339 |
| Black vs. Not Black X PO | -0.03 | -1.50 | 0.02 | -0.06 | 0.01 | 0.004 | 0.133 |
| South Asian vs. Not South Asian  X PO | 0.001 | 0.06 | 0.02 | -0.03 | 0.04 | <.001 | 0.950 |
| Black X South Asian X PO | -0.002 | -0.09 | 0.02 | -0.04 | 0.03 | <.001 | 0.926 |
| Harris vs. Other X PO | -0.004 | -0.48 | 0.01 | -0.02 | 0.01 | <.001 | 0.630 |

*Note.*  CI = confidence interval; *LL* = lower limit; *UL* = upper limit. * *p* < .05, ** *p* < .01, *** *p* < .001.

## Table 54. Study 2 Inferential Statistics for Other’s Perceptions of Black Americans as American/Foreign by Condition and Political Orientation

| Effect | Estimate | *t* | *SE* | 95% CI | | η²ₚ | *p* |
| --- | --- | --- | --- | --- | --- | --- | --- |
|  |  |  |  | *LL* | *UL* |  |  |
| Fixed effects |  |  |  |  |  |  |  |
| Intercept | 1.69 | 43.12 | 0.04 | 1.62 | 1.77 | 0.763 | <.001*** |
| Black vs. Not Black | 0.02 | 0.38 | 0.04 | -0.07 | 0.10 | <.001 | 0.705 |
| South Asian vs. Not South Asian | -0.04 | -1.00 | 0.04 | -0.13 | 0.04 | 0.002 | 0.319 |
| Black X South Asian | 0.01 | 0.17 | 0.04 | -0.08 | 0.09 | <.001 | 0.868 |
| Other vs. Harris | 0.003 | 0.14 | 0.02 | -0.04 | 0.04 | <.001 | 0.889 |
| Political Orientation (PO) | 0.07 | 3.38 | 0.02 | 0.03 | 0.12 | 0.019 | 0.001** |
| Black vs. Not Black X PO | -0.01 | -0.32 | 0.02 | -0.06 | 0.04 | <.001 | 0.751 |
| South Asian vs. Not South Asian  X PO | -0.01 | -0.31 | 0.02 | -0.06 | 0.04 | <.001 | 0.755 |
| Black X South Asian X PO | -0.02 | -0.72 | 0.02 | -0.07 | 0.03 | 0.001 | 0.475 |
| Harris vs. Other X PO | 0.02 | 1.92 | 0.01 | <.001 | 0.04 | 0.006 | 0.055 |

*Note.*  CI = confidence interval; *LL* = lower limit; *UL* = upper limit. * *p* < .05, ** *p* < .01, *** *p* < .001.

## Table 55. Study 2 Inferential Statistics for Other’s Perceptions of South Asian Americans as Superior/Inferior by Condition and Political Orientation

| Effect | Estimate | *t* | *SE* | 95% CI | | η²ₚ | *p* |
| --- | --- | --- | --- | --- | --- | --- | --- |
|  |  |  |  | *LL* | *UL* |  |  |
| Fixed effects |  |  |  |  |  |  |  |
| Intercept | 3.18 | 135.55 | 0.02 | 3.13 | 3.23 | 0.970 | <.001*** |
| Black vs. Not Black | -0.04 | -1.42 | 0.03 | -0.09 | 0.01 | 0.003 | 0.157 |
| South Asian vs. Not South Asian | -0.01 | -0.27 | 0.03 | -0.06 | 0.04 | <.001 | 0.787 |
| Black X South Asian | -0.02 | -0.66 | 0.03 | -0.07 | 0.03 | 0.001 | 0.508 |
| Other vs. Harris | -0.01 | -0.78 | 0.01 | -0.03 | 0.01 | 0.001 | 0.439 |
| Political Orientation (PO) | 0.01 | 0.52 | 0.01 | -0.02 | 0.03 | <.001 | 0.605 |
| Black vs. Not Black X PO | -0.02 | -1.09 | 0.02 | -0.04 | 0.01 | 0.002 | 0.278 |
| South Asian vs. Not South Asian  X PO | -0.01 | -0.84 | 0.02 | -0.04 | 0.02 | 0.001 | 0.401 |
| Black X South Asian X PO | -0.001 | -0.04 | 0.02 | -0.03 | 0.03 | <.001 | 0.968 |
| Harris vs. Other X PO | 0.01 | 0.87 | 0.01 | -0.01 | 0.02 | 0.001 | 0.387 |

*Note.*  CI = confidence interval; *LL* = lower limit; *UL* = upper limit. * *p* < .05, ** *p* < .01, *** *p* < .001.

## Table 56. Study 2 Inferential Statistics for Other’s Perceptions of South Asian as American/Foreign by Condition and Political Orientation

| Effect | Estimate | *t* | *SE* | 95% CI | | η²ₚ | *p* |
| --- | --- | --- | --- | --- | --- | --- | --- |
|  |  |  |  | *LL* | *UL* |  |  |
| Fixed effects |  |  |  |  |  |  |  |
| Intercept | 2.29 | 45.10 | 0.05 | 2.19 | 2.39 | 0.779 | <.001*** |
| Black vs. Not Black | -0.04 | -0.69 | 0.06 | -0.15 | 0.07 | 0.001 | 0.494 |
| South Asian vs. Not South Asian | -0.06 | -1.10 | 0.06 | -0.17 | 0.05 | 0.002 | 0.274 |
| Black X South Asian | 0.02 | 0.35 | 0.06 | -0.09 | 0.13 | <.001 | 0.727 |
| Other vs. Harris | 0.02 | 0.61 | 0.03 | -0.04 | 0.07 | 0.001 | 0.544 |
| Political Orientation (PO) | 0.20 | 7.10 | 0.03 | 0.14 | 0.25 | 0.080 | <.001*** |
| Black vs. Not Black X PO | 0.03 | 0.82 | 0.03 | -0.04 | 0.09 | 0.001 | 0.413 |
| South Asian vs. Not South Asian  X PO | -0.01 | -0.25 | 0.03 | -0.07 | 0.05 | <.001 | 0.800 |
| Black X South Asian X PO | -0.02 | -0.72 | 0.03 | -0.09 | 0.04 | 0.001 | 0.475 |
| Harris vs. Other X PO | 0.02 | 1.33 | 0.01 | -0.01 | 0.05 | 0.003 | 0.184 |

*Note.*  CI = confidence interval; *LL* = lower limit; *UL* = upper limit. * *p* < .05, ** *p* < .01, *** *p* < .001.

## Table 57. Study 2 Inferential Statistics for Perceptions of Afrocentricity by Condition and Political Orientation

| Effect | Estimate | *t* | *SE* | 95% CI | | η²ₚ | *p* |
| --- | --- | --- | --- | --- | --- | --- | --- |
|  |  |  |  | *LL* | *UL* |  |  |
| Fixed effects |  |  |  |  |  |  |  |
| Intercept | 49.72 | 48.15 | 1.03 | 47.693 | 51.75 | 0.800 | <.001*** |
| Black vs. Not Black | -0.48 | -0.42 | 1.15 | -2.75 | 1.78 | <.001 | 0.675 |
| South Asian vs. Not South Asian | 1.91 | 1.67 | 1.15 | -0.36 | 4.17 | 0.005 | 0.099 |
| Black X South Asian | 2.10 | 1.82 | 1.15 | -0.16 | 4.36 | 0.006 | 0.069 |
| Other vs. Harris | 0.45 | 0.87 | 0.52 | -0.57 | 1.47 | 0.001 | 0.387 |
| Political Orientation (PO) | -3.19 | -5.59 | 0.57 | -4.32 | -2.07 | 0.051 | <.001*** |
| Black vs. Not Black X PO | -0.79 | -1.23 | 0.64 | -2.05 | 0.47 | 0.003 | 0.221 |
| South Asian vs. Not South Asian  X PO | -0.12 | -0.03 | 0.64 | -1.278 | 1.25 | <.001 | 0.980 |
| Black X South Asian X PO | -0.99 | -1.55 | 0.64 | -2.26 | 0.27 | 0.004 | 0.122 |
| Harris vs. Other X PO | -0.25 | -0.90 | 0.28 | -0.81 | 0.30 | 0.001 | 0.368 |

*Note.*  CI = confidence interval; *LL* = lower limit; *UL* = upper limit. * *p* < .05, ** *p* < .01, *** *p* < .001.

## Table 58. Study 2 Inferential Statistics for Perceptions of South Asian Features by Condition and Political Orientation

| Effect | Estimate | *t* | *SE* | 95% CI | | η²ₚ | *p* |
| --- | --- | --- | --- | --- | --- | --- | --- |
|  |  |  |  | *LL* | *UL* |  |  |
| Fixed effects  Intercept | 46.08 | 46.58 | 0.99 | 44.14 | 48.03 | 0.790 | <.001*** |
| Black vs. Not Black | 0.02 | 0.02 | 1.10 | -2.15 | 2.19 | <.001 | 0.987 |
| South Asian vs. Not South Asian | 0.06 | 0.05 | 1.10 | -2.11 | 2.23 | <.001 | 0.958 |
| Black X South Asian | -1.84 | -1.67 | 1.10 | -4.01 | 0.33 | 0.005 | 0.096 |
| Other vs. Harris | -0.34 | -0.68 | 0.50 | -1.32 | 0.64 | 0.001 | 0.497 |
| Political Orientation (PO) | -0.93 | -1.69 | 0.55 | -2.00 | 0.15 | 0.005 | 0.091 |
| Black vs. Not Black X PO | 0.91 | 1.49 | 0.62 | -0.30 | 2.12 | 0.004 | 0.138 |
| South Asian vs. Not South Asian  X PO | -1.14 | -1.85 | 0.62 | -2.35 | 0.07 | 0.006 | 0.064 |
| Black X South Asian X PO | -1.34 | -2.17 | 0.62 | -2.54 | -0.13 | 0.008 | 0.030* |
| Harris vs. Other X PO | 0.09 | 0.35 | 0.27 | -0.44 | 0.62 | <.001 | 0.728 |

*Note.*  CI = confidence interval; *LL* = lower limit; *UL* = upper limit. * *p* < .05, ** *p* < .01, *** *p* < .001.

## Table 59. Study 2 Inferential Statistics for Traits by Condition and Gender

| Effect | Estimate | *t* | *SE* | 95% CI | | η²ₚ | *p* |
| --- | --- | --- | --- | --- | --- | --- | --- |
|  |  |  |  | *LL* | *UL* |  |  |
| Fixed effects |  |  |  |  |  |  |  |
| Intercept | 3.63 | 74.64 | 0.05 | 3.54 | 3.73 | 0.906 | <.001*** |
| Black vs Not Black | -0.09 | -1.67 | 0.05 | -0.20 | 0.02 | 0.005 | 0.095 |
| South Asian vs Not South Asian | 0.03 | 0.62 | 0.05 | -0.07 | 0.14 | 0.001 | 0.536 |
| Black X South Asian | 0.00 | 0.01 | 0.05 | -0.11 | 0.11 | <.001 | 0.996 |
| Harris vs. Other | 0.13 | 5.41 | 0.03 | 0.09 | 0.18 | 0.048 | <.001*** |
| Participant Gender (PG) | 0.21 | 2.15 | 0.10 | 0.02 | 0.40 | 0.008 | 0.032* |
| Black vs Not Black X PG | -0.09 | -0.80 | 0.11 | -0.30 | 0.13 | 0.001 | 0.424 |
| South Asian vs Not South Asian  X PG | 0.10 | 0.92 | 0.11 | -0.11 | 0.31 | 0.001 | 0.359 |
| Black X South Asian X PG | 0.16 | 1.46 | 0.11 | -0.06 | 0.37 | 0.004 | 0.145 |
| Harris vs. Other X PG | -0.05 | -1.10 | 0.05 | -0.15 | 0.04 | 0.002 | 0.273 |

*Note.*  CI = confidence interval; *LL* = lower limit; *UL* = upper limit. * *p* < .05, ** *p* < .01, *** *p* < .001.

## Table 60. Study 2 Inferential Statistics for Feeling Thermometer by Condition and Gender

| Effect | Estimate | *t* | *SE* | 95% CI | | η²ₚ | *p* |
| --- | --- | --- | --- | --- | --- | --- | --- |
|  |  |  |  | *LL* | *UL* |  |  |
| Fixed effects |  |  |  |  |  |  |  |
| Intercept | 56.31 | 40.10 | 1.40 | 53.55 | 59.07 | 0.736 | <.001*** |
| Black vs Not Black | -2.76 | -1.77 | 1.56 | -5.83 | 0.31 | 0.005 | 0.078 |
| South Asian vs Not South Asian | 1.41 | 0.90 | 1.56 | -1.66 | 4.48 | 0.001 | 0.366 |
| Black X South Asian | -0.24 | -0.15 | 1.56 | -3.31 | 2.83 | <.001 | 0.880 |
| Harris vs. Other | 3.16 | 4.44 | 0.71 | 1.76 | 4.56 | 0.033 | <.001*** |
| Participant Gender (PG) | 2.16 | 0.77 | 2.81 | -3.36 | 7.68 | 0.001 | 0.442 |
| Black vs Not Black X PG | -2.76 | -1.77 | 1.56 | -5.83 | 0.31 | 0.005 | 0.078 |
| South Asian vs Not South Asian  X PG | -0.34 | -0.11 | 3.13 | -6.48 | 5.80 | <.001 | 0.914 |
| Black X South Asian X PG | 4.54 | 1.45 | 3.13 | -1.60 | 10.68 | 0.004 | 0.147 |
| Harris vs. Other X PG | -1.69 | -1.19 | 1.42 | -4.49 | 1.11 | 0.002 | 0.236 |

*Note.*  CI = confidence interval; *LL* = lower limit; *UL* = upper limit. * *p* < .05, ** *p* < .01, *** *p* < .001.

## Table 61. Study 2 Inferential Statistics for Political Abilities by Condition and Gender

| Effect | Estimate | *t* | *SE* | 95% CI | | η²ₚ | *p* |
| --- | --- | --- | --- | --- | --- | --- | --- |
|  |  |  |  | *LL* | *UL* |  |  |
| Fixed effects |  |  |  |  |  |  |  |
| Intercept | 3.50 | 64.56 | 0.05 | 3.39 | 3.61 | 0.878 | <.001*** |
| Black vs Not Black | -0.10 | -1.63 | 0.06 | -0.22 | 0.02 | 0.005 | 0.104 |
| South Asian vs Not South Asian | 0.04 | 0.68 | 0.06 | -0.08 | 0.16 | 0.001 | 0.499 |
| Black X South Asian | 0.03 | 0.50 | 0.06 | -0.09 | 0.15 | <.001 | 0.615 |
| Harris vs. Other | 0.20 | 7.12 | 0.03 | 0.14 | 0.25 | 0.081 | <.001*** |
| Participant Gender (PG) | 0.17 | 1.55 | 0.11 | -0.05 | 0.38 | 0.004 | 0.122 |
| Black vs Not Black X PG | -0.09 | -0.75 | 0.12 | -0.33 | 0.15 | 0.001 | 0.452 |
| South Asian vs Not South Asian  X PG | -0.01 | -0.11 | 0.12 | -0.25 | 0.22 | <.001 | 0.910 |
| Black X South Asian X PG | 0.22 | 1.84 | 0.12 | -0.02 | 0.46 | 0.006 | 0.066 |
| Harris vs. Other X PG | -0.08 | -1.40 | 0.06 | -0.19 | 0.03 | 0.003 | 0.161 |

*Note.*  CI = confidence interval; *LL* = lower limit; *UL* = upper limit. * *p* < .05, ** *p* < .01, *** *p* < .001.

## Table 62. Study 2 Inferential Statistics for Superior/Inferior by Condition and Gender

| Effect | Estimate | *t* | *SE* | 95% CI | | η²ₚ | *p* |
| --- | --- | --- | --- | --- | --- | --- | --- |
|  |  |  |  | *LL* | *UL* |  |  |
| Fixed effects |  |  |  |  |  |  |  |
| Intercept | 3.27 | 67.81 | 0.05 | 3.18 | 3.37 | 0.888 | <.001*** |
| Black vs Not Black | -0.09 | -1.58 | 0.05 | -0.19 | 0.02 | 0.004 | 0.115 |
| South Asian vs Not South Asian | 0.07 | 1.22 | 0.05 | -0.04 | 0.17 | 0.003 | 0.224 |
| Black X South Asian | 0.03 | 0.62 | 0.05 | -0.07 | 0.14 | 0.001 | 0.538 |
| Harris vs. Other | 0.10 | 3.99 | 0.02 | 0.05 | 0.15 | 0.027 | <.001*** |
| Participant Gender (PG) | 0.12 | 1.28 | 0.10 | -0.07 | 0.31 | 0.003 | 0.203 |
| Black vs Not Black X PG | -0.06 | -0.59 | 0.11 | -0.27 | 0.15 | 0.001 | 0.558 |
| South Asian vs Not South Asian  X PG | 0.10 | 0.97 | 0.11 | -0.11 | 0.32 | 0.002 | 0.335 |
| Black X South Asian X PG | 0.16 | 1.49 | 0.11 | -0.05 | 0.37 | 0.004 | 0.137 |
| Harris vs. Other X PG | -0.05 | -1.09 | 0.05 | -0.15 | 0.04 | 0.002 | 0.275 |

*Note.*  CI = confidence interval; *LL* = lower limit; *UL* = upper limit. * *p* < .05, ** *p* < .01, *** *p* < .001.

## Table 63. Study 2 Inferential Statistics for American/Foreign by Condition and Gender

| Effect | Estimate | *t* | *SE* | 95% CI | | η²ₚ | *p* |
| --- | --- | --- | --- | --- | --- | --- | --- |
|  |  |  |  | *LL* | *UL* |  |  |
| Fixed effects |  |  |  |  |  |  |  |
| Intercept | 4.24 | 91.77 | 0.05 | 4.15 | 4.34 | 0.936 | <.001*** |
| Black vs Not Black | 0.01 | 0.12 | 0.05 | -0.10 | 0.11 | <.001 | 0.903 |
| South Asian vs Not South Asian | -0.01 | -0.14 | 0.05 | -0.11 | 0.09 | <.001 | 0.892 |
| Black X South Asian | -0.05 | -0.99 | 0.05 | -0.15 | 0.05 | 0.002 | 0.323 |
| Harris vs. Other | 0.08 | 3.19 | 0.02 | 0.03 | 0.12 | 0.017 | 0.002** |
| Participant Gender (PG) | 0.06 | 0.64 | 0.09 | -0.12 | 0.24 | 0.001 | 0.522 |
| Black vs Not Black X PG | 0.01 | 0.06 | 0.10 | -0.20 | 0.21 | <.001 | 0.950 |
| South Asian vs Not South Asian  X PG | <.001 | -0.003 | 0.10 | -0.20 | 0.20 | <.001 | 0.997 |
| Black X South Asian X PG | -0.02 | -0.19 | 0.10 | -0.22 | 0.18 | <.001 | 0.848 |
| Harris vs. Other X PG | -0.01 | -0.30 | 0.05 | -0.11 | 0.08 | <.001 | 0.762 |

*Note.*  CI = confidence interval; *LL* = lower limit; *UL* = upper limit. * *p* < .05, ** *p* < .01, *** *p* < .001.

## Table 64. Study 2 Inferential Statistics for Traits by Condition and Race

| Effect | Estimate | *t* | *SE* | 95% CI | | η²ₚ | *p* |
| --- | --- | --- | --- | --- | --- | --- | --- |
|  |  |  |  | *LL* | *UL* |  |  |
| Fixed effects |  |  |  |  |  |  |  |
| Intercept | 3.65 | 72.42 | 0.05 | 3.55 | 3.75 | 0.901 | <.001^***^ |
| Black vs Not Black | -0.10 | -1.85 | 0.06 | -0.21 | 0.01 | 0.006 | 0.065 |
| South Asian vs Not South Asian | 0.06 | 1.02 | 0.06 | -0.05 | 0.17 | 0.002 | 0.307 |
| Black X South Asian | -0.00 | -0.04 | 0.06 | -0.11 | 0.11 | 0.000 | 0.971 |
| Harris vs. Other | 0.13 | 5.41 | 0.03 | 0.09 | 0.19 | 0.047 | <.001^***^ |
| POC vs. Not POC | -0.11 | -1.11 | 0.10 | -0.31 | 0.09 | 0.002 | 0.270 |
| Other vs. Harris X POC vs. Not  POC | -0.01 | -0.27 | 0.05 | -0.11 | 0.09 | 0.000 | 0.789 |
| Black vs Not Black X POC vs.  Not POC | 0.13 | 1.12 | 0.11 | -0.10 | 0.35 | 0.002 | 0.265 |
| South Asian vs Not South Asian  X POC vs. Not POC | -0.10 | -0.90 | 0.11 | -0.32 | 0.12 | 0.001 | 0.370 |
| Black X South Asian X POC vs.  Not POC | -0.04 | -0.35 | 0.11 | -0.26 | 0.18 | 0.000 | 0.724 |

*Note.*  CI = confidence interval; *LL* = lower limit; *UL* = upper limit. * *p* < .05, ** *p* < .01, *** *p* < .001.

## Table 65. Study 2 Inferential Statistics for Feeling Thermometer by Condition and Race

| Effect | Estimate | *t* | *SE* | 95% CI | | η²ₚ | *p* |
| --- | --- | --- | --- | --- | --- | --- | --- |
|  |  |  |  | *LL* | *UL* |  |  |
| Fixed effects |  |  |  |  |  |  |  |
| Intercept | 56.43 | 38.97 | 1.45 | 53.59 | 59.28 | 0.724 | <.001^***^ |
| Black vs Not Black | -3.13 | -1.93 | 1.62 | -6.30 | 0.05 | 0.006 | 0.054 |
| South Asian vs Not South Asian | 1.79 | 1.11 | 1.62 | -1.38 | 4.97 | 0.002 | 0.268 |
| Black X South Asian | -0.32 | -0.20 | 1.62 | -3.50 | 2.85 | 0.000 | 0.842 |
| Harris vs. Other | 3.06 | 4.21 | 0.73 | 1.63 | 4.48 | 0.030 | <.001^***^ |
| POC vs. Not POC | -2.15 | -0.74 | 2.90 | -7.84 | 3.53 | 0.001 | 0.457 |
| Other vs. Harris X POC vs. Not  POC | 0.33 | 0.23 | 1.45 | -2.52 | 3.18 | 0.000 | 0.820 |
| Black vs Not Black X POC vs.  Not POC | 3.88 | 1.20 | 3.24 | -2.47 | 10.23 | 0.002 | 0.231 |
| South Asian vs Not South Asian  X POC vs. Not POC | -0.97 | -0.30 | 3.24 | -7.32 | 5.38 | 0.000 | 0.764 |
| Black X South Asian X POC vs.  Not POC | -0.89 | -0.27 | 3.24 | -7.24 | 5.47 | 0.000 | 0.785 |

*Note.*  CI = confidence interval; *LL* = lower limit; *UL* = upper limit. * *p* < .05, ** *p* < .01, *** *p* < .001.

## Table 66. Study 2 Inferential Statistics for Political Abilities by Condition and Race

| Effect | Estimate | *t* | *SE* | 95% CI | | η²ₚ | *p* |
| --- | --- | --- | --- | --- | --- | --- | --- |
|  |  |  |  | *LL* | *UL* |  |  |
| Fixed effects |  |  |  |  |  |  |  |
| Intercept | 3.51 | 62.66 | 0.06 | 3.40 | 3.62 | 0.872 | <.001^***^ |
| Black vs Not Black | -0.10 | -1.63 | 0.06 | -0.23 | 0.02 | 0.005 | 0.104 |
| South Asian vs Not South Asian | 0.07 | 1.05 | 0.06 | -0.06 | 0.19 | 0.002 | 0.296 |
| Black X South Asian | 0.03 | 0.48 | 0.06 | -0.09 | 0.15 | 0.000 | 0.633 |
| Harris vs. Other | 0.19 | 6.80 | 0.03 | 0.14 | 0.25 | 0.07 | <.001^***^ |
| POC vs. Not POC | -0.14 | -1.24 | 0.11 | -0.36 | 0.08 | 0.003 | 0.217 |
| Other vs. Harris X POC vs. Not  POC | 0.02 | 0.36 | 0.06 | -0.09 | 0.13 | 0.000 | 0.717 |
| Black vs Not Black X POC vs.  Not POC | 0.06 | 0.51 | 0.13 | -0.18 | 0.31 | 0.000 | 0.607 |
| South Asian vs Not South Asian  X POC vs. Not POC | -0.11 | -0.89 | 0.13 | -0.36 | 0.13 | 0.001 | 0.373 |
| Black X South Asian X POC vs.  Not POC | -0.06 | -0.50 | 0.13 | -0.31 | 0.18 | 0.000 | 0.619 |

*Note.*  CI = confidence interval; *LL* = lower limit; *UL* = upper limit. * *p* < .05, ** *p* < .01, *** *p* < .001.

## Table 67. Study 2 Inferential Statistics for Superior/Inferior by Condition and Race

| Effect | Estimate | *t* | *SE* | 95% CI | | η²ₚ | *p* |
| --- | --- | --- | --- | --- | --- | --- | --- |
|  |  |  |  | *LL* | *UL* |  |  |
| Fixed effects |  |  |  |  |  |  |  |
| Intercept | 3.29 | 66.11 | 0.05 | 3.19 | 3.39 | 0.883 | <.001^***^ |
| Black vs Not Black | -0.08 | -1.46 | 0.06 | -0.19 | 0.03 | 0.004 | 0.145 |
| South Asian vs Not South Asian | 0.09 | 1.62 | 0.06 | -0.02 | 0.20 | 0.005 | 0.105 |
| Black X South Asian | 0.03 | 0.58 | 0.06 | -0.08 | 0.14 | 0.001 | 0.560 |
| Harris vs. Other | 0.09 | 3.71 | 0.03 | 0.04 | 0.14 | 0.02 | <.001^***^ |
| POC vs. Not POC | -0.14 | -1.42 | 0.10 | -0.34 | 0.06 | 0.003 | 0.158 |
| Other vs. Harris X POC vs. Not  POC | 0.02 | 0.49 | 0.05 | -0.07 | 0.12 | 0.000 | 0.624 |
| Black vs Not Black X POC vs. Not  POC | -0.03 | -0.23 | 0.11 | -0.24 | 0.19 | 0.000 | 0.822 |
| South Asian vs Not South Asian X  POC vs. Not POC | -0.14 | -1.30 | 0.11 | -0.36 | 0.07 | 0.003 | 0.196 |
| Black X South Asian X POC vs. Not  POC | -0.03 | -0.30 | 0.11 | -0.25 | 0.19 | 0.000 | 0.766 |

*Note.*  CI = confidence interval; *LL* = lower limit; *UL* = upper limit. * *p* < .05, ** *p* < .01, *** *p* < .001.

## Table 68. Study 2 Inferential Statistics for American/Foreign by Condition and Race

| Effect | Estimate | *t* | *SE* | 95% CI | | η²ₚ | *p* |
| --- | --- | --- | --- | --- | --- | --- | --- |
|  |  |  |  | *LL* | *UL* |  |  |
| Fixed effects |  |  |  |  |  |  |  |
| Intercept | 4.24 | 89.22 | 0.05 | 4.15 | 4.33 | 0.932 | <.001^***^ |
| Black vs Not Black | 0.00 | 0.01 | 0.05 | -0.10 | 0.11 | 0.000 | 0.994 |
| South Asian vs Not South Asian | 0.01 | 0.15 | 0.05 | -0.10 | 0.11 | 0.000 | 0.881 |
| Black X South Asian | -0.06 | -1.10 | 0.05 | -0.16 | 0.05 | 0.002 | 0.273 |
| Harris vs. Other | 0.07 | 3.01 | 0.02 | 0.03 | 0.12 | 0.015 | 0.003^**^ |
| POC vs. Not POC | 0.07 | 0.76 | 0.10 | -0.11 | 0.26 | 0.001 | 0.446 |
| Other vs. Harris X POC vs. Not  POC | 0.02 | 0.37 | 0.05 | -0.08 | 0.11 | 0.000 | 0.713 |
| Black vs Not Black X POC vs.  Not POC | 0.01 | 0.09 | 0.11 | -0.20 | 0.22 | 0.000 | 0.927 |
| South Asian vs Not South Asian  X POC vs. Not POC | -0.12 | -1.17 | 0.11 | -0.33 | 0.09 | 0.002 | 0.244 |
| Black X South Asian X POC vs.  Not POC | 0.07 | 0.67 | 0.11 | -0.14 | 0.28 | 0.001 | 0.502 |

*Note.*  CI = confidence interval; *LL* = lower limit; *UL* = upper limit. * *p* < .05, ** *p* < .01, *** *p* < .001.
